# Switching Enantioselectivity in Phenylalanine Ammonia Lyase for the Synthesis of Electron‐Deficient Aromatic d‐Amino Acids

**DOI:** 10.1002/anie.202511739

**Published:** 2025-09-12

**Authors:** Ivan Buslov, Sarah Desmons, Weijin Wang, Laura Elena Massaad, Xile Hu

**Affiliations:** ^1^ Laboratory of Inorganic Synthesis and Catalysis Institute of Chemical Sciences and Engineering École Polytechnique Fédérale de Lausanne ISIC‐LSCI, BCH 3305 Lausanne 1015 Switzerland; ^2^ National Centre of Competence in Research (NCCR) Catalysis École Polytechnique Fédérale de Lausanne (EPFL) Lausanne 1015 Switzerland

**Keywords:** Asymmetric hydroamination, Biocatalysis, D‐amino acids, Enzyme engineering, Phenylalanine ammonia lyase (PAL)

## Abstract

Aromatic d‐amino acids (d‐AAs) are valuable building blocks in drug discovery and peptide therapeutics, yet their direct and efficient biocatalytic synthesis remains a challenge. Here, we report the rational engineering of phenylalanine ammonia lyase from *Planctomyces brasiliensis* (*Pb*PAL) to enable asymmetric hydroamination for the enantioselective synthesis of d‐aromatic amino acids. By targeting active‐site residue L205, we identified variants capable of highly d‐enantioselective hydroamination, with L205F enabling the transformation of electron‐deficient aryl acrylates with >99% enantiomeric excess (*ee*). The synthetic utility of this platform was demonstrated by gram‐scale synthesis of d‐benzoxazole and substituted 2‐pyridylalanines. Structural and mutational studies revealed distinct roles for the 4‐methylideneimidazole‐5‐one (MIO) prosthetic group and active‐site residues L205, Y64, and K397 in modulating enantioselectivity. These results enabled the identification of *Pb*PAL variants with the opposite selectivity, such as L205V‐K397A, which preferentially produce l‐amino acids. This work broadens the utility of PALs as programmable biocatalysts for asymmetric synthesis.

## Graphical abstract

Phenylalanine ammonia lyases (PALs) were engineered to switch enantioselectivity, enabling hydroamination for the direct synthesis of electron‐deficient aromatic d‐amino acids (d‐AA). Structure‐guided engineering provided PAL variants with broad substrate scope, high activity, and excellent d‐selectivity, broadening the utility of PALs in asymmetric synthesis.

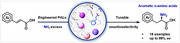

d‐Amino acids (d‐AAs) are considerably scarcer in nature compared to their l‐counterparts, yet they play crucial roles in both biological systems and pharmaceutical applications.^[^ ^1^, ^2^, ^3^, ^4^, ^5^ ^]^ Several marketed small‐molecule drugs, including tadalafil and nateglinide, incorporate aromatic d‐AA as essential structural motifs.^[^ ^6^, ^7^ ^]^ Moreover, d‐configured building blocks can significantly enhance chemical diversity and improve the pharmacological properties of therapeutic peptides.^[^ ^8^, ^9^, ^10^, ^11^ ^]^ Additionally, d‐AAs are present in naturally occurring non‐ribosomal peptides (NRPs)^[^ ^12^ ^]^ and in many natural antibiotics, which often display potent antimicrobial activity.^[^ ^9^ ^]^

Aromatic d‐AAs can be synthesized using chemical approaches such as chiral resolution and asymmetric catalysis.^[^ ^13^, ^14^ ^]^ However, these methods often require resource‐intensive steps involving protection groups, transition‐metal catalysts or chiral auxiliaries, and employing hazardous chemicals, posing notable environmental concerns. To address these issues, several biocatalytic strategies have been employed in the synthesis of d‐AAs.^[^ ^1^, ^3^, ^4^, ^15^ ^]^ One approach relies on the resolution of racemic mixtures through enantioselective degradation, employing enzymes such as hydrolases, phenylalanine ammonia lyases (PALs), or l‐amino acid oxidases (LAAOs). However, these methods only provide a maximum yield of 50% due to the kinetic resolution strategy (Figure 1a). Another synthetic strategy is dynamic kinetic resolution (DKR), where a racemic mixture is racemized while one enantiomer is selectively converted. The hydantoinase process remains one of the most established examples for producing optically pure d‐AAs (Figure 1b).^[^ ^16^ ^]^ Other reported multienzymatic DKRs that use acylases,^[^ ^17^ ^]^ oxidases,^[^ ^18^ ^]^ or dehydrogenases^[^ ^19^ ^]^ often share the hydantoinase process's suboptimal atom efficiency.^[^ ^20^ ^]^ A third approach involves the asymmetric biocatalytic transamination of pyruvates, using d‐amino acid aminotransferases (DAATs)^[^ ^21^, ^22^, ^23^, ^24^, ^25^ ^]^ or d‐amino acid dehydrogenases (DAADHs) (Figure 1c).^[^ ^26^, ^27^, ^28^, ^29^, ^30^ ^]^ While offering high stereoselectivity, the applications of these enzymes require cofactor regeneration systems (pyridoxal 5′‐phosphate (PLP) for DAATs and nicotinamide adenine dinucleotide phosphate (NADPH) for DAADHs). Moreover, the availability of corresponding pyruvates, which are typically derived from racemic amino acids, can be limited.^[^ ^31^, ^32^ ^]^

**Figure 1:**
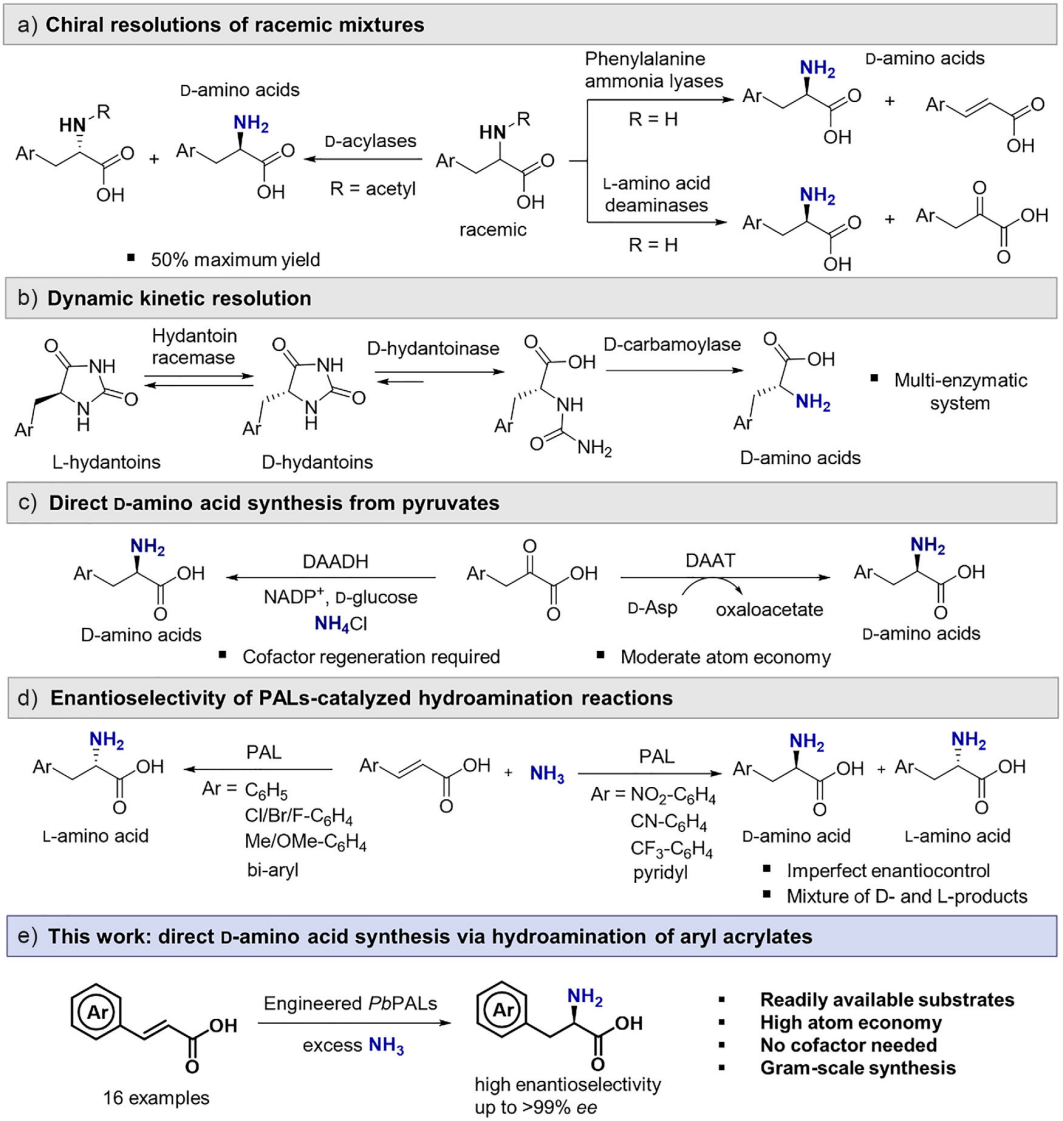
Enzymatic strategies for synthesizing aromatic d‐AAs. a) Methods relying on the resolution of racemic amino acid mixtures. b) DKR. c) Transamination and reductive amination of pyruvates using DAATs and DAADHs. d) Multienzymatic cascade process using PALs. e) This work: direct hydroamination of aryl acrylic acids using engineered *Pb*PALs.

The asymmetric hydroamination of alkenes using ammonia, mediated by ammonia lyases, provides a highly selective route to chiral amino acids. This reaction employs readily available substrates and reagents, and operates without requiring cofactors. As a result, it provides a potentially atom‐efficient, cost‐effective, and scalable synthetic strategy. Despite the advantages promised by PALs, they are usually highly selective in producing l‐enantiomers.^[^ ^33^, ^34^, ^35^, ^36^, ^37^, ^38^, ^39^ ^]^ Turner and co‐workers demonstrated that hydroamination of certain electron‐deficient substrates can produce substantial amounts of d‐amino acids, but high enantioselectivity was not achieved.^[^ ^32^, ^40^, ^41^ ^]^ The modifications in the carboxylate‐binding residues resulted in only a modest increase in the formation of the d‐enantiomers.^[^ ^31^ ^]^ High d‐selectivity from PAL has only been obtained when an additional enzyme, such as an l‐amino acid deaminase or oxidase, was employed to convert the undesired enantiomer (Figure 1d).^[^ ^31^, ^32^ ^]^

In this work, we report the rational engineering of the hydrophobic pocket in PAL from *Planctomyces brasiliensis* (*Pb*PAL) to control the enantioselectivity of hydroamination toward the selective formation of d‐AAs. This selectivity had not previously been obtained with a single PAL. Our engineered *Pb*PAL variants retain high catalytic activity and enable access to a range of synthetically valuable aromatic d‐AAs (Figure 1e). We elucidate the roles of key catalytic residues in *Pb*PAL affecting enantioselectivity of hydroamination of electron‐deficient substrates.

It is commonly accepted that PAL‐catalyzed hydroamination proceeds *via* anti‐addition, with the amino group delivered from the 4‐methylideneimidazole‐5‐one prosthetic group (MIO) side and protonation at the β‐carbon mediated by a catalytic tyrosine.^[^ ^42^, ^43^, ^44^, ^45^ ^]^ We previously demonstrated^[^ ^46^ ^]^ that the enantioselectivity of hydroamination was influenced by the nature and size of the residue at position 205 in *Pb*PAL, a leucine in the wild‐type, which is highly conserved among the MIO‐dependent enzymes.^[^ ^38^ ^]^ Based on that study, we hypothesized that modification at residue 205 of *Pb*PAL could alter the enantioselectivity of the amination reaction, enhancing d‐AA production (Figure 2a).

**Figure 2:**
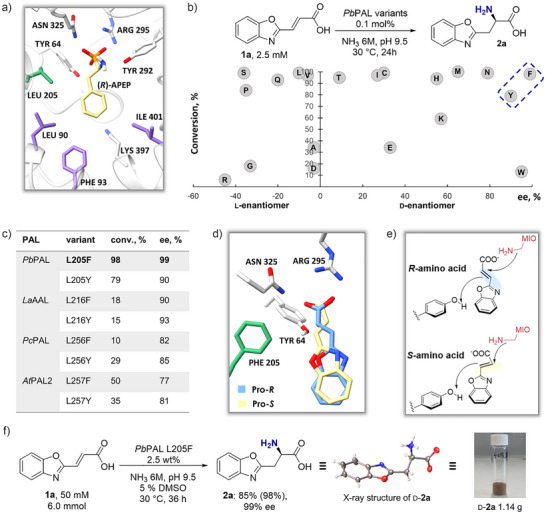
Active site residues, substrate positioning in the *Pb*PAL L205F variant, comparative analysis of site‐saturation mutagenesis at position L205, and gram‐scale synthesis of **2a**. a) Key active site residues of wild‐type *Pb*PAL (PDB: 9EQ5). b) Conversion and enantioselectivity for 20 *Pb*PAL L205X variants in the hydroamination under the following conditions: substrate **1a** (2.5 mM), 0.1 mol% enzyme loading, NH_3_ 6 M, pH 9.5, 30 °C, 24 h. c) Comparison of *Pb*PAL L205F with homologous mutations in *Pc*PAL, *At*PAL2, and *La*AAL in hydroamination of **1a**. d) Binding poses of pro‐(*R*) (blue) and pro‐(*S*) (yellow) conformations of substrate **1a** docked in the active site. e) Mechanistic representation of ammonia addition to electron‐deficient cinnamic acid derivative **1a**, showing formation of (*R*)‐ and (*S*)‐configured amino acids. f) Gram‐scale synthesis of **2a** was conducted on a 6.0 mmol scale (50 mM substrate, 6 M NH_3_, pH 9.5, 5% DMSO, 30 °C, 36 h, 2.5 wt% enzyme).

To test our hypothesis, we conducted a comprehensive screen of all 20 amino acid substitutions at position 205 of *Pb*PAL (Figure 2b and Table S8). Each variant was assessed in the hydroamination of substrate (*E*)‐3‐(benzo[d]oxazol‐2‐yl)acrylic acid (**1a**). Beyond serving as a tool to probe *Pb*PAL variants, the target product **2a** is a prominent pharmacophore, as benzoxazoles frequently appear in ligands for a broad range of receptors.^[^ ^47^, ^48^ ^]^ The reactions were carried out with 3 M ammonium carbonate as the sole ammonia source and 0.1 mol% PAL loading, calculated based on monomeric *Pb*PAL. Generally, substitutions with smaller residues than the native leucine (such as G, P, and S) favored the formation of the l‐amino acid **2a**, while bulkier residues like H, M, and N led to moderate enantiomeric excess (>50% *ee*) toward the d‐enantiomer (d‐**2a**). Charged residues (E, D, R) resulted in significantly reduced yields, likely due to unfavorable interactions in the active site. The L205W variant showed only 13% conversion, albeit with high enantioselectivity (95% *ee* for d‐**2a**). Among all variants, F and Y were identified as optimal: L205F gave 98% conversion with 99% *ee* for d‐**2a**, while L205Y yielded 79% conversion with 90% *ee*. The enantiomeric excess of the d‐**2a** increased during the initial phase of the *Pb*PAL L205F‐catalyzed hydroamination and remained high over 72 h (Figure S10). Notably, hydroamination of cinnamic acid with all *Pb*PAL variants resulted exclusively in l‐phenylalanine, highlighting the requirement for electron‐poor acrylic acids to achieve d‐selectivity in this system.

To explore whether related PAL homologs exhibit similar selectivity with substrate **1a**, we extended our study to other aromatic ammonia lyases of synthetic interest, specifically from *Petroselinum crispum* (*Pc*PAL L256F and L256Y),^[^ ^33^, ^34^, ^49^, ^50^ ^]^ *Arabidopsis thaliana* (*At*PAL2 L257F and L257Y),^[^ ^51^, ^52^ ^]^ and bacterial *Loktanella atrilutea* (*La*AAL L216F and L216Y)^[^ ^37^ ^]^ (Figure 2c and Table S9). Among these ammonia lyases, the best performing variant was *At*PAL2 L257F, which afforded 50% conversion and 77% *ee* for d‐**2a**. However, none of the tested homologs surpassed the catalytic efficiency and enantioselectivity of *Pb*PAL L205F. Thus, *Pb*PAL was selected as the parent enzyme for further biocatalytic transformations and engineering.

To gain insights, we performed molecular docking of substrate **1a** into the *Pb*PAL L205F variant active site, which was prepared from the cryo‐EM structure of *Pb*PAL (PDB: 9EQ5), using AutoDock Vina. The results supported our experimental results, showing that the pro‐(*R*) conformation, which corresponds to the d‐enantiomer, exhibited a binding affinity of −6.1 kcal mol^−1^, compared to −4.3 kcal mol^−1^ for the pro‐(*S*) (l‐form) conformation (Figure 2d). This result suggests that bulkier side chains at position 205 may preferentially favor d‐AA formation (Figure 2e). The preference for the pro‐(*R*) pose is likely due to favorable π–π and hydrophobic interactions of the benzoxazole ring with F205, whereas the alternative pro‐(*S*) orientation may be hindered by unfavorable steric interaction of the substrate's γ‐carbon with F205. To showcase the preparative utility of the system, the synthesis of d‐**2a** was performed on a gram scale using 2.5 wt% of *Pb*PAL L205F, affording the product in 85% isolated yield and 99% *ee*. The absolute configuration, corresponding to the (*R*)‐enantiomer (d‐**2a**), was confirmed by single‐crystal X‐ray diffraction analysis (Figure 2f).

With the above results in hand, we next evaluated the hydroamination of the structurally related benzothiazole‐derived acrylate **1b**. In this case, the *Pb*PAL L205Y variant outperformed L205F, delivering d‐**2b** with moderate isolated yield and 95% *ee* (Figure 3 and Table S10). Given that substitution patterns on the aromatic ring can influence both conversion and enantioselectivity, we next examined methyl‐substituted derivatives of **1a** at the 4‐, 5‐, 6‐, and 7‐positions (**1c‐1f**). We selected *Pb*PAL L205F as a benchmark and evaluated additional mutations targeting residues located near the phenyl ring of the substrate, namely F93V and I401V (Figure 3 and Table S11). For 4‐ and 7‐methyl‐substituted substrates, the original L205F variant retained superior performance. In contrast, for substrates bearing methyl groups at positions 5 and 6, variants L205F‐F93V and L205F‐I401V afforded satisfying conversion while maintaining moderate stereoselectivity. Semi‐preparative‐scale reactions were carried out for selected derivatives, yielding the corresponding products **2c‐2f** in good isolated yields. However, the enantiomeric excesses were reduced relative to d‐**2a** and d‐**2b**.

**Figure 3:**
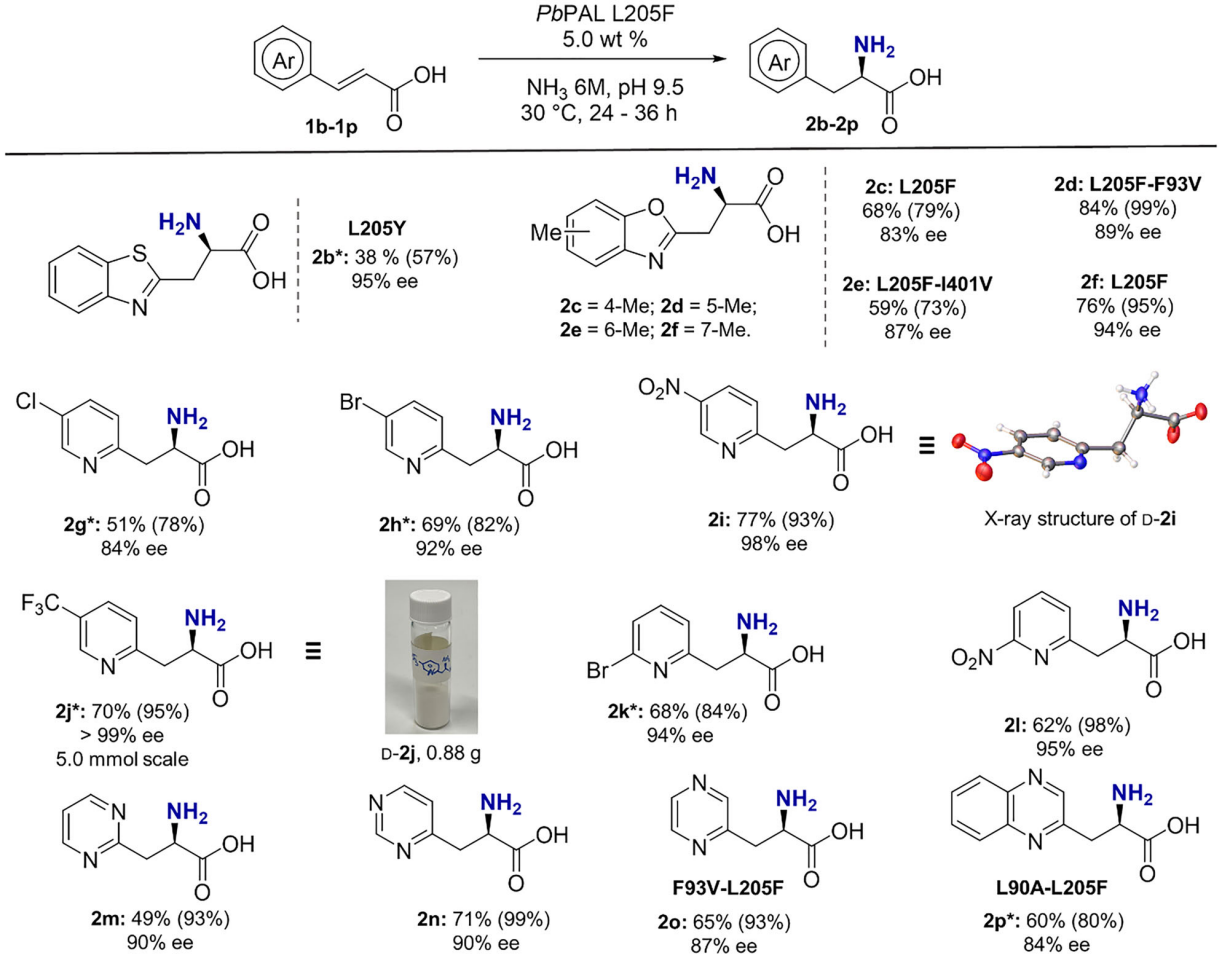
Synthesis of aromatic d‐AAs using *Pb*PAL variants. Experimental conditions: 0.1 mmol scale, 50 mM substrate, 5.0 wt% PAL, 6 M NH_3_, pH 9.5, 5% DMSO, 30 °C, 24 h (**2g‐2l**), 36 h (**2b‐2f**, **2p**), isolated yields. Conversions (in parentheses) determined by HPLC (**2b‐2l**, **2p**) and by ^1^H NMR (**2m‐2o**). Enantiomeric excesses were determined by HPLC; gram‐scale synthesis of **2j** was conducted on a 5.0 mmol scale using 50 mM substrate, 10.0 wt% PAL, 6 M NH_3_, pH 9.5, 5% DMSO, 30 °C, 24 h;* 10.0 wt% PAL loading.

We next targeted biocatalytic synthesis of d‐pyridylalanines, a valuable class of electron‐deficient arylalanines with applications in drug discovery and peptide design. Pyridylalanines are known to enhance solubility, stability, and reduce aggregation while maintaining biological activity.^[^ ^53^, ^54^ ^]^ Their incorporation improves pharmacokinetics^[^ ^55^ ^]^ and has also enabled enantioselective transformations in asymmetric catalysis.^[^ ^56^, ^57^ ^]^ A series of substituted 2‐pyridylacrylic acids were subjected to hydroamination under standard conditions. Substrates substituted in the 5‐position of the pyridyl ring, including 5‐chloro‐, 5‐bromo‐, 5‐nitro‐, and 5‐trifluoromethyl‐2‐pyridylacrylic acids (**1g‐1j**), as well as 6‐bromo‐ and 6‐nitro‐2‐pyridylacrylic acids (**1k** and **1l**), were converted with high enantioselectivity (84%–>99% *ee*) and good isolated yields (Figure 3). The (*R*)‐configuration of d‐**2i** was confirmed by single‐crystal X‐ray diffraction analysis. Preparative‐scale synthesis of d‐**2j** (5.0 mmol scale) was achieved with >99% *ee* and a 70% isolated yield using 10.0 wt% *Pb*PAL L205F, demonstrating the practical applicability. To further expand the substrate scope, we evaluated diazines, including pyrimidine (**1m** and **1n**), pyridazine (**1o**), and fused heterocycles, such as 2‐quinoxaline acrylic acid (**1p**).^[^ ^58^ ^]^ Pyrimidine substrates **1m** and **1n** were well tolerated, affording products with 90% *ee*. Using L205F‐F93V and L205F‐L90A variants enabled the synthesis of more challenging substrates (**1o** and **1p**) with moderate yields and enantioselectivities of 87% and 84%, respectively (Figure 3). Kinetic measurements confirmed that *Pb*PAL L205F maintains high catalytic activity under hydroamination conditions (see Section S4).

It is worth noting that the reaction of unsubstituted (*E*)‐3‐(pyridin‐2‐yl)acrylic acid gave the product in 95% *ee*, though the conversion was modest with *Pb*PAL L205F and L205Y and homologs from *At*PAL2, *Pc*PAL, and *La*AAL (Figure S11 and Table S15). Substrates bearing electron‐donating groups, such as methyl or methoxy substituents, were not accepted by the engineered enzymes. 3‐ and 4‐chloro‐substituted pyridylacrylates also exhibited reduced reactivity (Figure S11). In contrast, the reactions of (*E)*‐3‐(2‐bromopyridin‐4‐yl)acrylic acid, 4‐cyano‐, 4‐nitrocinnamic acids, and 2‐bromo‐4‐nitrocinnamic acids, despite having a high conversion, yielded products with <20% *ee*. These data highlight the important role of the iminium nitrogen at the δ‐position of the substrate in achieving high enantioselectivity.

We sought to probe the mechanistic basis for the observed enantioselectivity by studying key active‐site residues engaged in the PAL catalytic cycle.^[^ ^59^, ^60^, ^61^, ^62^ ^]^ First, we examined the role of the MIO prosthetic group^[^ ^45^ ^]^ by employing the *Pb*PAL S154A variant as a catalyst, which lacks the ability to generate MIO. A complete loss of hydroamination activity toward 2‐pyridylacrylic acid **1k** and benzoxazole acrylic acid **1a** confirmed that the MIO‐dependent mechanism is essential for d‐AA formation in this system (Figure 4a), ruling out the previously suggested MIO‐independent hydroamination pathway.^[^ ^40^ ^]^

**Figure 4:**
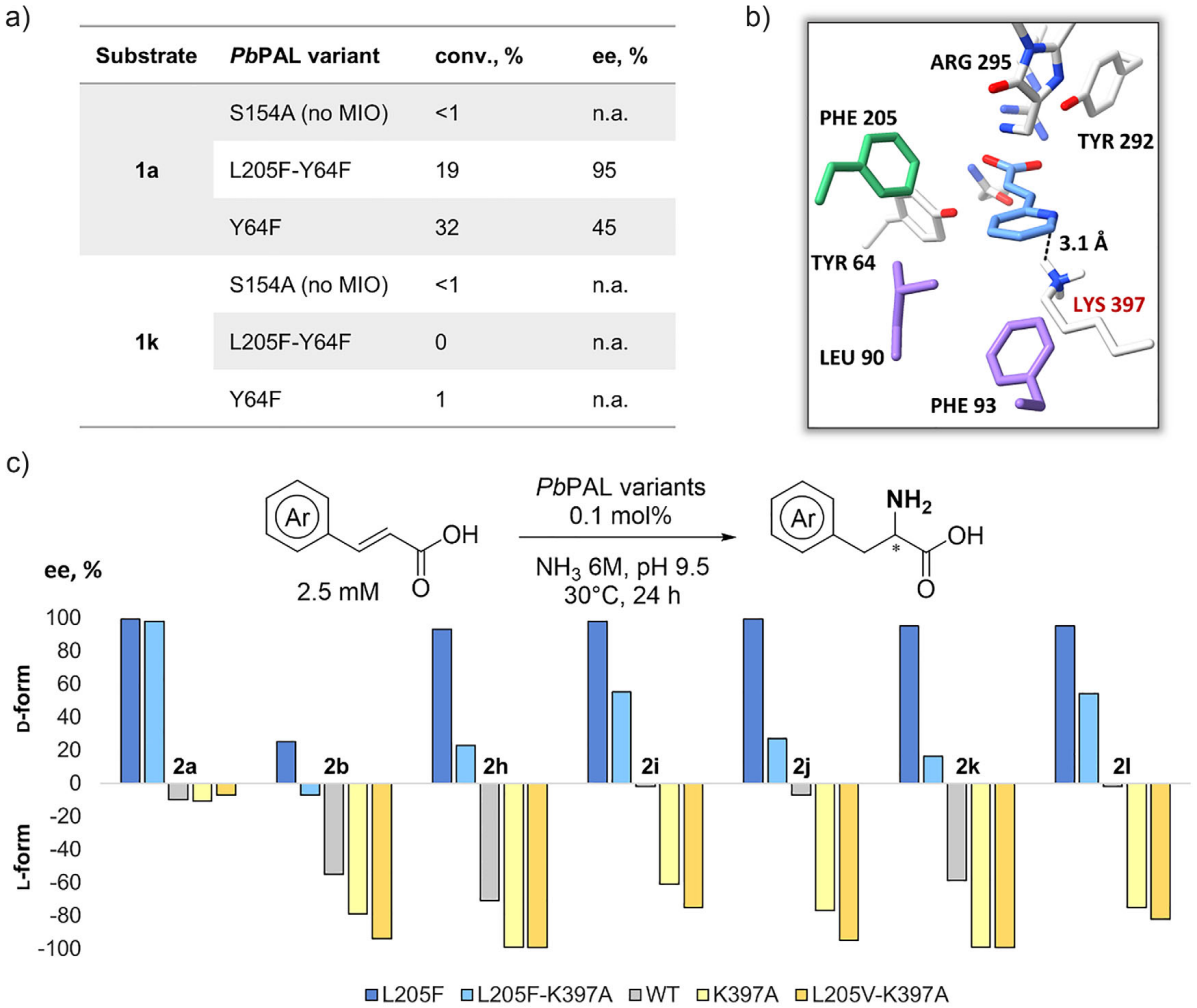
Mechanistic insights into the enantioselectivity control in *Pb*PAL‐catalyzed hydroamination. a) Hydroamination activity and enantioselectivity of MIO‐deficient *Pb*PAL S154A, mutated catalytic tyrosine Y64F, and double mutant L205F‐Y64F variants toward substrates **1a** and **1k**. b) QM‐minimized model of the *Pb*PAL L205F complex with (*E*)‐3‐(pyridin‐2‐yl)acrylic acid showing a potential interaction between K397 and the imino‐nitrogen of the substrate. c) Enantioselectivity of *Pb*PAL variants in the hydroamination of **1a‐1b** and **1h‐1l**. Experimental conditions: 2.5 mM substrate, 0.1 mol% PAL, 6 M NH_3_, pH 9.5, 30 °C, 24 h.

We next examined the conserved catalytic tyrosine residue Y64, which is assumed to act as an essential proton donor in the hydroamination. Surprisingly, the L205F‐Y64F variant retained considerable activity and exhibited similar enantioselectivity to the L205F variant in the hydroamination of **1a**, suggesting that an alternative proton transfer mechanism may be operative. Notably, the Y64F single mutant displayed moderate activity producing d‐**2a** with moderate 50% *ee*, providing evidence that disruption of the canonical pathway allows for the emergence of an alternative, d‐selective route. However, for the 2‐pyridyl substrate **2k**, a complete loss of reactivity of *Pb*PAL L205F‐Y64F and Y64F variants was observed, indicating that Y64 remains crucial for the hydroamination of this type of electron‐deficient substrates (Figure 4a).

To identify additional residues involved in modulating enantioselectivity, we performed QM minimization of the *Pb*PAL L205F complex with (*E*)‐3‐(pyridin‐2‐yl)acrylic acid. The analysis of the structure revealed that the lysine residue K397 (conserved among MIO‐dependent enzymes) is optimally positioned to form a hydrogen bond with the imino‐nitrogen of the substrate, suggesting a potential role in inducing stereochemical outcome (Figure 4b). Supporting this, the L205F‐K397A mutant displayed diminished d‐selectivity relative to L205F (Figure 4c and Table S19).

Building on these insights, we hypothesized that the disruption of the K397‐assisted d‐pathway could be exploited to favor l‐amino acid formation. To test this, we evaluated the *Pb*PAL L205V‐K397A double mutant, which combines a smaller hydrophobic residue at position 205 with the elimination of the K397 side chain. This combination was predicted to favor the l‐selective pose. Indeed, compared to the wild‐type PAL and K397A, the L205V‐K397A variant achieved high conversion and excellent enantioselectivity (up to >99% *ee*) toward the l‐isomers across a panel of arylacrylic acids (**1a** and **1b**, **1h**‐**1l**) (Figure 4c, Table S19). To showcase its synthetic utility, hydroamination of substrate **1k** at 50 mM was accomplished using 5.0 wt% *Pb*PAL L205V‐K397A, delivering the corresponding l‐**2k** in 61% isolated yield and >99% *ee*. Notably, this level of l‐selectivity was not achieved in wild‐type PALs.^[^ ^41^ ^]^ These results highlight how active‐site design precisely controls stereochemical outcomes and demonstrate the tunability of *Pb*PAL to achieve both d‐ and l‐selectivity.

In summary, this work establishes a direct biocatalytic hydroamination route for accessing valuable electron‐deficient aromatic d‐AAs, overcoming the limitations of existing chemical and enzymatic methods. Through structure‐guided mutagenesis at key active‐site positions, most notably L205, we achieved remarkable improvements in enantioselectivity toward a broad range of synthetically valuable substrates. The engineered variants enabled preparative‐scale synthesis of d‐AAs with enantiomeric excesses up to >99%. Mechanistic insights into the roles of the active‐site residues such as MIO, Y64, L205, and K397 advanced our understanding of enantioselectivity control and provided new avenues for tuning the enantioselectivity of PALs. The engineered *Pb*PAL variants represent robust biocatalysts with broad synthetic utility and set the stage for future applications in the sustainable synthesis of chiral building blocks.

## Supporting Information

The authors have cited additional references within the Supporting Information.^[^ ^63^, ^64^, ^65^, ^66^, ^67^, ^68^, ^69^, ^70^, ^71^, ^72^, ^73^ ^]^

Experimental procedures and compound characterization data that support the findings of this study are available in the online version of this paper in the accompanying Supporting Information. Crystallographic data for **2a** and **2i** have been deposited at the Cambridge Crystallographic Data Centre, under deposition numbers CCDC 2410915 (**2a**), CCDC 2410916 (**2i**). Copies of the data can be obtained free of charge *via* www.ccdc.cam.ac.uk. Further data are available online in Zenodo: doi: 10.5281/zenodo.16849579

## Conflict of Interests

The authors declare no conflict of interest.

## Supporting information



Supporting Information

## Data Availability

The data that support the findings of this study are available in the supplementary material of this article.
