# Supplementary material for: Switching Enantioselectivity in Phenylalanine Ammonia Lyase for the Synthesis of Electron‐Deficient Aromatic d‐Amino Acids
Source: Angew Chem Int Ed Engl. 2025 Sep 12;64(43):e202511739. doi: 10.1002/anie.202511739 (PMC12535398; doi:10.1002/anie.202511739)
Supplement: Supplementary file 1 — Supporting Information [file ANIE-64-e202511739-s001.pdf]

Table of Contents

1. GENERAL EXPERIMENTAL DETAILS 2

2. INSTRUMENTATION AND PRODUCT CHARACTERIZATION 2

3. MOLECULAR BIOLOGY PROTOCOLS 5

4. ENZYME KINETIC CHARACTERIZATION 11

5. SYNTHESIS OF ACRYLIC ACIDS 16

6. ANALYTICAL SCALE HYDROAMINATIONS 18

7. PREPARATIVE AND SEMI-PREPARATIVE SCALE HYDROAMINATIONS 25

8. X-RAY CRYSTALLOGRAPHY 33

9. MOLECULAR DOCKING SIMULATIONS 37

10. QM CLUSTER CALCULATION 38

11. REFERENCES 46

12. HPLC CHROMATOGRAMS 47

13. ^{1}H, ^{13}C AND ^{19}F NMR SPECTRA OF COMPOUNDS 2A-2P 64

1. General Experimental Details

Chemicals and materials

Ethyl acetate and acetonitrile (HPLC gradient grade) were purchased from Fisher Chemical. Ammonium carbonate was purchased from Sigma-Aldrich. (*E*)-3-(benzo[d]oxazol-2-yl)acrylic acid (**1a**), (*E*)-3-(benzo[d]thiazol-2-yl)acrylic acid (**1b**) were purchased from Combi-Blocks and used as received. Maleic anhydride, polyphosphoric acid, 2-amino-3-methylphenol, 2-amino-4-methylphenol, 2-amino-5-methylphenol, 2-amino-6-methylphenol were purchased from Sigma-Aldrich. Malonic acid was purchased from Apollo scientific and used as received. Picolinaldehydes, isonicotinaldehydes, pyrimidinecarbaldehydes, pyrazine-2-carbaldehyde, quinoxaline-2-carbaldehyde and N_{α}-(2,4-Dinitro-5-fluorophenyl)-L-valine amide (L-FDVA) were purchased from Fluorochem. All deuterated solvents were purchased from Sigma-Aldrich and used without further purification. All other reagents were purchased from Sigma-Aldrich and used as received.

2. Instrumentation and product characterization

NMR spectroscopy

NMR spectra (^{1}H, ^{13}C{^{1}H}, ^{19}F) were recorded at 293 K on a Bruker Advance 400 Spectrometer. ^{1}H and ^{13}C chemical shifts are given in ppm and were referenced internally to residual solvent resonances or methanol-*d₄* (4.79 ppm for ^{1}H spectra (D_2O residual solvent peak), 49.00 ppm for ^{13}C spectra (chemical shift of added methanol-d_4)).
Coupling constants (*J*) are reported in Hertz (Hz), and the following abbreviations for signal multiplicities and description were used: s = singlet, d = doublet, t = triplet, q = quartet, m = multiplet, br = broad signal.
The chemical shift of all signals is reported as the center of the resonance range, except in the case of multiplets, which are reported as ranges in chemical shift. All raw fid files were processed and the spectra analyzed using the program MestReNOVA 14.3.0 from Mestrelab Research S. L.

Automated column purification

Automated column chromatography was performed on an Biotage® Isolera™ One 3.3.0 apparatus using Biotage® Stär Silica D Duo 60 µm columns.

High-resolution mass spectrometry (HRMS) analyses

High-resolution mass spectrometry (HRMS) was performed by the EPFL ISIC Mass Spectroscopy Service employing electrospray ionization (ESI).

Optical rotation data

Optical rotations were measured on a Polartronic M polarimeter using a 0.5 cm cell with a Na 589 nm filter.

HPLC analyses

High performance liquid chromatography (HPLC) analyses were conducted with Agilent 1260 (Santa Clara, CA, USA) and UltiMate 3000 (Thermo Fisher Scientific, Waltham, MA, USA) systems.

Solvent compositions used in the LC are 0.1% formic acid in H_2O (solvent A) and 0.1% formic acid in acetonitrile (solvent B). The following LC-MS methods have been used:

Method A LC conditions: Agilent Zorbax Eclipse Plus C18 column: 4.6 × 100 mm, 3.5 µm, column temperature: 25 °C, gradient: 0-2 min 1% B, 2-11 min 1-80% B, 11-12 min 80-95% B, flow rate: 0.8 mL/min.

The conversions of compounds **1a-l** to **2a-l** and **1p** to **2p** were determined using **Method A** detected at 210 nm wavelength (retention times and relative response factors listed below).

Table S1. Retention times and response factors of **1a**-**1l** and **1p**-**2p**.

Substrate	Retention time (t_R), min		Response factors 1 vs 2
	1	2	
**a**	9.3	6.4	1.23
**b**	10.5	7.2	1.66
**c**	11.1	7.9	1.42
**d**	11.1	7.9	1.33
**e**	11.0	7.8	1.50
**f**	10.9	7.8	1.42
**g**	9.9	6.6	1.17
**h**	10.1	6.9	1.60
**i**	9.5	4.4	0.73
**j**	10.6	7.4	1.11
**k**	10.1	6.9	1.52
**l**	9.5	4.4	0.81
**p**	9.8	7.1	0.97

Chiral stationary phase (determination of enantiomeric excesses of 2a-2l, and 2p)

For separating enantiomers of products **2a-2f,** solvent compositions consisted of 11.4 mM $HClO_4$ in H_2O pH 2.0 (solvent A) and acetonitrile (solvent B). The following LC-MS methods were used:

Method B LC conditions: Daicel Corporation, CROWNPACK CR-I (+), 3.0 × 150 mm, 5 mm, column temperature: ambient, gradient: 0-2 min 1% B, 2-12 min 1-30% B, 11-16 min 30-95% B, flow rate: 0.3 mL/min, detected at 280 or 254 nm wavelength.

Method C LC conditions: Daicel Corporation, CROWNPACK CR-I (+), 3.0 × 150 mm, 5 mm, column temperature: ambient, gradient: 0-2 min 1% B, 2-12 min 1-40% B, 12-22 min 40-95% B, flow rate: 0.3 mL/min, detected at 254 nm wavelength.

Table S2. Retention times of D- and L- enantiomers of **2a**-**2f**.

Substrate	Retention time (t_R), min[a]		HPLC Method
	**D-2**	**L-2**	
**a**	4.8	6.2	B
**b**	8.4	11.4	C
**c**	7.6	9.2	B
**d**	7.7	10.0	B
**e**	7.9	10.1	B
**f**	9.7	13.3	B

[a]Retention times for the D- and L-enantiomers were assigned by analyzing product mixture obtained from hydroamination catalyzed by wild-type *Pb*PAL.

For separating enantiomers of products **2g-2l** and **2p** solvent compositions consisted of 114 mM $HClO_4$ in H_2O pH 1.0 (solvent A) and methanol (solvent B). The following LC-MS methods were used:

Method D LC conditions: Daicel Corporation, CROWNPACK CR-I (+), 3.0 × 150 mm, 5 mm, column temperature: ambient, isocratic elution: 0-12 min 4% B, flow rate: 0.4 mL/min, detected at 280 or 254 nm wavelength.

Method E LC conditions: Daicel Corporation, CROWNPACK CR-I (+), 3.0 × 150 mm, 5 mm, column temperature: ambient, isocratic elution: 0-30 min 4% B, flow rate: 0.4 mL/min, detected at 280 nm wavelength.

Table S3. Retention times of D- and L- enantiomers of **2g**-**2l** and **2p**.

**Substrate**	**Retention time (t_R), min[a]**		**HPLC Method**
	**D-2**	**L-2**	
**g**	4.0	5.2	D
**h**	4.8	6.6	D
**i**	4.3	5.8	D
**j**	4.5	6.4	D
**k**	4.7	6.3	D
**l**	4.2	5.8	D
**p**	7.2	10.9	E

[a]Retention times for the D- and L-enantiomers were assigned by analyzing product mixture obtained from hydroamination catalyzed by wild-type *Pb*PAL.

Procedure for FDVA derivatization and determination of enantiomeric excesses of 2m-2o

The absolute configurations of amino acids **2m-2o** were determined by derivatization using 1-fluoro-2,4-dinitrophenyl-5-L-valine amide (L-FDVA). Stock solutions of amino acid standards (25 mM in H_2O), $NaHCO_3$ (1 M in H_2O), and L-FDVA (37.5 mM in methanol) were prepared. Available standards and amino acids prepared *via* hydroamination were derivatized using molar ratios of amino acid to L-FDVA and $NaHCO_3$ (1:1.5:10). After incubating at 45 °C for 30 minutes, samples were subsequently analyzed using C18 reverse-phase HPLC-MS with the standard gradient (Method A) at a flow rate of 0.8 mL/min, detected at 330 nm wavelength.

Table S4. Retention times of L-FDVA-derivatized D- and L- enantiomers of **2m**-**2n**.

**Substrate**	**Retention time (t_R), min[a]**		**HPLC Method**
	**L-2**	**D-2**	
**m**	10.26	10.65	A
**n**	10.29	10.70	A
**o**	10.10	10.45	A

[a]Retention times for the D- and L-enantiomers were assigned by analyzing product mixture obtained from hydroamination catalyzed by wild-type *Pb*PAL.

3. Molecular biology protocols

Polypeptide sequences

The His9 tag, along with a short polypeptide, is indicated by underlining.

***Pc*PAL wt (C704S-C716S, His$_9$ tag):**

MENGNGATTNGHVNGNGMDFCMKTEDPLYWGIAAEAMTGSHLDEVKKMVAEYRKPVVKLGGETLTISQVAAISARDGSGVT
VELSEAARAGVKASSDWVMDSMNKGTDSYGVTTGFGATSHRRTKQGGALQKELIRFLNAGIFGNGSDNTLPHSATRAAMLVR
INTLLQGYSGIRFEILEAITKFLNQNITPCLPLRGTITASGDLVPLSYIAGLLTGRPNSKAVGPTGVILSPEEAFKLAGVEGGFFELQ
PKEGLALVNGTAVGSGMASMVLFEANILAVLAEVMSAIFAEVMQGKPEFTDHLTHKLKHHPGQIEAAAIMEHILDGSAYVKAAQ
KLHEMDPLQKPKQDRYALRTSPQWLGPQIEVIRSSTKMIEREINSVNDNPLIDVSRNKAIHGGNFQGTPIGVSMDNTRLAIAAIG
KLMFAQFSELVNDFYNNGLPSNLSGGRNPSLDYGFKGAEIAMASYCSELQFLANPVTNHVQSAEQHNQDVNSLGLISSRKTSE
AVEILKLMSTTFLVGLCQAIDLRHLEENLKSTVKNTVSSVAKRVLTMGVNGELHPSRFCEKDLLRVVDREYIFAYIDDPCSATYP
LMQKLRQTLVEHALKNGDNERNLSTSIFQKIATFEDELKALLPKEVESARAALESGNPAIPNRIEECRSYPLYKFVRKELGTEYLT
GEKVTSPGEEFEKVFIAMSKGEIIDPLLESLESWNGAPLPISSGGGLEVLFQGPGGSSGSGHHHHHHHHH

Number of amino acids = 744

Molecular weight = 80662.76 Da

pI = 6.05

***Pc*PAL wt (C704S-C716S) DNA sequence:**

ATGGAGAACGGGAACGGCGCCACAACGAATGGCCACGTTAATGGGAACGGTATGGACTTCTGTATGAAAACCGAA
GACCCGTTGTACTGGGGCATTGCTGCAGAAGCGATGACTGGCAGTCATCTTGATGAAGTTAAAAAGATGGTCGCTG
AGTACCGCAAACCTGTGGTTAAGTTAGGGGGGGAAACGCTTACAATTAGCCAAGTGGCTGCCATCTCTGCCCGGG
ACGGCTCAGGGGTGACGGTGGAGCTCTCTGAAGCTGCACGGGCTGGGGTCAAGGCCTCTAGCGACTGGGTTATG
GACTCAATGAACAAAGGGACGGACAGTTATGGGGTTACAACGGGTTTCGGCGCCACGTCTCATCGTCGGACCAAG
CAAGGGGGCGCTCTCCAGAAGGAGCTCATTCGCTTTCTTAATGCAGGGATCTTTGGGAATGGTTCCGATAACACTC
TCCCGCATTCCGCTACTCGCGCTGCTATGCTGGTTCGCATCAATACACTCTTGCAGGGCTACAGTGGCATCCGGTT
TGAAATTCTTGAAGCTATTACCAAATTCCTTAACCAAAACATCACCCCGTGTCTCCCATTACGGGGGACTATTACTG
CCAGCGGGGACTTGGTGCCACTGTCTTACATTGCTGGTCTCTTAACCGGCCGGCCGAATTCAAAGGCTGTGGGTC
CAACGGGCGTGATTTTGAGTCCTGAGGAAGCCTTTAAATTAGCTGGTGTCGAAGGCGGGTTTTTTGAACTGCAGCC
TAAGGAGGGCCTCGCTTTGGTCAATGGGACTGCGGTAGGCAGCGGGATGGCTTCGATGGTCCTTTTTGAAGCAAA
TATTCTCGCCGTGTTGGCCGAAGTAATGTCTGCTATTTTCGCAGAAGTAATGCAAGGCAAACCTGAGTTCACCGAT
CATCTCACACACAAGTTAAAACACCACCCAGGTCAGATTGAAGCCGCGGCTATTATGGAGCACATTCTCGACGGGA
GCGCATACGTTAAAGCCGCTCAGAAGCTGCACGAAATGGACCCGTTACAAAAACCGAAGCAAGACCGTTACGCGC
TGCGTACGTCGCCACAGTGGCTTGGGCCTCAAATTGAAGTGATCCGTTCGTCGACGAAGATGATCGAGCGTGAAA
TTAACTCTGTGAATGATAATCCGCTGATTGACGTCTCACGTAACAAGGCGATCCACGGCGGCAATTTTCAGGGCAC
GCCAATTGGGGTGAGTATGGACAACACTCGTTTGGCAATTGCCGCTATTGGCAAGCTGATGTTTGCTCAGTTTTCA
GAACTTGTTAATGACTTTTATAATAACGGCCTTCCGAGCAACCTGTCCGGCGGGCGCAATCCAAGCTTGGATTATG
GCTTTAAAGGCGCGGAAATCGCAATGGCCAGCTATTGTTCCGAACTTCAATTTTTAGCGAACCCGGTAACAAATCAT
GTACAGAGCGCCGAGCAGCATAACCAAGATGTTAACTCACTCGGCTTAATCTCTAGCCGTAAAACCTCGGAAGCAG
TCGAGATTCTGAAGTTAATGAGCACAACGTTCTTAGTCGGTCTCTGCCAAGCGATCGACCTCCGTCACCTTGAAGA
AAATCTTAAGTCAACAGTAAAGAACACGGTTAGCTCCGTAGCAAAACGGGTGCTGACTATGGGCGTGAATGGCGAA
CTTCATCCTTCACGCTTTTGCGAGAAGGACTTGTTGCGCGTCGTCGACCGGGAATATATTTTTGCCTATATTGACGA
CCCTTGCAGTGCTACGTATCCATTGATGCAAAAACTCCGCCAGACACTGGTGGAGCACGCCCTTAAGAACGGCGA
TAACGAACGCAATCTCTCAACGTCAATCTTTCAAAAAATCGCAACATTCGAGGACGAGTTAAAAGCGCTTCTGCCGA
AAGAGGTTGAATCTGCGCGTGCGGCATTGGAAAGTGGGAATCCTGCAATCCCAAACCGCATCGAAGAATGCCGTT
CGTACCCGCTTTATAAGTTTGTACGTAAAGAACTGGGTACCGAATATTTGACCGGCGAGAAAGTCACGTCACCTGG
CGAGGAGTTCGAAAAGGTTTTTATCGCCATGAGTAAAGGTGAAATCATTGACCCTTTACTCGAAAGTTTAGAGAGTT
GGAACGGTGCGCCACTCCCGATTTCC

***At*PAL2 wt (His$_9$ tag):**

MDQIEAMLCGGGEKTKVAVTTKTLADPLNWGLAADQMKGSHLDEVKKMVEEYRRPVVNLGGETLTIGQVAAISTVGGSVKVE
LAETSRAGVKASSDWVMESMNKGTDSYGVTTGFGATSHRRTKNGTALQTELIRFLNAGIFGNTKETCHTLPQSATRAAMLVR
VNTLLQGYSGIRFEILEAITSLLNHNISPSLPLRGTITASGDLVPLSYIAGLLTGRPNSKATGPDGESLTAKEAFEKAGISTGFFDL
QPKEGLALVNGTAVGSGMASMVLFEANVQAVLAEVLSAIFAEVMSGKPEFTDHLTHRLKHHPGQIEAAAIMEHILDGSSYMKLA
QKVHEMDPLQKPKQDRYALRTSPQWLGPQIEVIRQATKSIEREINSVNDNPLIDVSRNKAIHGGNFQGTPIGVSMDNTRLAIAAI
GKLMFAQFSELVNDFYNNGLPSNLTASSNPSLDYGFKGAEIAMASYCSELQYLANPVTSHVQSAEQHNQDVNSLGLISSRKTS
EAVDILKLMSTTFLVGICQAVDLRHLEENLRQTVKNTVSQVAKKVLTTGINGELHPSRFCEKDLLKVVDREQVFTYVDDPCSAT
YPLMQRLRQVIVDHALSNGETEKNAVTSIFQKIGAFEEELKAVLPKEVEAARAAYGNGTAPIPNRIKECRSYPLYRFVREELGTK
LLTGEKVVSPGEEFDKVFTAMCEGKLIDPLMDCLKEWNGAPIPICSGGGLEVLFQGPGGSSGSGHHHHHHHH

Number of amino acids = 745

Molecular weight = 80725.92 Da

pI = 6.22

***At*PAL2 wt DNA sequence:**

ATGGATCAAATCGAGGCTATGTTATGCGGCGGTGGCGAGAAGACTAAGGTTGCAGTGACTACAAAGACCTTGGCG
GACCCTTTAAACTGGGGACTGGCAGCGGATCAGATGAAGGGATCACATTTAGACGAAGTAAAGAAGATGGTAGAA
GAATATCGTCGCCCGGTAGTTAATTTGGGGGGAGAAACGCTTACGATTGGACAGGTCGCAGCGATTTCCACTGTTG
GTGGCTCTGTAAAAGTAGAATTAGCAGAAACGTCGCGCGCGGGTGTAAAGGCCTCTTCCGACTGGGTCATGGAGT
CGATGAATAAAGGTACAGACTCGTACGGGGTAACTACCGGATTTGGTGCTACATCTCATCGTCGCACGAAAAATGG
TACAGCGTTGCAGACGGAACTTATTCGTTTTTTGAATGCGGGCATCTTTGGTAACACTAAAGAGACATGTCACACCT
TACCCCAAAGTGCTACCCGCGCAGCGATGTTAGTTCGTGTTAACACTCTTTTGCAAGGATACAGTGGGATTCGTTT
CGAAATCTTAGAGGCGATTACCTCGCTTTTAAATCACAATATTTCGCCTAGCCTTCCGTTGCGTGGGACCATCACTG
CGTCTGGGGATTTGGTGCCATTGTCCTACATCGCGGGCCTTCTTACGGGCCGCCCGAATTCAAAGGCAACGGGAC
CTGACGGCGAATCGTTAACGGCTAAAGAGGCATTTGAAAAAGCCGGCATCTCTACGGGTTTTTTCGACCTGCAGCC
AAAAGAAGGATTAGCGTTAGTTAATGGTACGGCAGTTGGGAGTGGTATGGCGAGCATGGTGCTGTTTGAGGCTAA
CGTGCAGGCTGTTTTGGCTGAGGTATTGTCCGCGATTTTCGCTGAAGTAATGTCCGGCAAGCCTGAATTCACCGAC
CACCTTACACATCGTCTGAAACACCACCCGGGCCAAATTGAGGCCGCAGCAATTATGGAACATATTCTGGACGGCT
CATCGTACATGAAGCTGGCCCAAAAGGTCCACGAAATGGATCCCCTTCAGAAACCCAAACAGGATCGCTACGCGC
TTCGTACGTCTCCTCAATGGCTTGGCCCGCAAATCGAGGTAATTCGTCAAGCCACAAAGAGTATCGAACGTGAAAT
CAACTCAGTAAACGACAATCCGCTGATCGATGTAAGCCGCAATAAAGCGATCCATGGGGGGAATTTTCAAGGTACG
CCGATTGGGGTTAGCATGGACAACACGCGTTTAGCGATTGCGGCGATCGGAAAGCTTATGTTCGCGCAATTCTCA
GAACTTGTAAATGATTTCTACAACAATGGGTTGCCGTCTAATTTAACGGCATCCTCGAATCCGAGTCTTGACTATGG
CTTTAAGGGTGCAGAAATTGCCATGGCATCCTACTGTTCGGAGTTGCAATATTTAGCCAATCCCGTAACTTCTCATG
TGCAATCTGCCGAGCAGCACAACCAGGATGTAAACAGCCTTGGCTTGATCTCGTCACGCAAGACTTCGGAAGCCG
TGGACATTTTGAAATTAATGAGTACTACATTCCTTGTTGGGATCTGTCAAGCTGTCGACTTGCGCCATCTGGAAGAA
AATTTACGCCAGACTGTTAAGAACACTGTTAGTCAAGTGGCTAAGAAAGTTCTTACTACAGGTATTAACGGAGAACT
TCACCCTTCTCGTTTTTGCGAGAAAGACCTTTTAAAAGTTGTGGATCGCGAACAAGTTTTCACATACGTGGATGACC
CATGTAGCGCTACGTACCCACTTATGCAACGCTTACGTCAGGTTATTGTGGATCACGCGCTGAGTAATGGAGAGAC
CGAGAAAAACGCTGTAACGTCTATCTTCCAAAAAATTGGCGCGTTCGAAGAGGAACTGAAAGCCGTTCTTCCCAAG
GAAGTTGAAGCTGCGCGCGCAGCTTATGGGAACGGAACAGCTCCCATCCCCAATCGTATCAAGGAATGTCGCAGT
TACCCTTTGTACCGTTTCGTTCGTGAAGAGCTTGGAACCAAGTTGCTTACCGGGGAAAAAGTAGTCTCACCTGGAG
AAGAGTTTGATAAGGTGTTCACTGCCATGTGTGAGGGCAAGTTAATTGACCCACTGATGGACTGTCTTAAAGAGTG
GAACGGTGCTCCAATTCCCATTTGC

***Pb*PAL wt (His$_9$ tag):**

MLASSPSGHTNPVLSGAPLSINVVADIGRQRLIPSLTDDEQVLNRVHACRDVVQKAVRNNERIYGITTGFGGMSDIPIPPQHVA
QTQDNLLAFLSTSTGASLDPRHVRAAMALRANVLLQGRSGVRLELIERLVEFLRQDAIPVVCDLGSIGASGDLVPLGVIARSIIGH
PSTTQVKYQGEQADSHDVLQQLNYSALQLEAKEGLALVNGTSFSSAIAANCVFESQRLLSLSLVLQSIMVRALGGHPEAFHPF
VDENKPHPGQGWSAQMMRDLLSYSPNDSKRNGDLAQDRYSLRCLAQYFAPIVEGIAQISQSISTEMNAVSDNPLIDVDTGRFH
QSGNFLGQYVAMSMDQLRRHLGLLAKHLDVQIAQLVAPAFNNGLPASLRGNSSRPFNMGLKGLQITGNSIMPLLTYLGNPLTE
HFPTHAEEFNQNINGLSWGSANLAWRSVQLFQHYLSVASIFAVQAIDLRAGLEADHCDGRELLGETATELYETVYDLLERNCG
QESPFLFNDDEQSLEVDLQMLNGDLAGAGRMHEAVSSVTDSFLAEFCESGGGLEVLFQGPGGSSGSGHHHHHHHHH

Number of amino acids: 578

Molecular weight: 62683.55

Theoretical pI: 5.50

***Pb*PAL wt DNA sequence:**

ATGTTGGCGTCTTCGCCATCAGGACATACGAACCCTGTCTTGTCAGGGGCCCCCTTGAGTATTAACGTAGTTGCAG
ATATTGGACGCCAACGCCTGATTCCTTCATTAACCGATGACGAGCAGGTGTTGAATCGCGTTCATGCGTGCCGTGA
TGTAGTACAGAAGGCGGTCCGTAACAACGAACGTATTTATGGTATTACCACCGGCTTTGGGGGGATGAGCGACATC
CCAATTCCGCCGCAACATGTTGCACAAACGCAGGACAATTTATTGGCCTTTCTTTCGACCTCCACGGGAGCGAGCC
TGGACCCGCGTCATGTGCGTGCAGCTATGGCATTGCGCGCCAACGTCTTGCTGCAAGGGCGCTCAGGGGTCCGT
CTTGAACTTATTGAACGCTTGGTAGAATTCCTGCGCCAAGACGCGATCCCTGTCGTATGCGACCTGGGAAGCATCG
GCGCGAGTGGTGACTTGGTGCCACTGGGCGTAATCGCGCGTTCAATCATCGGGCACCCGTCGACAACGCAAGTG
AAGTACCAGGGTGAGCAAGCAGACTCTCACGACGTATTGCAGCAGCTGAATTACTCTGCCCTTCAGCTTGAAGCGA
AAGAAGGCCTTGCCTTAGTAAATGGCACCTCTTTCTCGAGCGCCATTGCGGCCAATTGTGTATTCGAGTCACAGCG
CCTGTTGTCATTATCACTGGTCCTGCAAAGTATCATGGTTCGTGCTCTGGGAGGCCATCCGGAGGCATTTCACCCA
TTTGTGGACGAAAACAAACCACACCCCGGCCAGGGTTGGAGTGCTCAGATGATGCGTGACCTGCTTTCGTACTCC
CCTAATGATAGCAAGCGTAACGGAGACTTAGCTCAAGACCGTTATTCCCTGCGCTGCTTGGCTCAATATTTCGCTC
CTATTGTTGAAGGAATTGCTCAGATTTCTCAGTCAATCAGCACGGAAATGAATGCGGTATCAGATAACCCACTTATT
GACGTAGATACCGGTCGTTTCCATCAGTCAGGAAATTTTTTAGGCCAATACGTGGCTATGTCGATGGACCAGCTGC
GTCGCCACTTAGGCCTTCTTGCGAAACATCTGGATGTTCAGATCGCTCAATTGGTGGCCCCGGCTTTCAATAATGG
ATTACCCGCATCCTTACGTGGAAACAGTTCACGTCCCTTTAACATGGGATTGAAGGGCTTACAGATCACAGGAAATT
CTATCATGCCCCTGCTGACCTACTTGGGAAACCCATTGACCGAACACTTTCCAACTCATGCAGAAGAGTTCAATCA
GAATATTAACGGTTTATCCTGGGGCAGTGCTAATTTAGCCTGGCGTAGCGTCCAACTTTTCCAGCACTACCTTAGC
GTGGCCAGTATCTTTGCGGTTCAGGCGATTGACTTGCGCGCGGGCTTGGAAGCAGATCACTGTGATGGTCGCGAG
CTTCTTGGGGAGACAGCCACTGAATTATATGAGACGGTGTATGACTTGCTGGAACGTAATTGCGGACAAGAGTCAC
CATTCTTGTTTAACGACGACGAACAGTCACTGGAGGTCGATCTTCAGATGTTGAATGGAGATTTAGCCGGTGCGGG
GCGTATGCACGAAGCGGTTAGCTCGGTGACGGATTCCTTTTTAGCAGAATTCTGTGAG

***La*AAL wt (His$_9$ tag):-**

MTITLDGASLTLADIDAVARGGAKVAITGDADVLARVHGSRDVIARAVERGEEIYGVTTLFGGMADVHVTRDQLIDVQKIALWQH
KSTTGPRLPDADVRAAMLLRANSLMRGASGVRIALIERLVAFLNAGASPQVYQRGSIGASGDLVPLTYIGASILGLSPEFLVDLD
GETLDCHTVLAKLGFTPMDPDPKEGLALNNGTGACTGVAANVMARALDAATMALGVHALFAQALLATDQSFDPYIHAQKPHP
GQVWSAARMAELLTGGRTIRSEAGGDRARRKGDLIQDRYGIRCLPQFFGPIVDGLSTAARQIETEANTANDNPLINPATGETFH
TGNFLAQYTAIAMDSTRYLIGLMCKHIDSQIALMITPAFSNGLTPALVGNMDTGVNVGLKSLHIGMNQMSTQISYLGQSVADRFP
THAEMYNQNINSQAMNAANLARDQMDVTEHFLAAALLTGVQAVEVRSRVETGSCDARDILSPATVPLYEAARVAAAGRPDKA
RTIVWDDMDGFLQPKVEGLLADIGSRGNVHAALQALRSSLDTFRASGGGLEVLFQGPGGSSGSGHHHHHHHHH

Number of amino acids: 576

Molecular weight: 61016.20

Theoretical pI: 5.97

***La*AAL wt DNA sequence:**

ATGGGATCATCACATCACCACCACCACCACTCCAGCGGTTTAGTTCCGCGTGGGTCTCACATGACGATTACCTTGG
ATGGCGCCTCCCTTACATTGGCGGATATCGATGCTGTCGCTCGTGGGGGAGCTAAAGTAGCTATCACCGGTGACG
CCGATGTCCTGGCACGTGTTCATGGGAGCCGTGATGTAATCGCTCGCGCTGTTGAGCGCGGTGAAGAGATCTACG
GCGTTACCACCTTGTTCGGCGGGATGGCCGACGTACACGTTACTCGTGACCAACTTATTGATGTACAGAAAATCGC
TTTATGGCAGCACAAAAGCACTACGGGACCTCGCCTGCCGGATGCAGATGTACGCGCTGCCATGCTTCTTCGCGC
TAACAGCTTGATGCGCGGTGCAAGTGGGGTGCGTATCGCATTAATCGAACGTCTTGTGGCATTTTTGAACGCAGGG
GCTAGTCCTCAGGTATACCAACGTGGATCTATTGGCGCTTCGGGCGACTTGGTTCCTCTGACTTATATTGGCGCGT
CGATCTTGGGATTGTCCCCAGAATTTTTAGTCGATTTAGATGGAGAGACACTTGATTGTCATACTGTCCTGGCGAAG
TTAGGCTTTACGCCAATGGATCCCGACCCAAAAGAAGGCCTTGCCCTTAATAATGGTACTGGCGCCTGTACGGGG
GTTGCAGCCAACGTCATGGCCCGCGCTTTGGACGCTGCGACTATGGCTTTGGGTGTACACGCCTTGTTTGCCCAG
GCTCTTTTGGCAACTGATCAGAGCTTTGATCCCTACATCCACGCACAAAAACCTCATCCAGGCCAAGTTTGGTCTG
CAGCTCGTATGGCAGAGCTGCTTACAGGAGGTCGTACAATTCGTTCAGAGGCAGGTGGTGACCGTGCACGCCGCA
AAGGTGATCTTATCCAGGACCGTTACGGCATTCGCTGTCTTCCTCAATTCTTCGGTCCGATCGTGGATGGGCTGAG
TACCGCTGCTCGTCAAATTGAGACCGAAGCAAACACCGCTAACGACAACCCGCTGATTAACCCGGCTACAGGGGA
GACATTTCATACTGGCAACTTTCTTGCCCAGTACACTGCGATTGCGATGGATTCAACACGCTACCTTATCGGTTTGA
TGTGCAAGCACATTGATAGCCAAATCGCTCTTATGATCACCCCTGCTTTTAGTAATGGTTTGACCCCAGCGTTGGTC
GGTAACATGGACACAGGTGTAAATGTTGGGTTGAAGAGCCTGCACATCGGCATGAACCAGATGTCAACGCAAATCT
CATATCTGGGGCAATCAGTTGCGGACCGTTTCCCAACACACGCAGAGATGTACAACCAGAACATTAATAGTCAAGC
TATGAATGCAGCGAACTTGGCTCGCGACCAGATGGACGTTACTGAACACTTTTTGGCAGCGGCCTTGTTGACTGGG
GTCCAGGCGGTTGAGGTGCGCTCGCGCGTTGAGACTGGAAGTTGCGACGCACGCGATATCTTGTCACCCGCGAC
TGTGCCTCTGTACGAAGCAGCGCGTGTGGCCGCCGCAGGGCGTCCGGACAAGGCCCGCACTATTGTATGGGATG
ACATGGATGGATTCCTGCAGCCGAAAGTAGAGGGGTTATTGGCAGACATTGGAAGTCGCGGGAATGTCCATGCGG
CGCTTCAAGCATTGCGCTCGTCACTTGATACTTTTCGTGCT

Strains and plasmids

E. coli BL21(DE3) was used as an expression host for protein production. The pET-29b(+)-PALs plasmids of both wild-type and studied variants (codon-optimized for *E. coli*) were obtained from Genscript Biotech (Netherlands) B.V. Kanamycin (Kan) was added where appropriate to a final concentration of 50 µg/mL. Agar plates supplemented with kanamycin were purchased from EPFL SV store.

Transformation of competent cells

For overexpression of wild types and PALs variants, competent *E. coli* BL21(DE3) cells (50 µL aliquot) were thawed on ice for 10 minutes and transformed with the appropriate pET-29b(+)-PAL (100 ng). The tubes were mixed by flicking, incubated on ice for 30 minutes, heat-shocked at 42 °C for 45 seconds and transferred back to ice for 1 minute. SOC medium (200 µL) was added to the tube under sterile conditions and incubated in an orbital shaker for 1 hour at 37 °C and 250 rpm. Then, 100 µL of the culture was spread across LB plates containing 50 µg/mL kanamycin. Plates were incubated overnight at 37 °C and stored at 4 °C.

Biocatalyst production

A single colony of *E. coli* BL21(DE3) carrying the pET-29b(+)-PALs plasmid was used to inoculate 50 mL of LB medium supplemented with 50 µg/mL kanamycin. Cells were grown overnight at 37 °C and 200 rpm. Then, 10 mL of starter culture was then used to inoculate 0.5 L of LB medium containing 50 µg/mL kanamycin. Cells were grown at 37 °C and 200 rpm up to an optical density (OD_{600}) of 0.6-0.8 at 600 nm. Protein expression was induced by addition of IPTG (0.5 mM) at 22 °C and 150 rpm overnight. Cells were harvested by centrifugation (5,000 rpm, 20 minutes, 4 °C), transferred in 50 mL falcon tubes and stored at –20 °C until further use.

General protocol for purification of wild-type PALs and *Pb*PAL variants

Cell pellets obtained from 0.5 L LB culture were resuspended in 20 mL of storage buffer (100 mM phosphate, 300 mM NaCl, pH 8.0) and lysed on ice *via* sonication. Cellular debris was removed by centrifugation at 9,000 rpm for 1 hour at 4 °C. The soluble extract was collected and loaded onto a HisPur^{TM} Cobalt-NTA-agarose column (4 mL of resin for 0.5 L LB culture) equilibrated with storage buffer (100 mM phosphate, 300 mM NaCl, pH 8.0). The column was washed with 20 mL wash buffer (25 mM imidazole, 100 mM phosphate, 300 mM NaCl, pH 8.1) to elute non-specific binding proteins. His_9-tagged PALs were eluted with 15 mL elution buffer (300 mM imidazole, 100 mM phosphate, 300 mM NaCl, pH 8.0). Fractions containing purified PALs were identified by SDS-PAGE, de-salted *via* dialysis (Spectra/Por 4 dialysis tubing, 12-14 kD MW cut off) into storage buffer (100 mM phosphate, 300 mM NaCl, pH 8.0) for 16 hours under stirring. The resulting proteins were stored at 4 °C until

further use. Protein concentration was determined in triplicate by absorbance measurements using the Thermo Scientific™ µDrop™ plate with Thermo Scientific™ Multiskan™ SkyHigh.

SDS-PAGE analysis

The samples (10 µL) were mixed with 6 µL of Laemmli buffer and heated to denature the proteins (95 °C, 5 min). The aliquots (12 µL) were loaded on a pre-cast Mini-PROTEAN TGX Stain-Free polyacrylamide gels (BIO-RAD). The electrophoresis was performed in a Mini-PROTEAN Tetra chamber (BIO-RAD) in running buffer (25 mM Tris HCl, 192 mM glycine, 0.1% w/v SDS) at 200 V for 30 min.

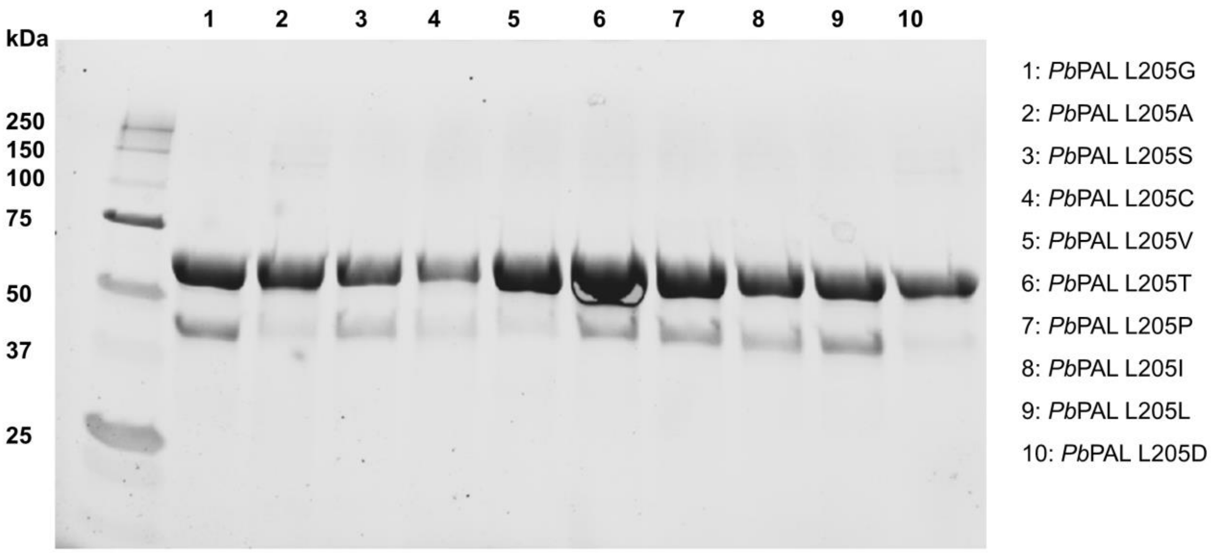

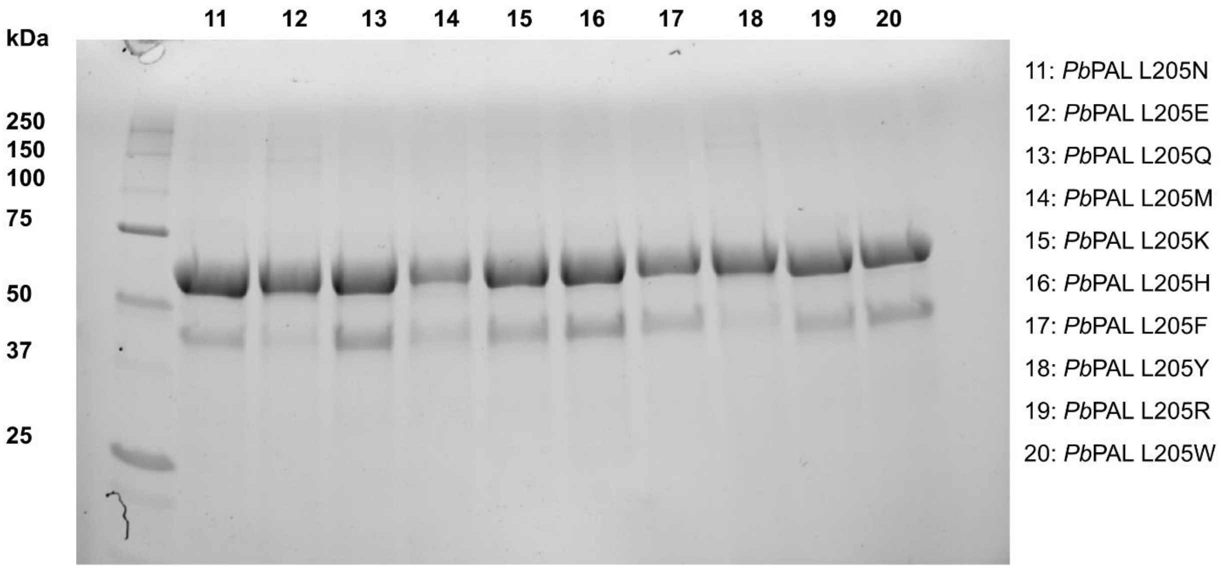

Figure S1. Representative SDS-PAGE analysis of purified L205X *Pb*PALs variants.

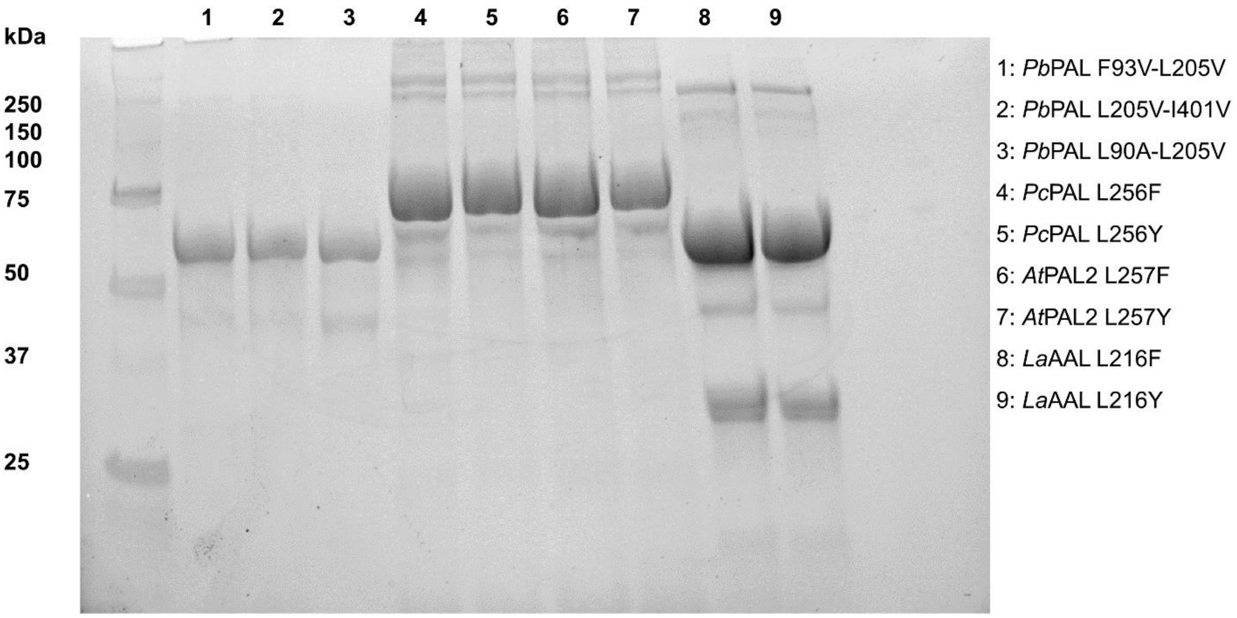

Figure S2. Representative SDS-PAGE analysis of *Pb*PAL variants F93V-L205F, L205F-I401V, L90A-L205V and *Pc*PAL L256F/Y, *At*PAL2 L257F/Y, *La*AAL L216F/Y.

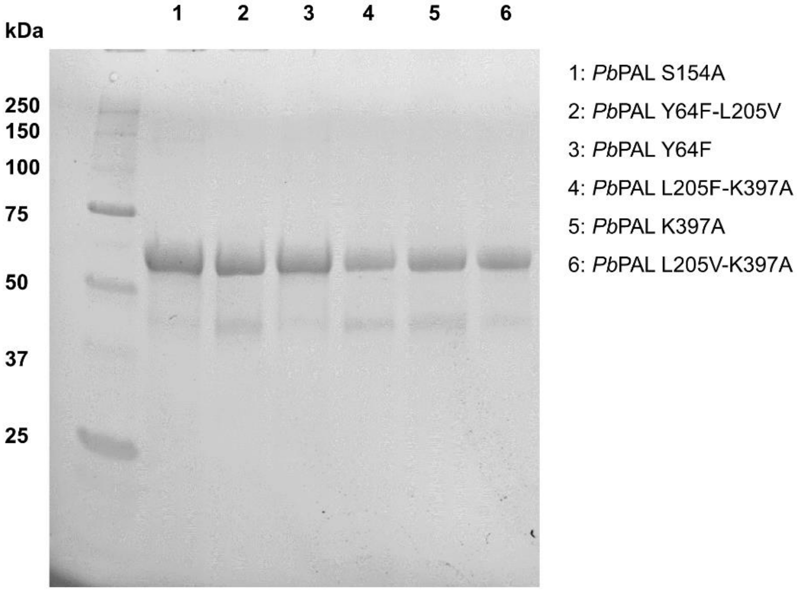

Figure S3. Representative SDS-PAGE analysis of *Pb*PAL variants employed in enantioselectivity mechanistic studies.

.

4. Enzyme kinetic characterization

Measurement of substrates **1a** and **1k** disappearances was carried out with wild-type *Pb*PAL and variants. Amination activity of PALs at different substrate concentrations was measured in triplicate in a UV-transparent 96-well plate with a continuous assay at 330 nm and 310 nm, respectively.

The kinetic parameters were obtained by hyperbolic fit using OriginPro 2020b. The velocity for each substrate concentration was measured in triplicate.

KINETIC DATA FOR 1a

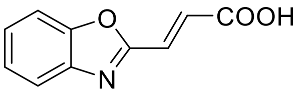

[PAL] = 0.8 µM
[**1a**] = 0.0125, 0.025, 0.04, 0.05, 0.10, 0.15, 0.20, 0.25, 0.30, 0.35, 0.40, 0.50, 1, 2, 5 mM.

Table S5. Kinetic constants for wild-type *Pb*PAL and *Pb*PAL L205F catalyzed amination reactions.

PAL	K_m (mM)	k_{cat} (s^{-1})	k_{cat}/K_m ($mM.s^{-1}$)	V_{max} ($mM.s^{-1}$)
*Pb*PAL WT	0.64 ± 0.05	68.9 ± 3.18	107.7	$5.51*10^{-5} \pm 2.54*10^{-6}$
*Pb*PAL L205F	0.37 ± 0.02	110.6 ± 3.91	298.9	$8.85*10^{-5} \pm 3.13*10^{-6}$

Measured at @330 nm for 30 min with 10 sec intervals. TRIPLICATE

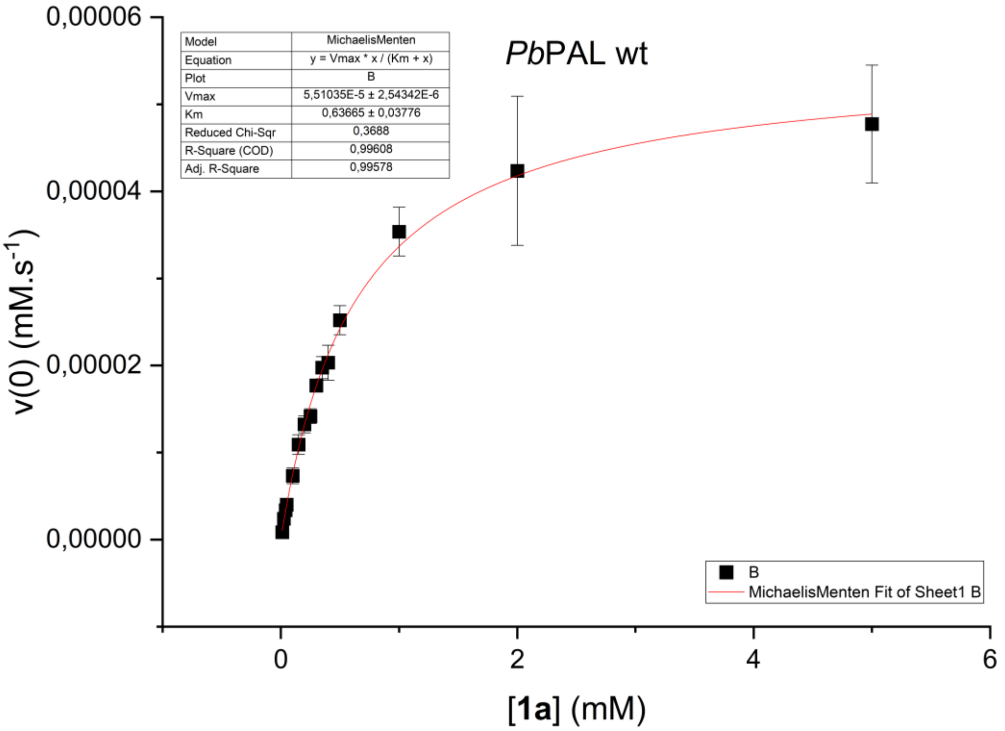

Figure S4. Michaelis-Menten curve for the ammonia addition reaction of **1a** catalyzed by wild-type *Pb*PAL.

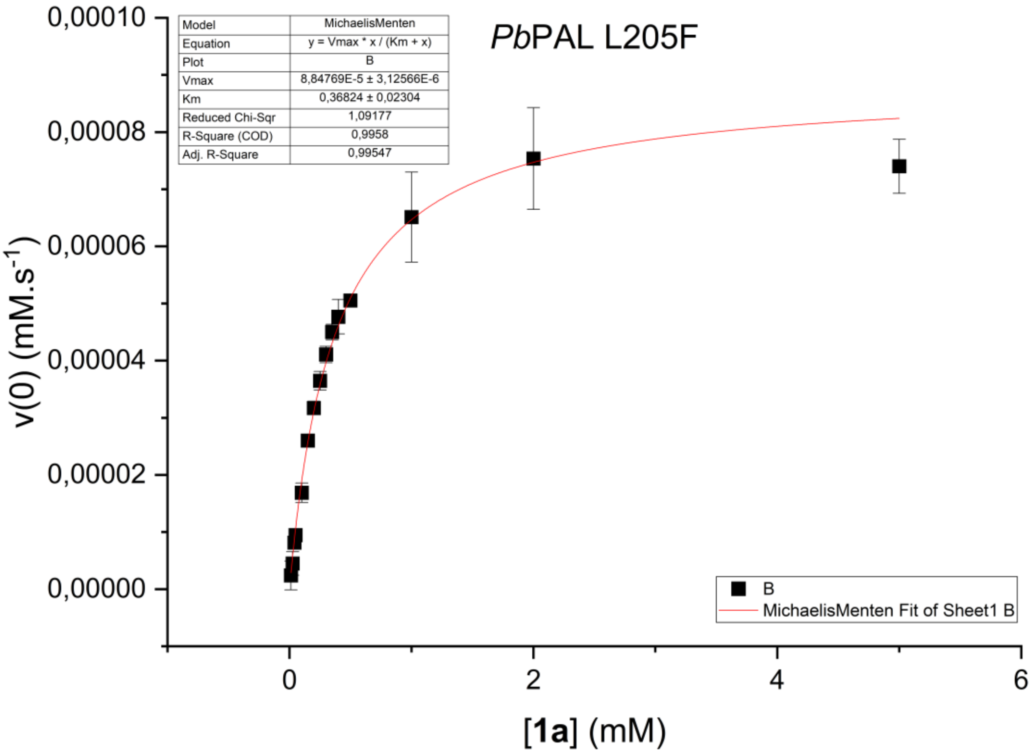

Figure S5. Michaelis-Menten curve for the ammonia addition reaction of **1a** catalyzed by *Pb*PAL L205F.

KINETIC DATA FOR 1k

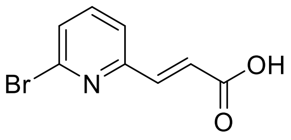

[PAL wt] = 12.0 µM, 30 min with 10 sec intervals, [**1k**] = 0.0125, 0.025, 0.04, 0.05, 0.10, 0.15, 0.20, 0.25, 0.30, 0.35, 0.40, 0.50, 1, 2, 5 mM.
[PAL L205F] = 15.0 µM, 20 min with 6 sec intervals, [**1k**] = 0.15, 0.25, 0.35, 0.45, 0.50, 0.55, 0.60, 1, 2, 5 mM.
[PAL K397A] = 15.8 µM, 10 min with 6 sec intervals, [**1k**] = 0.15, 0.25, 0.35, 0.40, 0.45, 0.50, 0.55, 0.60, 1, 2, 5 mM.
[PAL L205V-K397A] = 1.5 µM, 20 min with 6 sec intervals, [**1k**] = 0.15, 0.25, 0.35, 0.40, 0.45, 0.50, 0.55, 0.60, 1, 2, 5 mM.

Table S6. Kinetic constants for wild-type *Pb*PAL and *Pb*PAL L205F, *Pb*PAL K397A, *Pb*PAL L205V-K397A catalyzed aminations.

PAL	K_m (mM)	k_{cat} (s^{-1})	k_{cat}/K_m ($mM.s^{-1}$)	V_{max} ($mM.s^{-1}$)
*Pb*PAL wt	0.39 ± 0.02	9.17 ± 0.37	23.5	$1.10*10^{-4} \pm 4.48*10^{-6}$
*Pb*PAL L205F	0.87 ± 0.05	11.5 ± 0.38	13.2	$1.73*10^{-4} \pm 5.65*10^{-6}$
*Pb*PAL K397A	0.67 ± 0.02	75.9 ± 1.58	113.3	$0.0012 \pm 2.49*10^{-5}$
*Pb*PAL L205V-K397A	0.59 ± 0.03	216.7 ± 6.27	367.3	$3.25*10^{-4} \pm 9.41*10^{-6}$

Measured at @310 nm. TRIPLICATE

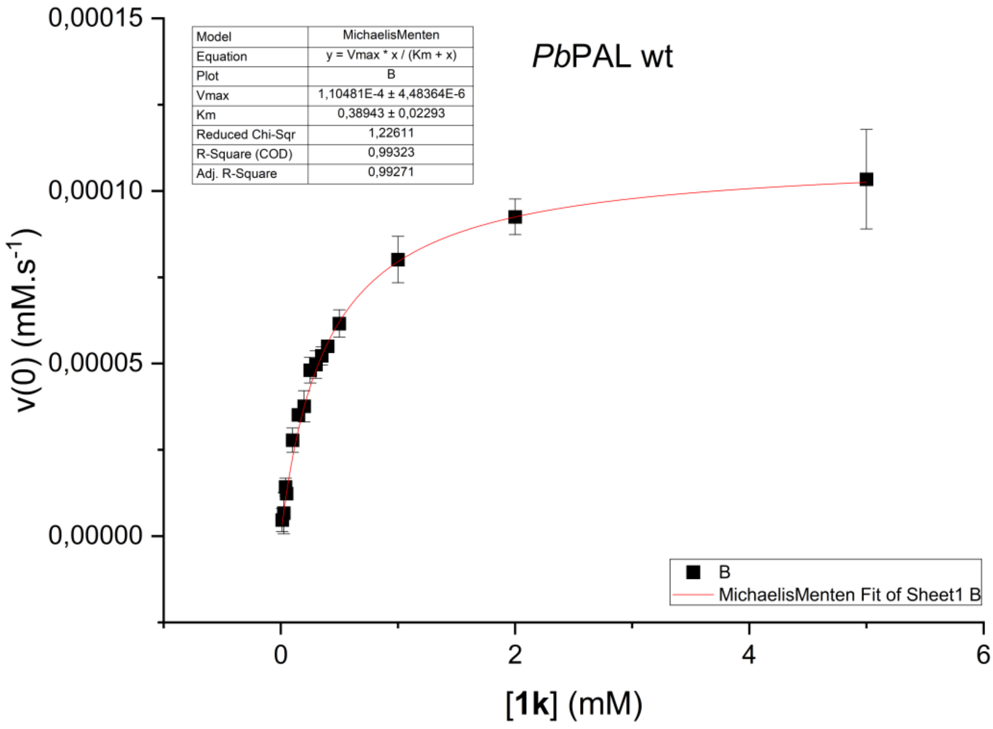

Figure S6. Michaelis-Menten curve for the ammonia addition reaction of **1k** catalyzed by wild-type *Pb*PAL.

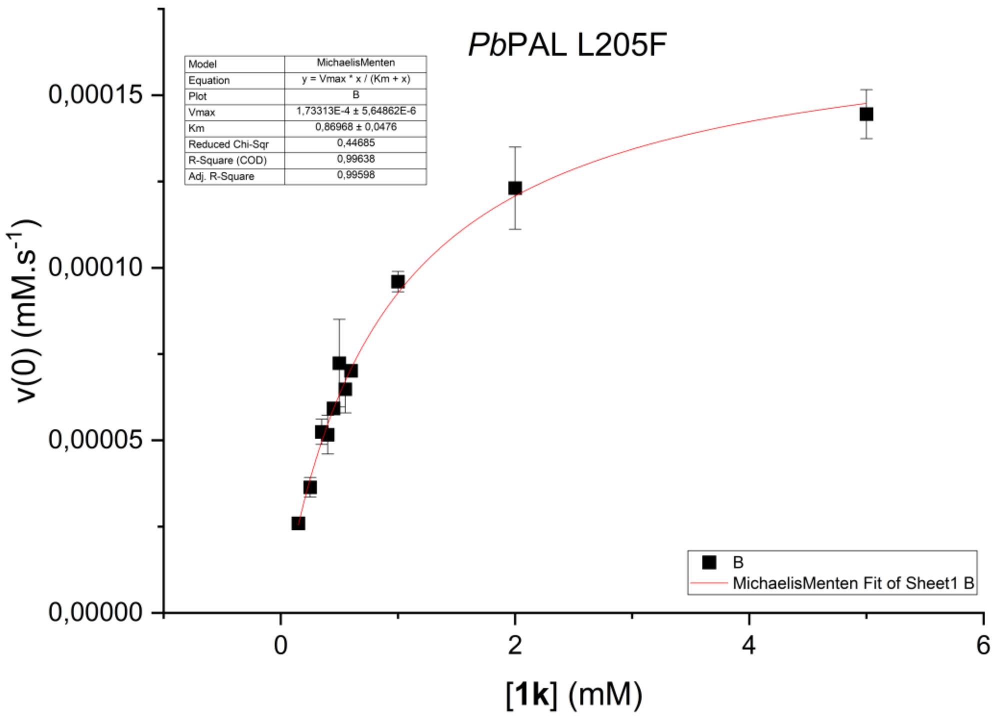

Figure S7. Michaelis-Menten curve for the ammonia addition reaction of **1k** catalyzed by *Pb*PAL L205F.

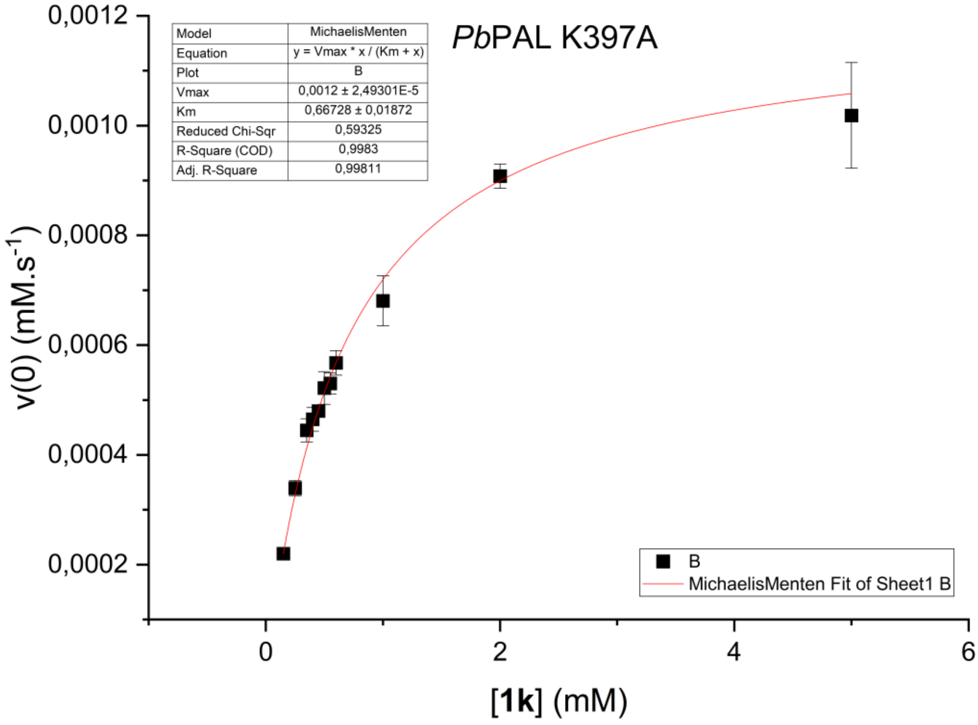

Figure S8. Michaelis-Menten curve for the ammonia addition reaction of **1k** catalyzed by *Pb*PAL K397A.

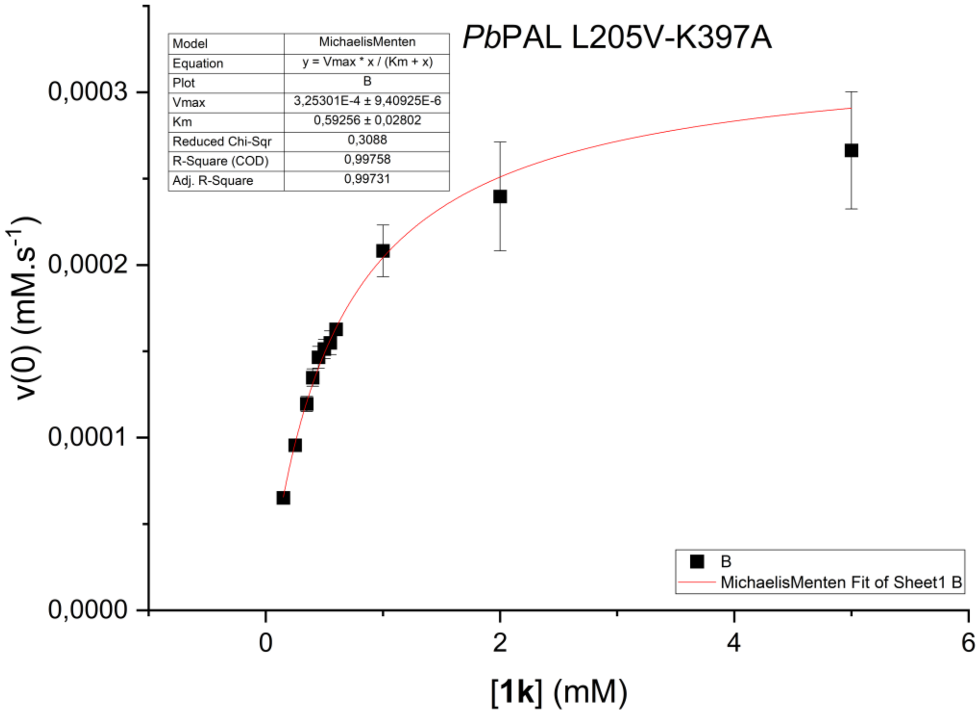

Figure S9. Michaelis-Menten curve for the ammonia addition reaction of **1k** catalyzed by *Pb*PAL L205V-K397A.

5. Synthesis of acrylic acids

General protocol for the synthesis of acrylic acids 1c-1f[1]

A 50 mL round-bottom flask equipped with magnetic stirrer and condenser was charged with a suitable aminophenol (615 mg, 5 mmol, 1.0 equiv.) and polyphosphoric acid (10 mL). Maleic anhydride (490 mg, 5 mmol, 1.0 equiv.) was added, and the reaction mixture was stirred at 140 °C overnight. The mixture was cooled to room temperature, the reaction mixture was quenched by the addition of crushed ice. The resulting suspension was extracted with EtOAc (3 × 20 mL), the combined organic phase dried over Na_2SO_4, filtered, and concentrated under reduced pressure. The crude product was purified by flash column chromatography (hexanes-EtOAc, 10:1 to 1:2) to afford the desired product.

General protocol for the synthesis of acrylic acids 1g-1i and 1k-1p[2,3]

A 20 mL vial equipped with a magnetic stir bar and a septum was charged with malonic acid (416 mg, 4 mmol, 2.0 equiv.), suitable aromatic aldehyde (2 mmol, 1.0 equiv.) in 3 mL of dry pyridine. The reaction was stirred at 110 °C overnight, cooled down to room temperature, and solvent removed under reduced pressure. Crude product was washed with water (5 mL), dried, and recrystallized from methanol to afford the desired product.

Protocol for the synthesis of 1j[4]

A 50 mL round-bottom flask equipped with magnetic stir bar and condenser was charged with malonic acid (1.45 g, 14 mmol, 2.0 equiv.), 5-(trifluoromethyl)picolinaldehyde (1.23 g, 7.0 mmol, 1.0 equiv.) in 10 mL of dry pyridine. The reaction was stirred at 110 °C overnight, cooled down to room temperature, and solvent removed under reduced pressure. Crude product was washed with water (2 × 5 mL), dried, and recrystallized from methanol to afford the desired product as white solid (1.25 g, 82%).

Table S7. Characterization data for compounds **1c-1p**.

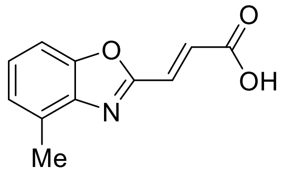	(*E*)-3-(4-methylbenzo[d]oxazol-2-yl)acrylic acid **1c**: brown solid (36%) **^{1}H NMR** (400 MHz, DMSO-*d$_6$*) δ 7.55 (d, *J* = 8.2 Hz, 1H), 7.46 – 7.32 (m, 2H), 7.23 (d, *J* = 7.5 Hz, 1H), 6.86 (d, *J* = 16.0 Hz, 1H), 2.55 (s, 3H). **^{13}C NMR** (101 MHz, DMSO-*d$_6$*) δ 166.0, 159.1, 149.8, 140.6, 130.7, 128.9, 128.3, 126.7, 125.5, 108.3, 16.1. **HRMS** (ESI/QTOF) m/z: $[M - H]^-$ Calcd for $C_{11}H_8NO_3^-$ 202.0510; Found 202.0509.
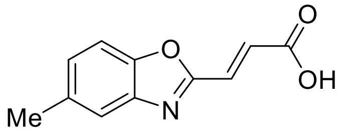	(*E*)-3-(5-methylbenzo[d]oxazol-2-yl)acrylic acid **1d**: brown solid (43%) **^{1}H NMR** (400 MHz, DMSO-*d$_6$*) δ 7.67 – 7.58 (m, 2H), 7.39 (d, *J* = 16.0 Hz, 1H), 7.30 (dd, *J* = 8.4, 1.2 Hz, 2H), 6.86 (d, *J* = 16.0 Hz, 1H), 2.43 (s, 3H). **^{13}C NMR** (101 MHz, DMSO-*d$_6$*) δ 166.0, 159.9, 148.3, 141.5, 134.8, 129.0, 128.3, 128.0, 120.1, 110.6, 20.9. **HRMS** (ESI/QTOF) m/z: $[M - H]^-$ Calcd for $C_{11}H_8NO_3^-$ 202.0510; Found 202.0510.
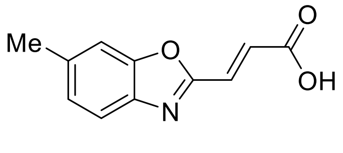	(*E*)-3-(6-methylbenzo[d]oxazol-2-yl)acrylic acid **1e**: brown solid, (55%) **^{1}H NMR** (400 MHz, DMSO-*d$_6$*) δ 7.68 (d, *J* = 8.2 Hz, 1H), 7.57 (s, 1H), 7.38 (d, *J* = 16.0 Hz, 1H), 7.25 (d, *J* = 8.2 Hz, 1H), 6.84 (d, *J* = 16.0 Hz, 1H), 2.46 (s, 4H). **^{13}C NMR** (101 MHz, DMSO-*d$_6$*) δ 166.1, 159.3, 150.3, 139.2, 137.3, 128.8, 128.2, 126.5, 119.9, 111.0, 21.4. **HRMS** (ESI/QTOF) m/z: $[M - H]^-$ Calcd for $C_{11}H_8NO_3^-$ 202.0510; Found 202.0510.
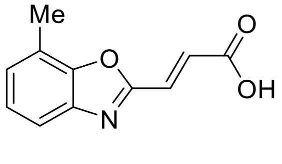	(*E*)-3-(7-methylbenzo[d]oxazol-2-yl)acrylic acid **1f**: grey solid (22%) **^{1}H NMR** (400 MHz, DMSO-*d$_6$*) δ 7.63 (dd, *J* = 7.0, 1.9 Hz, 2H), 7.42 (d, *J* = 16.0 Hz, 1H), 7.32 (d, *J* = 7.0 Hz, 2H), 6.92 (d, *J* = 16.0 Hz, 1H), 2.52 (s, 4H). **^{13}C NMR** (101 MHz, DMSO-*d$_6$*) δ 166.7, 166.0, 159.6, 149.2, 141.0, 130.2, 129.2, 128.3, 127.6, 125.2, 121.2, 117.8, 14.7. **HRMS** (ESI/QTOF) m/z: $[M - H]^-$ Calcd for $C_{11}H_8NO_3^-$ 202.0510; Found 202.0508.
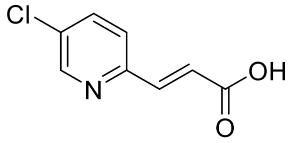	(*E*)-3-(5-chloropyridin-2-yl)acrylic acid **1g**: white solid (79%) **^{1}H NMR** (400 MHz, D_2O) δ 8.34 (d, *J* = 2.3 Hz, 1H), 7.74 (dd, *J* = 8.5, 2.4 Hz, 1H), 7.45 (d, *J* = 8.5 Hz, 1H), 7.16 (d, *J* = 16.0 Hz, 1H), 6.59 (d, *J* = 16.0 Hz, 1H). **^{13}C NMR** (101 MHz, D_2O) δ 175.5, 152.5, 148.7, 138.7, 132.7, 130.1, 125.1. **HRMS** (ESI/QTOF) m/z: $[M - H]^-$ Calcd for $C_8H_5ClNO_2^-$ 182.0009; Found 182.0011.

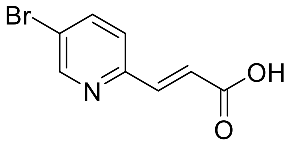	(*E*)-3-(5-bromopyridin-2-yl)acrylic acid **1h**: white solid (88%)<br>**^{1}H** NMR (400 MHz, D_2O) δ 8.49 (d, *J* = 2.2 Hz, 1H), 7.93 (dd, *J* = 8.4, 2.3 Hz, 1H), 7.45 (d, *J* = 8.5 Hz, 1H), 7.19 (d, *J* = 16.0 Hz, 1H), 6.66 (d, *J* = 16.0 Hz, 1H). **^{13}C NMR** (101 MHz, D_2O) δ 175.6, 152.9, 151.0, 141.5, 138.6, 130.2, 125.5, 121.4. **HRMS** (ESI/QTOF) m/z: [M - H]$^-$ Calcd for $C_8H_5BrNO_2^-$ 225.9509; Found 225.9510.
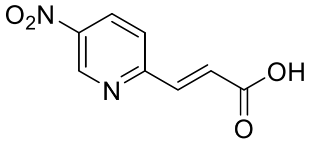	(*E*)-3-(5-nitropyridin-2-yl)acrylic acid **1i**: brown solid (52%)<br>**^{1}H** NMR (400 MHz, D_2O) δ 9.23 (s, 1H), 8.54 (s, 1H), 7.74 (s, 1H), 7.23 (d, *J* = 14.8 Hz, 1H), 6.76 (d, *J* = 14.5 Hz, 1H). **^{13}C** NMR (101 MHz, D_2O) δ 174.6, 160.0, 145.7, 144.4, 137.2, 133.9, 124.5. **HRMS** (ESI/QTOF) m/z: [M - H]$^-$ Calcd for $C_8H_5N_2O_4^-$ 193.0255; Found 193.0257.
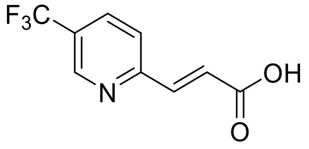	(*E*)-3-(5-(trifluoromethyl)pyridin-2-yl)acrylic acid **1j**: white solid (82%)<br>**^{1}H NMR** (400 MHz, DMSO-*d$_6$*) δ 13.71 – 12.40 (br, 1H), 9.00 (s, 1H), 8.28 (dd, *J* = 8.2, 2.2 Hz, 1H), 7.96 (d, *J* = 8.2 Hz, 1H), 7.67 (d, *J* = 15.7 Hz, 1H), 6.95 (d, *J* = 15.7 Hz, 1H). **^{13}C NMR** (101 MHz, DMSO-*d$_6$*) δ 166.9, 156.2, 146.6 (q, *J* = 4.0 Hz), 141.4, 134.8 (q, *J* = 4.0 Hz), 125.5, 125.2 (q, *J* = 33.0 Hz), 124.3, 123.6 (q, *J* = 273 Hz). **HRMS** (ESI/QTOF) m/z: [M - H]$^-$ Calcd for $C_9H_5F_3NO_2^-$ 216.0278; Found 216.0279.
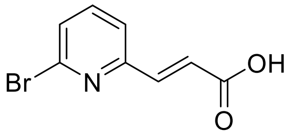	(*E*)-3-(6-bromopyridin-2-yl)acrylic acid **1k**: beige solid (74%)<br>**^{1}H NMR** (400 MHz, D_2O) δ 8.47 (d, *J* = 2.0 Hz, 1H), 7.90 (dd, *J* = 8.4, 2.2 Hz, 1H), 7.43 (d, *J* = 8.5 Hz, 1H), 7.17 (d, *J* = 16.0 Hz, 1H), 6.64 (d, *J* = 16.0 Hz, 1H). **^{13}C NMR** (101 MHz, D_2O) δ 175.6, 152.9, 151.0, 141.4, 138.6, 130.2, 125.5, 121.4. **HRMS** (ESI/QTOF) m/z: [M - H]$^-$ Calcd for $C_8H_5BrNO_2^-$ 225.9509; Found 225.9510.
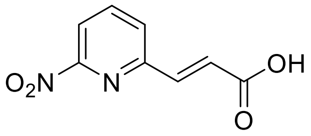	(*E*)-3-(6-nitropyridin-2-yl)acrylic acid **1l**: beige solid (35%)<br>**^{1}H NMR** (400 MHz, D_2O) δ 9.29 (d, *J* = 2.3 Hz, 1H), 8.58 (dd, *J* = 8.7, 2.5 Hz, 1H), 7.80 (d, *J* = 8.7 Hz, 1H), 7.31 (d, *J* = 16.0 Hz, 1H), 6.84 (d, *J* = 16.0 Hz, 1H). **^{13}C NMR** (101 MHz, DMSO-*d$_6$*) δ 166.7, 157.5, 145.1, 143.9, 140.7, 132.7, 126.8, 124.8. **HRMS** (ESI/QTOF) m/z: [M - H]$^-$ Calcd for $C_8H_5N_2O_4^-$ 193.0255; Found 193.0256.
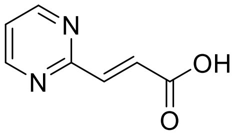	(*E*)-3-(pyrimidin-2-yl)acrylic acid **1m**: beige solid (90%)<br>**^{1}H NMR** (400 MHz, DMSO-*d$_6$*) δ 8.88 (d, *J* = 4.9 Hz, 2H), 7.51 – 7.39 (m, 2H), 7.00 (d, *J* = 15.7 Hz, 1H). **^{13}C NMR** (101 MHz, DMSO-*d$_6$*) δ 166.8, 161.8, 157.7, 142.1, 127.8, 121.0. **HRMS** (ESI/QTOF) m/z: [M - H]$^-$ Calcd for $C_7H_5N_2O_2^-$ 149.0351; Found 149.0355.
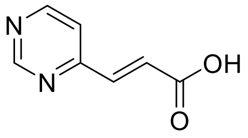	(*E*)-3-(pyrimidin-4-yl)acrylic acid **1n**: beige solid (83%)<br>**^{1}H NMR** (400 MHz, DMSO-*d$_6$*) δ 9.26 – 9.19 (m, 1H), 8.89 (d, *J* = 5.1 Hz, 1H), 7.82 (dd, *J* = 5.1, 1.2 Hz, 2H), 7.53 (d, *J* = 15.8 Hz, 1H), 7.03 (d, *J* = 15.8 Hz, 1H). **^{13}C NMR** (101 MHz, DMSO-*d$_6$*) δ 166.7, 159.2, 158.7, 158.7, 140.7, 127.1, 120.8. **HRMS** (ESI/QTOF) m/z: [M - H]$^-$ Calcd for $C_7H_5N_2O_2^-$ 149.0351; Found 149.0353.
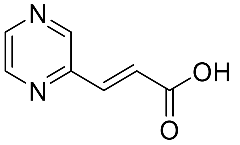	(*E*)-3-(pyrazin-2-yl)acrylic acid **1o**: beige solid (75%)<br>**^{1}H NMR** (400 MHz, DMSO-*d$_6$*) δ 8.95 (d, *J* = 1.3 Hz, 1H), 8.73 – 8.67 (m, 1H), 8.64 (d, *J* = 2.4 Hz, 1H), 6.92 (d, *J* = 15.8 Hz, 1H). **^{13}C NMR** (101 MHz, DMSO-*d$_6$*) δ 166.9, 148.0, 145.5, 145.3, 145.0, 139.6, 124.8. **HRMS** (ESI/QTOF) m/z: [M - H]$^-$ Calcd for $C_7H_5N_2O_2^-$ 149.0351; Found 149.0353.
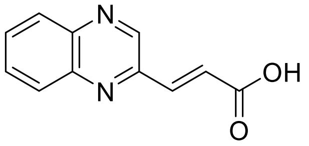	(E)-3-(quinoxalin-2-yl)acrylic acid **1p**: brown solid (30%)<br>**^{1}H NMR** (400 MHz, DMSO-*d$_6$*) δ 9.35 (s, 1H), 8.12 (dt, *J* = 7.9, 4.1 Hz, 3H), 7.95 – 7.85 (m, 2H), 7.80 (d, *J* = 16.0 Hz, 1H), 7.16 (d, *J* = 16.0 Hz, 1H). **^{13}C NMR** (101 MHz, DMSO-*d$_6$*) δ 167.3, 148.7, 146.2, 142.3, 142.0, 140.7, 131.4, 131.4, 129.9, 129.4, 126.8. **HRMS** (ESI/QTOF) m/z: [M - H]$^-$ Calcd for $C_{11}H_7N_2O_2^-$ 199.0508; Found 199.0511.

6. Analytical scale hydroaminations

Hydroamination of 1a at 2.5 mM substrate concentration with L205X *Pb*PAL variants

Substrate **1a** (20 µL, 25 mM stock solution in DMSO, final concentration 2.5 mM) was added to a solution of ammonium carbonate (pH 9.5, 4.6 M, 130 µL) in a 1.5 mL Eppendorf tube. PALs (50 µL, final concentration 3 µM) were added to the mixture. The resulting solutions were incubated in a thermomixer (300 rpm, 30 °C) for 24 h. An aliquot (100 µL) was diluted with MeOH (100 µL) and centrifuged (8000 rpm, 5 min, 4 °C). The supernatant was used directly for HPLC analysis. Conversions determined using Method A, enantiomeric excess of D-enantiomer determined using Method B.

Table S8. Hydroamination of **1a** at 2.5 mM substrate concentration with *Pb*PAL L205X variants.

L205X	Conversion, %	*ee*, %[a]
**G**	18	-33
**A**	34	-3
**S**	99	-36
**C**	99	30
**V**	97	-6
**T**	95	9
**P**	84	-35
**I**	97	27
**L**	99	-10
**D**	16	-3
**N**	99	79
**E**	34	33
**Q**	93	-20
**M**	100	65
**K**	59	57
**H**	94	55
**F**	98	99
**Y**	79	90
**R**	6	-45
**W**	13	95

[a]*ee* of the D-enantiomer

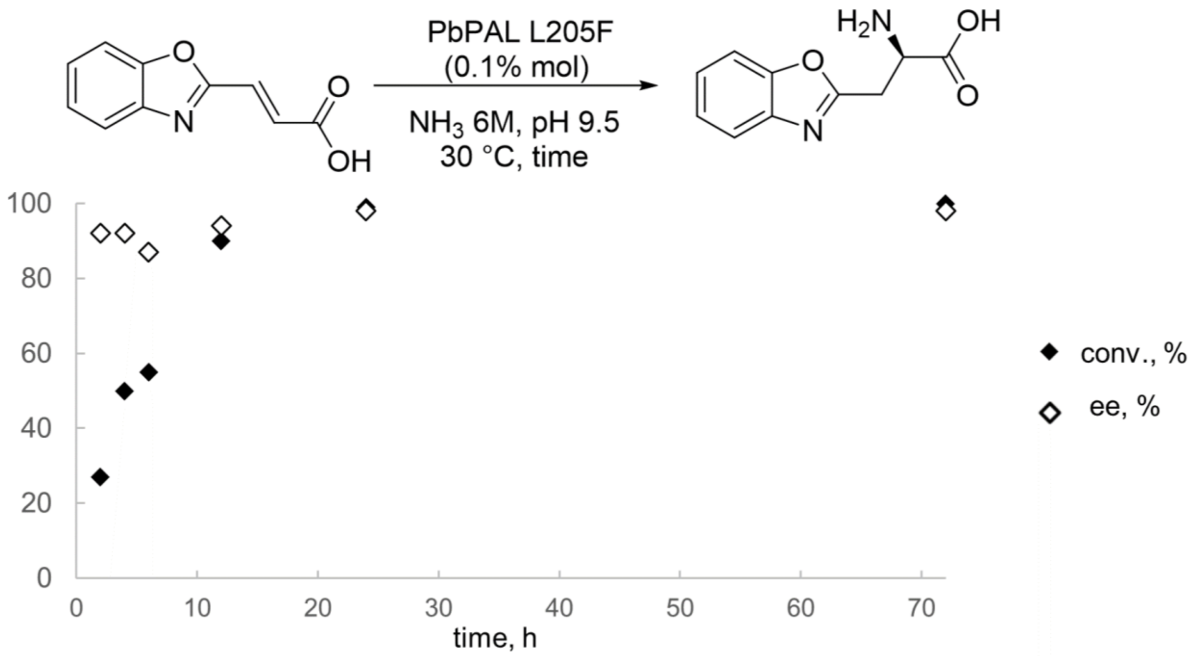

Figure S10. Time course of conversion and enantioselectivity in the *Pb*PAL L205F-catalyzed hydroamination of **1a**.

Hydroamination of 1a at 2.5 mM substrate concentration with *Pb*PAL L205F/Y and corresponding *Pc*PAL, *At*PAL2, and *La*AAL homologs

Substrate **1a** (20 µL, 25 mM stock solution in DMSO, final concentration 2.5 mM) was added to a solution of ammonium carbonate (pH 9.5, 4.6 M, 130 µL) in a 1.5 mL Eppendorf tube. PALs (50 µL, final concentration 3 µM) were added to the mixture. The resulting solutions were incubated in a thermomixer (300 rpm, 30 °C) for 24 h. An aliquot (100 µL) was diluted with MeOH (100 µL) and centrifuged (8000 rpm, 5 min, 4 °C). The supernatant was used directly for HPLC analysis. Conversions determined using Method A, enantiomeric excess of D-enantiomer determined using Method B.

Table S9. Hydroamination of **1a** at 2.5 mM substrate concentration with PAL variants.

PAL	Conversion, %	*ee*, %[a]
*Pb*PAL L205F	98	99
*Pb*PAL L205Y	79	90
*La*AAL L216F	18	90
*La*AAL L216Y	15	93
*Pc*PAL L256F	10	82
*Pc*PAL L256Y	29	85
*At*PAL2 L257F	50	77
*At*PAL2 L257Y	35	81

[a]*ee* of the D-enantiomer

Hydroamination of 1b at 2.5 mM substrate concentration with *Pb*PAL L205X variants

Substrate **1b** (20 µL, 25 mM stock solution in DMSO, final concentration 2.5 mM) was added to a solution of ammonium carbonate (pH 9.5, 4.6 M, 130 µL) in a 1.5 mL Eppendorf tube. PALs (50 µL, final concentration 3 µM) were added to the mixture. The resulting solutions were incubated in a thermomixer (300 rpm, 30 °C) for 24 h. An aliquot (100 µL) was diluted with MeOH (100 µL) and centrifuged (8000 rpm, 5 min, 4 °C). The supernatant was used directly for HPLC analysis. Conversions determined using Method A, enantiomeric excess of D-enantiomer determined using Method C.

Table S10. Hydroamination of **1b** at 2.5 mM substrate concentration with *Pb*PAL L205X variants.

L205X	Conversion, %	*ee*, %[a]
**I**	95	-81
**L**	92	-79
**M**	96	-73
**K**	45	-91
**H**	96	-63
**F**	56	25
**Y**	48	94
**W**	9	81

[a]*ee* of the D-enantiomer

Hydroamination of 1c-1f at 2.5 mM substrate concentration with *Pb*PAL variants

Substrates **1c-1f** (20 μL, 25 mM stock solution in DMSO, final concentration 2.5 mM) was added to a solution of ammonium carbonate (pH 9.5, 4.6 M, 130 μL) in a 1.5 mL Eppendorf tube. PALs (50 μL, final concentration 3 μM) were added to the mixture. The resulting solutions were incubated in a thermomixer (300 rpm, 30 °C) for 24 h. An aliquot (100 μL) was diluted with MeOH (100 μL) and centrifuged (8000 rpm, 5 min, 4 °C). The supernatant was used directly for HPLC analysis. Conversions determined using Method A, enantiomeric excess of D-enantiomers determined using Method B.

Table S11. Hydroamination of **1c-1f** at 2.5 mM substrate concentration with *Pb*PAL WT and variants.

Substrate	*Pb*PAL WT		*Pb*PAL L205F		*Pb*PAL F93V-L205F		*Pb*PAL L205F-I401V	
	Conv., %	*ee*, %[a]	Conv., %	*ee*, %[a]	Conv., %	*ee*, %[a]	Conv., %	*ee*, %[a]
**1c**	99	-63	45	83	97	50	83	70
**1d**	99	-50	36	90	99	87	62	89
**1e**	96	-59	71	79	>99	17	51	87
**1f**	99	-30	99	96	>99	68	99	91

[a]*ee* of the D-enantiomer

Hydroamination of 1k at 2.5 mM substrate concentration with PAL variants

Substrate **1k** (20 μL, 25 mM stock solution in DMSO, final concentration 2.5 mM) was added to a solution of ammonium carbonate (pH 9.5, 4.6 M, 130 μL) in a 1.5 mL Eppendorf tube. PALs (50 μL, final concentration 3 μM) were added to the mixture. The resulting solutions were incubated in a thermomixer (300 rpm, 30 °C) for 24 h. An aliquot (100 μL) was diluted with MeOH (100 μL) and centrifuged (8000 rpm, 5 min, 4 °C). The supernatant was used directly for HPLC analysis. Conversions determined using Method A, enantiomeric excess of D-enantiomers determined using Method D.

Table S12. Hydroamination of **1k** at 2.5 mM substrate concentration with PAL variants.

PAL	conv., %	*ee*, %[a]
*Pb*PAL WT	>99	-59
*Pb*PAL L205F	43	95
*Pb*PAL L205Y	2	n.a.
*Pc*PAL L256F	1	n.a.
*Pc*PAL L256Y	1	n.a.
*At*PAL2 L257F	8	75
*At*PAL2 L257Y	1	n.a.
*La*AAL L216F	0	n.a.

*La*AAL L216Y	1	n.a.
*Pb*PAL L205F-F93V	95	75
*Pb*PAL L205F-I401V	82	65

[a]*ee* of the D-enantiomer

Determination of enantioselectivity of 1m-1o hydroamination at 2.5 mM substrate concentration with *Pb*PAL variants

Substrates **1m-1o** (20 µL, 25 mM stock solution in DMSO, final concentration 2.5 mM) was added to a solution of ammonium carbonate (pH 9.5, 4.6 M, 130 µL) in a 1.5 mL Eppendorf tube. PALs (50 µL, final concentration 3 µM) were added to the mixture. The resulting solutions were incubated in a thermomixer (300 rpm, 30 °C) for 24 h. An aliquot (100 µL) was diluted with MeOH (100 µL) and centrifuged (8000 rpm, 5 min, 4 °C). Enantiomeric excess of D-enantiomers determined using FDVA-derivatization and Method A.

Table S13. Enantiselectivity of hydroamination of **1m-1o** at 2.5 mM substrate concentration with *Pb*PAL WT and variants.

**Substrate**	***Pb*PAL WT**	***Pb*PAL L205F**	***Pb*PAL F93-L205F**	***Pb*PAL L205F-I401V**
	ee, %[a]			
**1m**	-53	85	*n.a.*	70
**1n**	-50	89	*n.a.*	87
**1o**	-33	78	86	77

[a]*ee* of the D-enantiomer

Hydroamination of 1p at 2.5 mM substrate concentration with PAL variants

Substrate **1p** (20 µL, 25 mM stock solution in DMSO, final concentration 2.5 mM) was added to a solution of ammonium carbonate (pH 9.5, 4.6 M, 130 µL) in a 1.5 mL Eppendorf tube. PALs (50 µL, final concentration 3 µM) were added to the mixture. The resulting solutions were incubated in a thermomixer (300 rpm, 30 °C) for 24 h. An aliquot (100 µL) was diluted with MeOH (100 µL) and centrifuged (8000 rpm, 5 min, 4 °C). The supernatant was used directly for HPLC analysis. Conversions determined using Method A.

Table S14. Hydroamination of **1p** at 2.5 mM substrate concentration with *Pb*PAL WT and variants.

**PAL**	**conv., %**
*Pb*PAL WT	83
*Pb*PAL L205F	45
*Pb*PAL L205Y	20
*Pb*PAL F93-L205F	38
*Pb*PAL L205F-[illegible]	10
*Pb*PAL L90A-L205F	87

Hydroamination of (*E*)-3-(pyridin-2-yl)acrylic acid at 2.5 mM with *Pb*PAL L205F/Y and corresponding *Pc*PAL, *At*PAL2, and *La*AAL homologs

(*E*)-3-(pyridin-2-yl)acrylic acid (20 µL, 25 mM stock solution in DMSO, final concentration 2.5 mM) was added to a solution of ammonium carbonate (pH 9.5, 4.6 M, 130 µL) in a 1.5 mL Eppendorf tube. PALs (50 µL, final concentration 3 µM) were added to the mixture. The resulting solutions were incubated in a thermomixer (300 rpm, 30 °C) for 24 h. An aliquot (100 µL) was diluted with MeOH (100 µL) and centrifuged (8000 rpm, 5 min, 4 °C). Conversions determined using Method A (without correction for relative response factor at 210 nm), enantiomeric excess of D-enantiomer determined using FDVA-derivatization and HPLC Method A.

Table S15. Hydroamination of (*E*)-3-(pyridin-2-yl)acrylic acid at 2.5 mM substrate concentration with with *Pb*PAL L205F/Y and corresponding *Pc*PAL, *At*PAL2, and *La*AAL homologs.

**PAL**	**conv., %**	***ee*, %[a]**
*Pb*PAL WT	52	-87
*Pb*PAL L205F	27	67
*Pb*PAL L205Y	8	95
*Pc*PAL L256F	8	-27
*Pc*PAL L256Y	6	75
*At*PAL2 L257F	21	-32
*At*PAL2 L257Y	5	94
*La*AAL L216F	6	-30
*La*AAL L216Y	<1	*n.a.*

[a]*ee* of the D-enantiomer

Hydroamination of (*E*)-3-(2-bromopyridin-4-yl)acrylic acid at 2.5 mM concentration with PAL variants

(*E*)-3-(2-bromopyridin-4-yl)acrylic acid (20 µL, 25 mM stock solution in DMSO, final concentration 2.5 mM) was added to a solution of ammonium carbonate (pH 9.5, 4.6 M, 130 µL) in a 1.5 mL Eppendorf tube. PALs (50 µL, final concentration 3 µM) were added to the mixture. The resulting solutions were incubated in a thermomixer (300 rpm, 30 °C) for 24 h. An aliquot (100 µL) was diluted with MeOH (100 µL) and centrifuged (8000 rpm, 5 min, 4 °C). Conversions determined using Method A (without correction for relative response factor at 210 nm), enantiomeric excess of D-enantiomers determined using Method D (the peak with the shorter t_r was assigned to the D-enantiomer, consistent with other substrates).

Table S16. Hydroamination of (*E*)-3-(2-bromopyridin-4-yl)acrylic acid at 2.5 mM substrate concentration with PAL variants.

**PAL**	**conv., %**	***ee*, %[a]**
*Pb*PAL WT	80	-67
*Pb*PAL L205F	92	-3
*Pb*PAL L205Y	3	*n.a.*
*Pc*PAL L256F	1	*n.a.*
*Pc*PAL L256Y	0	*n.a.*
*At*PAL2 L257F	77	-76
*At*PAL2 L257Y	1	*n.a.*
*La*AAL L216F	0	*n.a.*
*La*AAL L216Y	0	*n.a.*

[a]*ee* of the D-enantiomer

Hydroamination of 1a at 2.5 mM substrate concentration with *Pb*PAL S154A, *Pb*PAL Y64F, and *Pb*PAL Y64F-L205F variants

Substrate **1a** (20 µL, 25 mM stock solution in DMSO, final concentration 2.5 mM) was added to a solution of ammonium carbonate (pH 9.5, 4.6 M, 130 µL) in a 1.5 mL Eppendorf tube. PALs (50 µL, final concentration 3 µM) were added to the mixture. The resulting solutions were incubated in a thermomixer (300 rpm, 30 °C) for 24 h. An aliquot (100 µL) was diluted with MeOH (100 µL) and centrifuged (8000 rpm, 5 min, 4 °C). The supernatant was used directly for HPLC analysis. Conversions determined using Method A, enantiomeric excess of D-enantiomer determined using Method B.

Table S17. Hydroamination of **1a** acid at 2.5 mM substrate concentration with PAL variants.

**PAL**	**conv., %**	***ee*, %[a]**
*Pb*PAL WT	>99	-7
*Pb*PAL S154A	3	*n.a.*
*Pb*PAL L205F	99	96
*Pb*PAL Y64F	32	45
*Pb*PAL Y64F-L205F	19	95

[a]*ee* of the D-enantiomer

Hydroamination of 1k at 2.5 mM substrate concentration with *Pb*PAL S154A, *Pb*PAL Y64F, and *Pb*PAL Y64F-L205F variants

Substrate **1k** (20 µL, 25 mM stock solution in DMSO, final concentration 2.5 mM) was added to a solution of ammonium carbonate (pH 9.5, 4.6 M, 130 µL) in a 1.5 mL Eppendorf tube. PALs (50 µL, final concentration 3 µM) were added to the mixture. The resulting solutions were incubated in a thermomixer (300 rpm, 30 °C) for 24 h. An aliquot (100 µL) was diluted with MeOH (100 µL) and centrifuged (8000 rpm, 5 min, 4 °C). The supernatant was used directly for HPLC analysis. Conversions determined using Method A, enantiomeric excess of D-enantiomer determined using Method D.

Table S18. Hydroamination of **1k** acid at 2.5 mM substrate concentration with PAL variants.

**PAL**	**conv., %**	***ee*, %[a]**
*Pb*PAL WT	92	-84
*Pb*PAL S154A	0	*n.a.*
*Pb*PAL L205F	89	94
*Pb*PAL Y64F	0	*n.a.*
*Pb*PAL Y64F-[illegible]	1	*n.a.*

[a]*ee* of the D-enantiomer

Hydroamination of 1a-1b and 1h-1l at 2.5 mM substrate concentration with *Pb*PAL L205F-K397A, *Pb*PAL K397A, and *Pb*PAL Y64F-L205F variants

Substrates **1a-1b** and **1h-1l** (20 µL, 25 mM stock solution in DMSO, final concentration 2.5 mM) were added to a solution of ammonium carbonate (pH 9.5, 4.6 M, 130 µL) in a 1.5 mL Eppendorf tube. PALs (50 µL, final concentration 3 µM) were added to the mixture. The resulting solutions were incubated in a thermomixer (300 rpm, 30 °C) for 24 h. An aliquot (100 µL) was diluted with MeOH (100 µL) and centrifuged (8000 rpm, 5 min, 4 °C). The supernatant was used directly for HPLC analysis. Conversions determined using Method A, enantiomeric excess of D-enantiomer determined using Method B (**2a**), Method C (**2b**) and Method D (**2h**-**2l**).

Table S19. Hydroamination of **1a-1b** and **1h-1l** acid at 2.5 mM substrate concentration with PAL variants.

Substrate	*Pb*PAL WT		*Pb*PAL L205F-K397A		*Pb*PAL K397A		*Pb*PAL L205V-K397A	
	**conv., %**	***ee*, %[a]**	**conv., %**	***ee*, %[a]**	**conv., %**	***ee*, %[a]**	**conv., %**	***ee*, %[a]**
**1a**	99	-5	96	98	99	-11	99	-7
**1b**	95	-55	15	-7	87	-79	97	-94
**1h**	99	-71	10	23	99	-99	99	-99.5
**1i**	99	-2	59	55	99	-61	99	-75
**1j**	99	-7	25	27	99	-77	99	-95
**1k**	99	-59	34	16	99	-99	99	-99.5
**1l**	99	-2	52	54	99	-75	99	-82

[a]*ee* of the D-enantiomer

7. Preparative and semi-preparative scale hydroaminations

Preparative scale hydroamination of 1a

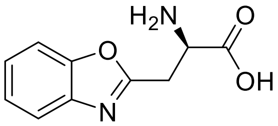

2a

To a solution of the substrate **1a** (1.13 g, 6.0 mmol, 1.0 equiv.) in 6 mL of DMSO in a 250 mL conical flask, 100 mL of ammonium carbonate buffer (3.6 M, pH 9.5) and a solution of *Pb*PAL L205F (28 mg, 2.5 wt%) in 14 mL of storage buffer (100 mM phosphate, NaCl 300 mM, pH 8.0) were added. The suspension was incubated in an orbital shaker at 300 rpm, 30 °C for 36 h. Then the reaction was quenched by adding methanol (100 mL), and the mixture was centrifuged (8000 rpm, 10 min, 4 °C) to remove the insoluble components. The aqueous phase was extracted with diethyl ether (3 × 40 mL) and the solvent evaporated under vacuum until dryness. The resulting solid was extracted with a 1:1 acetone-methanol mixture (4 × 50 mL), filtered with 0.22 µm PTFE syringe filter, and dried under vacuum to afford the desired product **2a** (1.14 g, 85%) as a beige solid. Characterization data are reported below (Table S20).

Preparative scale hydroamination of 1k

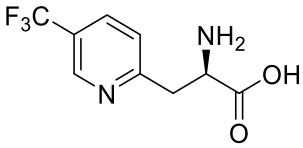

2k

To a solution of the substrate **1k** (1.09 g, 5.0 mmol, 1.0 equiv.) in 5 mL of DMSO in a 250 mL conical flask, 67 mL of ammonium carbonate buffer (4.6 M, pH 9.5) and a solution of *Pb*PAL L205F (109 mg, 10.0 wt%) in 28 mL of storage buffer (100 mM phosphate, NaCl 300 mM, pH 8.0) were added. The suspension was incubated in an orbital shaker at 300 rpm, 30 °C for 24 h, cooled to room temperature. Then reaction mixture was acidified to pH < 2.0 by the addition of aqueous sulfuric acid (50% w/v) on ice (caution: carbon dioxide gas evolution), the aqueous phase was extracted with diethyl ether (3 × 20 mL), and filtered using 0.22 µm PTFE syringe filter. Dowex® 50WX8 resin hydrogen form (7 g) was washed with Mili-Q water (20 mL) and aqueous sulfuric acid (20 mL, 10% w/v). The aqueous phase was loaded onto the resin, incubated for 10 min at room temperature. Then the resin was washed with water until neutral pH of the eluent, and the product was eluted using aqueous NH_4OH (10% w/v). The solvent was removed under vacuum to afford **2k** (0.88 g, 70%) as a white solid. Characterization data are reported below (Table S20).

Semi-preparative hydroamination of 1b

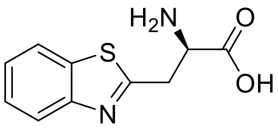

2b

In a 20 mL glass vial equipped with a screw cap, substrate **1b** (21 mg, 0.1 mmol) was added to a solution of ammonium carbonate (1.3 mL, 4.6 M, pH 9.5), followed by the addition of *Pb*PAL L205Y (2 mg) in 0.7 mL of storage buffer (100 mM phosphate, 300 mM NaCl, pH 8.0), to give a final substrate concentration of 50 mM. The mixture was incubated in an orbital shaker (300 rpm, 30 °C) for 36 h. Then, the reaction mixture was acidified to pH < 2.0 by addition of aqueous sulfuric acid (10% w/v) on ice (caution: carbon dioxide gas evolution). The aqueous phase was extracted with diethyl ether (2 × 5 mL) and filtered using 0.22 µm PTFE syringe filter. Dowex® 50WX8 resin hydrogen form (1.0 g) was washed with water (20 mL) and aqueous sulfuric acid (10 mL, 10% w/v). The aqueous phase was loaded onto the resin and incubated for 10 min at room temperature. Then, the resin was washed with water until neutral pH of the eluent and the product was eluted using aqueous NH_4OH (10 mL, 10% w/v). The solvent was removed under vacuum to afford **2b** (9mg, 38%) as a white solid**.** Characterization data are reported below (Table S20).

General protocol for semi-preparative scale hydroaminations of 1c-1f

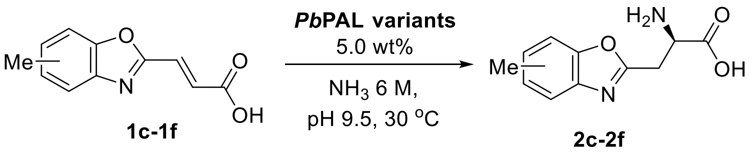

In a 20 mL glass vial equipped with a screw cap, substrates **1c-1f** (20 mg, 0.1 mmol) were added to a solution of ammonium carbonate (1.3 mL, 4.6 M, pH 9.5), followed by the addition of *Pb*PAL variant (2 mg) in 0.7 mL of storage buffer (100 mM phosphate, 300 mM NaCl, pH 8.0), to give a final substrate concentration of 50 mM. The mixture was incubated in an orbital shaker (300 rpm, 30 °C) for 36 h. Then, the reaction was quenched by adding methanol (2 mL), and the mixture was centrifuged (8000 rpm, 10 min, 4 °C) to remove the insoluble components. The aqueous phase was extracted with diethyl ether (2 × 4 mL) and the solvent evaporated under vacuum until dryness. The resulting solid was extracted with a 1:1 acetone-methanol mixture (4 × 5 mL), filtered with 0.22 μm PTFE syringe filter, and dried under vacuum to afford the desired products **2c-2f.** Characterization data are reported below (Table S20).

Semi-preparative scale hydroamination of 1g

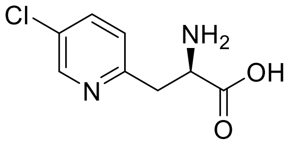

2g

In a 20 mL glass vial equipped with a screw cap, substrate **1g** (18.5 mg, 0.1 mmol) was added to a solution of ammonium carbonate (1.3 mL, 4.6 M, pH 9.5), followed by the addition of *Pb*PAL L205F (1.8 mg) in 0.7 mL of storage buffer (100 mM phosphate, 300 mM NaCl, pH 8.0), to give a final substrate concentration of 50 mM. The mixture was incubated in an orbital shaker (300 rpm, 30 °C) for 24 h. Then, the reaction mixture was acidified to pH < 2.0 by addition of aqueous sulfuric acid (10% w/v) on ice (caution: carbon dioxide gas evolution). The aqueous phase was extracted with diethyl ether (2 × 5 mL) and filtered using 0.22 μm PTFE syringe filter. Dowex® 50WX8 resin hydrogen form (1.0 g) was washed with water (10 mL) and aqueous sulfuric acid (10 mL, 10% w/v). The aqueous phase was loaded onto the resin and incubated for 10 min at room temperature. Then, the resin was washed with water until neutral pH of the eluent and the product was eluted using aqueous NH_4OH (10 mL, 10% w/v). The solvent was removed under vacuum to afford **2g** (11 mg, 51%) as a white solid**.** Characterization data are reported below (Table S20).

Semi-preparative scale hydroamination of 1h

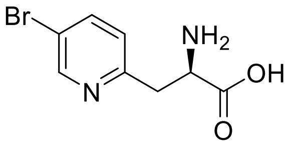

2h

In a 20 mL glass vial equipped with a screw cap, substrate **1h** (23 mg, 0.1 mmol) was added to a solution of ammonium carbonate (1.3 mL, 4.6 M, pH 9.5), followed by the addition of *Pb*PAL L205F (2.3 mg) in 0.7 mL of storage buffer (100 mM phosphate, 300 mM NaCl, pH 8.0), to give a final substrate concentration of 50 mM. The mixture was incubated in an orbital shaker (300 rpm, 30 °C) for 24 h. Then, the reaction mixture was acidified to pH < 2.0 by addition of aqueous sulfuric acid (10% w/v) on ice (caution: carbon dioxide gas evolution). The aqueous phase was extracted with diethyl ether (2 × 5 mL) and filtered using 0.22 μm PTFE syringe filter. Dowex® 50WX8 resin hydrogen form (1.0 g) was washed with water (10 mL) and aqueous sulfuric acid (10 mL, 10% w/v). The aqueous phase was loaded onto the resin and incubated for 10 min at room temperature. Then, the resin was washed with water until neutral pH of the eluent and the product was eluted using aqueous NH_4OH (10 mL, 10% w/v). The solvent was removed under vacuum to afford **2h** (18 mg, 69%) as a white solid**.** Characterization data are reported below (Table S20).

Semi-preparative scale hydroamination of 1i

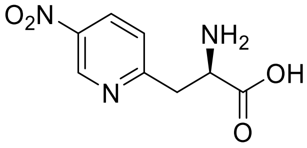

2i

In a 20 mL glass vial equipped with a screw cap, substrate **1i** (19.5 mg, 0.1 mmol) was added to a solution of ammonium carbonate (1.3 mL, 4.6 M, pH 9.5), followed by the addition of *Pb*PAL L205F (1.0 mg) in 0.7 mL of storage buffer (100 mM phosphate, 300 mM NaCl, pH 8.0), to give a final substrate concentration of 50 mM. The mixture was incubated in an orbital shaker (300 rpm, 30 °C) for 24 h. Then, the reaction mixture was acidified to pH < 2.0 by addition of aqueous sulfuric acid (10% w/v) on ice (caution: carbon dioxide gas evolution). The aqueous phase was extracted with diethyl ether (2 × 5 mL) and filtered using 0.22 μm PTFE syringe filter. Dowex® 50WX8 resin hydrogen form (1.0 g) was washed with water (10 mL) and aqueous sulfuric acid (10 mL, 10% w/v). The aqueous phase was loaded onto the resin and incubated for 10 min at room temperature. Then, the resin was washed with water until neutral pH of the eluent and the product was eluted using aqueous NH_4OH (10 mL, 10% w/v). The solvent was removed under vacuum to afford **2i** (17.5 mg, 77%) as a beige solid**.** Characterization data are reported below (Table S20).

Semi-preparative scale hydroamination of 1k

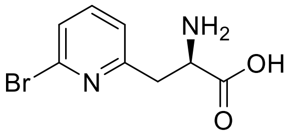

2k

In a 20 mL glass vial equipped with a screw cap, substrate **1k** (23 mg, 0.1 mmol) was added to a solution of ammonium carbonate (1.3 mL, 4.6 M, pH 9.5), followed by the addition of *Pb*PAL L205F (2.3 mg) in 0.7 mL of storage buffer (100 mM phosphate, 300 mM NaCl, pH 8.0), to give a final substrate concentration of 50 mM. The mixture was incubated in an orbital shaker (300 rpm, 30 °C) for 24 h. Then, the reaction mixture was acidified to pH < 2.0 by addition of aqueous sulfuric acid (10% w/v) on ice (caution: carbon dioxide gas evolution). The aqueous phase was extracted with diethyl ether (2 × 5 mL) and filtered using 0.22 μm PTFE syringe filter. Dowex® 50WX8 resin hydrogen form (1.0 g) was washed with water (10 mL) and aqueous sulfuric acid (10 mL, 10% w/v). The aqueous phase was loaded onto the resin and incubated for 10 min at room temperature. Then, the resin was washed with water until neutral pH of the eluent and the product was eluted using aqueous NH_4OH (10 mL, 10% w/v). The solvent was removed under vacuum to afford **2k** (17.5 mg, 68%) as a white solid**.** Characterization data are reported below (Table S20).

Semi-preparative scale hydroamination of 1l

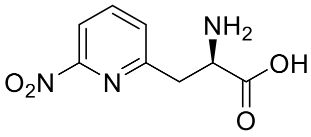

2l

In a 20 mL glass vial equipped with a screw cap, substrate **1l** (19.5 mg, 0.1 mmol) was added to a solution of ammonium carbonate (1.3 mL, 4.6 M, pH 9.5), followed by the addition of *Pb*PAL L205F (1.0 mg) in 0.7 mL of storage buffer (100 mM phosphate, 300 mM NaCl, pH 8.0), to give a final substrate concentration of 50 mM. The mixture was incubated in an orbital shaker (300 rpm, 30 °C) for 24 h. Then, the reaction mixture was acidified to pH < 2.0 by addition of aqueous sulfuric acid (10% w/v) on ice (caution: carbon dioxide gas evolution). The aqueous phase was extracted with diethyl ether (2 × 5 mL) and filtered using 0.22 μm PTFE syringe filter. Dowex® 50WX8 resin hydrogen form (1.0 g) was washed with water (10 mL) and aqueous sulfuric acid (10 mL, 10% w/v). The aqueous phase was loaded onto the resin and incubated for 10 min at room temperature. Then, the resin was washed with water until neutral pH of the eluent and the product was eluted using aqueous NH_4OH (10 mL, 10% w/v). The solvent was removed under vacuum to afford **2l** (14 mg, 62%) as a beige solid**.** Characterization data are reported below (Table S20).

Semi-preparative scale hydroamination of 1m

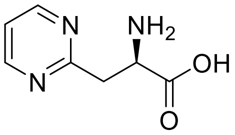
2m

In a 20 mL glass vial equipped with a screw cap, substrate **1m** (15 mg, 0.1 mmol) was added to a solution of ammonium carbonate (1.3 mL, 4.6 M, pH 9.5), followed by the addition of *Pb*PAL L205F (0.8 mg) in 0.7 mL of storage buffer (100 mM phosphate, 300 mM NaCl, pH 8.0), to give a final substrate concentration of 50 mM. The mixture was incubated in an orbital shaker (300 rpm, 30 °C) for 24 h. Then, the reaction mixture was acidified to pH < 2.0 by addition of aqueous sulfuric acid (10% w/v) on ice (caution: carbon dioxide gas evolution). The aqueous phase was extracted with diethyl ether (3 × 5 mL) and filtered using 0.22 μm PTFE syringe filter. Dowex® 50WX8 resin hydrogen form (1.0 g) was washed with water (10 mL) and aqueous sulfuric acid (10 mL, 10% w/v). The aqueous phase was loaded onto the resin and incubated for 10 min at room temperature. Then, the resin was washed with water until neutral pH of the eluent and the product was eluted using aqueous NH_4OH (10 mL, 10% w/v). The solvent was removed under vacuum to afford **2m** (9 mg, 49%) as a white solid**.** Characterization data are reported below (Table S20).

Semi-preparative scale hydroamination of 1n

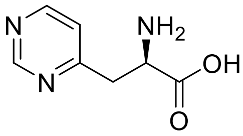
2n

In a 20 mL glass vial equipped with a screw cap, substrate **1n** (15 mg, 0.1 mmol) was added to a solution of ammonium carbonate (1.3 mL, 4.6 M, pH 9.5), followed by the addition of *Pb*PAL L205F (0.8 mg) in 0.7 mL of storage buffer (100 mM phosphate, 300 mM NaCl, pH 8.0), to give a final substrate concentration of 50 mM. The mixture was incubated in an orbital shaker (300 rpm, 30 °C) for 24 h. Then, the reaction mixture was acidified to pH < 2.0 by addition of aqueous sulfuric acid (10% w/v) on ice (caution: carbon dioxide gas evolution). The aqueous phase was extracted with diethyl ether (3 × 5 mL) and filtered using 0.22 μm PTFE syringe filter. Dowex® 50WX8 resin hydrogen form (1.0 g) was washed with water (10 mL) and aqueous sulfuric acid (10 mL, 10% w/v). The aqueous phase was loaded onto the resin and incubated for 10 min at room temperature. Then, the resin was washed with water until neutral pH of the eluent and the product was eluted using aqueous NH_4OH (10 mL, 10% w/v). The solvent was removed under vacuum to afford **2n** (12 mg, 65%) as a white solid**.** Characterization data are reported below (Table S20).

Semi-preparative scale hydroamination of 1o

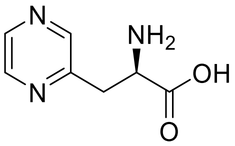
2o

In a 20 mL glass vial equipped with a screw cap, substrate **1o** (15 mg, 0.1 mmol) was added to a solution of ammonium carbonate (1.3 mL, 4.6 M, pH 9.5), followed by the addition of *Pb*PAL F93V-L205F (0.8 mg) in 0.7 mL of storage buffer (100 mM phosphate, 300 mM NaCl, pH 8.0), to give a final substrate concentration of 50 mM. The mixture was incubated in an orbital shaker (300 rpm, 30 °C) for 24 h. Then, the reaction mixture was acidified to pH < 2.0 by addition of aqueous sulfuric acid (10% w/v) on ice (caution: carbon dioxide gas evolution). The aqueous phase was extracted with diethyl ether (2 × 5 mL) and filtered using 0.22 μm PTFE syringe filter. Dowex® 50WX8 resin hydrogen form (1.0 g) was washed with water (10 mL) and aqueous sulfuric acid (10 mL, 10% w/v). The aqueous phase was loaded onto the resin and incubated for 10 min at room temperature. Then, the resin was washed with water until neutral pH of the eluent and the product was eluted using aqueous NH_4OH (10 mL, 10% w/v). The solvent was removed under vacuum to afford **2o** (13 mg, 71%) as a beige solid**.** Characterization data are reported below (Table S20).

Semi-preparative scale hydroamination of 1p

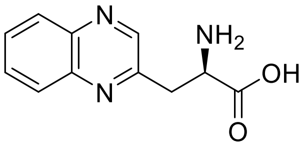

2p

In a 20 mL glass vial equipped with a screw cap, substrate **1p** (20 mg, 0.1 mmol) was added to a solution of ammonium carbonate (1.3 mL, 4.6 M, pH 9.5), followed by the addition of *Pb*PAL L90A-L205F (2 mg) in 0.7 mL of storage buffer (100 mM phosphate, 300 mM NaCl, pH 8.0), to give a final substrate concentration of 50 mM. The mixture was incubated in an orbital shaker (300 rpm, 30 °C) for 36 h. Then, the reaction mixture was acidified to pH < 2.0 by addition of aqueous sulfuric acid (10% w/v) on ice (caution: carbon dioxide gas evolution). The aqueous phase was extracted with diethyl ether (2 × 5 mL) and filtered using 0.22 μm PTFE syringe filter. Dowex® 50WX8 resin hydrogen form (1.0 g) was washed with water (10 mL) and aqueous sulfuric acid (10 mL, 10% w/v). The aqueous phase was loaded onto the resin and incubated for 10 min at room temperature. Then, the resin was washed with water until neutral pH of the eluent and the product was eluted using aqueous NH_4OH (10 mL, 10% w/v). The solvent was removed under vacuum to afford **2p** (14 mg, 60%) as a brown solid**.** Characterization data are reported below (Table S20).

Semi-preparative scale hydroamination of 1k

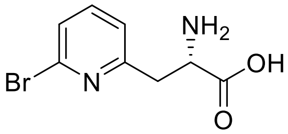

L-**2k**

In a 20 mL glass vial equipped with a screw cap, substrate **1k** (23 mg, 0.1 mmol) was added to a solution of ammonium carbonate (1.3 mL, 4.6 M, pH 9.5), followed by the addition of *Pb*PAL L205V-K397A (1 mg) in 0.7 mL of storage buffer (100 mM phosphate, 300 mM NaCl, pH 8.0), to give a final substrate concentration of 50 mM. The mixture was incubated in an orbital shaker (300 rpm, 30 °C) for 3 h. Then, the reaction mixture was acidified to pH < 2.0 by addition of aqueous sulfuric acid (10% w/v) on ice (caution: carbon dioxide gas evolution). The aqueous phase was extracted with diethyl ether (2 × 5 mL) and filtered using 0.22 μm PTFE syringe filter. Dowex® 50WX8 resin hydrogen form (2.0 g) was washed with water (10 mL) and aqueous sulfuric acid (10 mL, 10% w/v). The aqueous phase was loaded onto the resin and incubated for 10 min at room temperature. Then, the resin was washed with water until neutral pH of the eluent and the product was eluted using aqueous NH_4OH (10 mL, 10% w/v). The solvent was removed under vacuum to afford L-**2k** (16 mg, 61%) as a white solid**.** Characterization data are reported below (Table S20).

Table S20. Characterization data for compounds **2a-2p.**

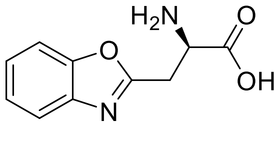	(*R*)-2-amino-3-(benzo[d]oxazol-2-yl)propanoic acid **2a:** beige solid (1.135 g, 85%). **^{1}H NMR** (400 MHz, D_2O) δ 7.71 – 7.55 (m, 3H), 7.44 – 7.33 (m, 2H), 3.79 (dd, *J* = 8.1, 5.2 Hz, 1H), 3.37 – 3.27 (m, 1H), 3.16 (dd, *J* = 15.3, 8.2 Hz, 1H). **^{13}C NMR** (101 MHz, D_2O) δ 181.7, 166.7, 151.5, 140.7, 126.2, 125.6, 119.7, 111.9, 55.2, 35.0. **HRMS** (ESI/QTOF) m/z: $[M + H]^+$ Calcd for $C_{10}H_{11}N_2O_3^+$ 207.0764. Found 207.0766. $[\alpha]_D^{23}$ = -13.4 (c = 0.5 in 1M HCl). **HPLC:** The enantiomeric excess (99%) was determined by HPLC analysis using Method B.
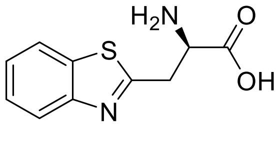	(*R*)-2-amino-3-(benzo[d]thiazol-2-yl)propanoic acid **2b**: white solid (9 mg, 38%). **^{1}H NMR** (400 MHz, D_2O) δ 7.93 (dd, *J* = 17.5, 8.1 Hz, 2H), 7.51 (t, *J* = 7.7 Hz, 1H), 7.43 (t, *J* = 7.6 Hz, 1H), 3.72 (dd, *J* = 8.1, 5.1 Hz, 1H), 3.45 (dd, *J* = 14.7, 5.1 Hz, 1H), 3.28 (dd, *J* = 14.7, 8.1 Hz, 1H). **^{13}C NMR** (101 MHz, D_2O) δ 181.7, 171.8, 152.8, 135.8, 127.4, 126.3, 123.1, 122.5, 57.4, 39.9. **HRMS** (ESI/QTOF) m/z: $[M + H]^+$ Calcd for $C_{10}H_{11}N_2O_2S^+$ 223.0536; Found 223.0540. $[\alpha]_D^{23}$ = -10.2 (c = 0.5 in 1M HCl). **HPLC:** The enantiomeric excess (95%) was determined by HPLC analysis using Method C.
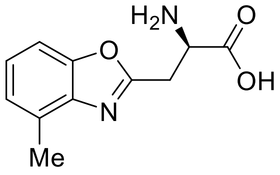	(*R*)-2-amino-3-(4-methylbenzo[d]oxazol-2-yl)propanoic acid **2c**: beige solid (16 mg, 68%). **^{1}H NMR** (400 MHz, D_2O) δ 7.46 (d, *J* = 8.2 Hz, 1H), 7.32 (t, *J* = 7.9 Hz, 1H), 7.22 (d, *J* = 7.5 Hz, 1H), 3.87 – 3.78 (m, 1H), 3.35 (dd, *J* = 15.0, 5.2 Hz, 1H), 3.18 (dd, *J* = 15.1, 8.3 Hz, 1H), 2.55 (s, 3H). **^{13}C NMR** (101 MHz, D_2O) δ 181.6, 166.0, 151.3, 139.8, 130.6, 126.0, 125.9, 109.1, 55.4, 35.0, 16.6. **HRMS** (ESI/QTOF) m/z: $[M + H]^+$ Calcd for

	$C_{11}H_{13}N_2O_3^+$ 221.0921; Found 221.0923; $[\alpha]_D^{23}$ = -12.4 (c = 0.5 in 1M HCl). **HPLC:** The enantiomeric excess (83%) was determined by HPLC analysis using Method B.
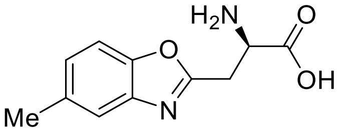	(*R*)-2-amino-3-(5-methylbenzo[d]oxazol-2-yl)propanoic acid **2d**: beige solid (20 mg, 84%). **^{1}H NMR** (400 MHz, D_2O) δ 7.51 (d, *J* = 9.4 Hz, 2H), 7.26 (d, *J* = 8.3 Hz, 1H), 3.86 – 3.78 (m, 1H), 3.34 (dd, *J* = 15.2, 5.2 Hz, 1H), 3.18 (dd, *J* = 15.1, 8.1 Hz, 1H), 2.46 (s, 3H). **^{13}C NMR** (101 MHz, D_2O) δ 181.6, 166.7, 149.6, 140.8, 135.8, 127.1, 119.4, 111.2, 55.2, 35.0, 21.4. **HRMS** (ESI/QTOF) m/z: $[M + H]^+$ Calcd for $C_{11}H_{13}N_2O_3^+$ 221.0921; Found 221.0922. $[\alpha]_D^{23}$ = -12.7 (c = 0.5 in 1M HCl). **HPLC:** The enantiomeric excess (89%) was determined by HPLC analysis using Method B.
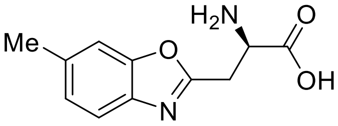	(*R*)-2-amino-3-(6-methylbenzo[d]oxazol-2-yl)propanoic acid **2e**: beige solid (14 mg, 59%). **^{1}H NMR** (400 MHz, D_2O) δ 7.55 (d, *J* = 8.1 Hz, 1H), 7.46 (s, 1H), 7.25 (d, *J* = 8.2 Hz, 1H), 3.85 – 3.77 (m, 1H), 3.32 (dd, *J* = 15.1, 5.2 Hz, 1H), 3.16 (dd, *J* = 14.0, 8.1 Hz, 1H), 2.47 (s, 3H). **^{13}C NMR** (101 MHz, D_2O) δ 181.6, 166.1, 151.8, 138.4, 137.0, 126.6, 119.0, 111.9, 55.2, 34.9, 21.6. **HRMS** (ESI/QTOF) m/z: $[M + H]^+$ Calcd for $C_{11}H_{13}N_2O_3^+$ 221.0921; Found 221.0923. $[\alpha]_D^{23}$ = -13.1 (c = 0.5 in 1M HCl). **HPLC:** The enantiomeric excess (87 %) was determined by HPLC analysis using Method B.
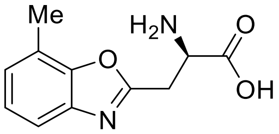	(*R*)-2-amino-3-(7-methylbenzo[d]oxazol-2-yl)propanoic acid **2f**: greyish solid (18 mg, 76%). **^{1}H NMR** (400 MHz, D_2O) δ 7.51 (d, *J* = 7.8 Hz, 1H), 7.32 (t, *J* = 7.7 Hz, 1H), 7.25 (d, *J* = 7.5 Hz, 1H), 3.89 – 3.81 (m, 1H), 3.37 (dd, *J* = 15.3, 5.2 Hz, 1H), 3.20 (dd, *J* = 15.2, 8.1 Hz, 1H), 2.53 (s, 3H). **^{13}C NMR** (101 MHz, D_2O) δ 181.6, 166.4, 150.7, 140.3, 127.0, 125.5, 122.8, 116.8, 55.2, 35.0, 15.1. **HRMS** (ESI/QTOF) m/z: $[M + H]^+$ Calcd for $C_{11}H_{13}N_2O_3^+$ 221.0921; Found 221.0921. $[\alpha]_D^{23}$ = -12.6 (c = 0.5 in 1M HCl). **HPLC:** The enantiomeric excess (94%) was determined by HPLC analysis using Method B.
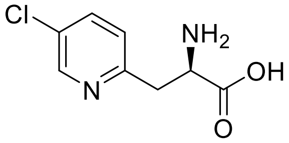	(*R*)-2-amino-3-(5-chloropyridin-2-yl)propanoic acid **2g**: white solid (11 mg, 51%). **^{1}H NMR** (400 MHz, D_2O) δ 8.41 (d, *J* = 2.4 Hz, 1H), 7.77 (dd, *J* = 8.4, 2.5 Hz, 1H), 7.28 (d, *J* = 8.4 Hz, 1H), 3.57 (dd, *J* = 8.2, 5.8 Hz, 1H), 3.10 (dd, *J* = 13.6, 5.7 Hz, 1H), 2.89 (dd, *J* = 13.6, 8.3 Hz, 1H). **^{13}C NMR** (101 MHz, D_2O) δ 182.5, 157.2, 147.9, 138.2, 130.8, 126.1, 57.6, 42.8. **HRMS** (ESI/QTOF) m/z: $[M + Na]^+$ Calcd for $C_8H_9ClN_2NaO_2^+$ 223.0245; Found 223.0246. $[\alpha]_D^{23}$ = -28.8 (c = 0.5 in 1M HCl). **HPLC:** The enantiomeric excess (84%) was determined by HPLC analysis using Method D.
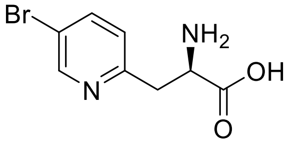	(*R*)-2-amino-3-(5-bromopyridin-2-yl)propanoic acid **2h**: white solid (18 mg, 69%). **^{1}H NMR** (400 MHz, D_2O) δ 8.51 (d, *J* = 2.2 Hz, 1H), 7.92 (dd, *J* = 8.4, 2.4 Hz, 1H), 7.23 (d, *J* = 8.4 Hz, 1H), 3.57 (dd, *J* = 8.3, 5.7 Hz, 1H), 3.08 (dd, *J* = 13.6, 5.7 Hz, 1H), 2.87 (dd, *J* = 13.6, 8.3 Hz, 1H). **^{13}C NMR** (101 MHz, D_2O) δ 182.7, 157.8, 150.3, 141.3, 126.8, 119.4, 57.7, 43.1. **HRMS** (ESI/QTOF) m/z: $[M + H]^+$ Calcd for $C_8H_{10}BrN_2O_2^+$ 244.9920; Found 244.9925. $[\alpha]_D^{23}$ = -37.1 (c = 0.5 in 1M HCl). **HPLC:** The enantiomeric excess (92%) was determined by HPLC analysis using Method D.
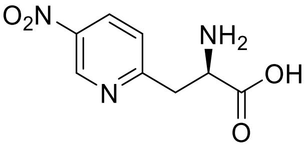	(*R*)-2-amino-3-(5-nitropyridin-2-yl)propanoic acid **2i**: beige solid (17.5 mg, 77%). **^{1}H NMR** (400 MHz, D_2O) δ 9.36 (d, *J* = 2.4 Hz, 1H), 8.61 (dd, *J* = 8.6, 2.5 Hz, 1H), 7.64 (d, *J* = 8.6 Hz, 1H), 4.28 – 4.21 (m, 1H), 3.63 – 3.47 (m, 2H). **^{13}C NMR** (101 MHz, D_2O) δ 174.1, 163.9, 145.4, 144.5, 133.8, 125.8, 54.8, 37.9. **HRMS** (ESI/QTOF) m/z: $[M + H]^+$ Calcd for $C_8H_{10}N_3O_4^+$ 212.0666; Found 212.0669. $[\alpha]_D^{23}$ = -12.5 (c = 0.5 in 1M HCl). **HPLC:** The enantiomeric excess (98%) was determined by HPLC analysis using Method D.

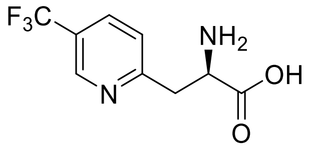	(R)-2-amino-3-(5-(trifluoromethyl)pyridin-2-yl)propanoic acid **2j**: white solid (0.88g, 70%).<br>**^{1}H NMR** (400 MHz, D_2O) δ 8.62 (s, 1H), 7.96 – 7.89 (m, 1H), 7.35 (d, J = 8.2 Hz, 1H), 3.54 – 3.45 (m, dd, J = 8.3, 5.8 Hz, 1H), 3.07 (dd, J = 13.6, 5.7 Hz, 1H), 2.86 (dd, J = 13.6, 8.4 Hz, 1H). **^{19}F NMR** (376 MHz, D_2O) δ -62.3. **^{13}C NMR** (101 MHz, D_2O) 182.6, 163.8, 146.5 (q, J = 4.0 Hz), 135.9 (q, J = 4.0 Hz), 125.6 (q, J = 32.3 Hz), 125.5, 124.6 (q, J = 272 Hz). **HRMS** (ESI/QTOF) m/z: $[M + H]^+$ Calcd for $C_9H_{10}F_3N_2O_2^+$ 235.0689; Found 235.0693. $[\alpha]_D^{23}$ = -36.9 (c = 0.5 in 1M HCl). **HPLC:** The enantiomeric excess (>99%) was determined by HPLC analysis using Method D.
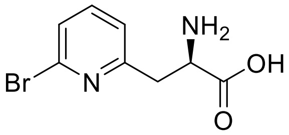	(R)-2-amino-3-(6-bromopyridin-2-yl)propanoic acid **2k**: white solid (17.5 mg, 68%).<br>**^{1}H NMR** (400 MHz, D_2O) δ 8.52 (d, J = 2.3 Hz, 1H), 7.92 (dd, J = 8.4, 2.4 Hz, 1H), 7.23 (d, J = 8.4 Hz, 1H), 3.57 (dd, J = 8.3, 5.7 Hz, 1H), 3.08 (dd, J = 13.6, 5.7 Hz, 1H), 2.87 (dd, J = 13.6, 8.3 Hz, 1H). **^{13}C NMR** (101 MHz, D_2O) δ 182.7, 157.8, 150.4, 141.3, 126.8, 119.4, 57.7, 43.1. **HRMS** (ESI/QTOF) m/z: $[M + H]^+$ Calcd for $C_8H_{10}BrN_2O_2^+$ 244.9920; Found 244.9926. $[\alpha]_D^{23}$ = -36.1 (c = 0.5 in 1M HCl). **HPLC:** The enantiomeric excess (94%) was determined by HPLC analysis using Method D.
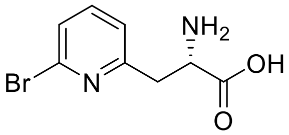	(S)-2-amino-3-(6-bromopyridin-2-yl)propanoic acid L-**2k**: white solid (16 mg, 61%).<br>**^{1}H NMR** (400 MHz, D_2O) δ 8.51 (d, J = 2.3 Hz, 1H), 7.91 (dd, J = 8.4, 2.4 Hz, 1H), 7.22 (d, J = 8.4 Hz, 1H), 3.55 (dd, J = 8.3, 5.7 Hz, 1H), 3.06 (dd, J = 13.6, 5.7 Hz, 1H), 2.85 (dd, J = 13.6, 8.3 Hz, 1H). $[\alpha]_D^{23}$ = +40.4 (c = 0.5 in 1M HCl).<br>**HPLC:** The enantiomeric excess (> - 99%) was determined by HPLC analysis using Method D.
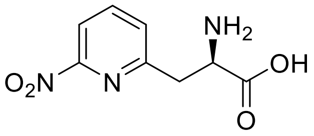	(R)-2-amino-3-(6-nitropyridin-2-yl)propanoic acid **2l**: beige solid (14 mg, 62%).<br>**^{1}H NMR** (400 MHz, D_2O) δ 9.36 (d, J = 2.6 Hz, 1H), 8.61 (dd, J = 8.6, 2.6 Hz, 1H), 7.64 (d, J = 8.6 Hz, 1H), 4.25 (dd, J = 7.0, 5.2 Hz, 1H), 3.63 – 3.47 (m, 2H). **^{13}C NMR** (101 MHz, D_2O) δ 174.1, 163.9, 145.4, 144.5, 133.8, 125.8, 54.8, 37.9. **HRMS** (ESI/QTOF) m/z: $[M - H]^-$ Calcd for $C_8H_5BrNO_2^-$ 225.9509; Found 225.9510. $[\alpha]_D^{23}$ = -15.3 (c = 0.5 in 1M HCl). **HPLC:** The enantiomeric excess (95%) was determined by HPLC analysis using Method D.
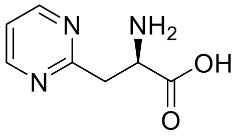	(R)-2-amino-3-(pyrimidin-2-yl)propanoic acid **2m**: white solid (9 mg, 49%).<br>**^{1}H NMR** (400 MHz, D_2O) δ 8.73 (d, J = 5.1 Hz, 2H), 7.44 (t, J = 5.1 Hz, 1H), 3.75 (dd, J = 9.0, 5.5 Hz, 1H), 3.33 (dd, J = 14.0, 5.5 Hz, 1H), 3.06 (dd, J = 14.0, 9.0 Hz, 1H). **^{13}C NMR** (101 MHz, D_2O) δ 182.6, 168.3, 158.6, 120.7, 57.0, 44.6. **HRMS** (ESI/QTOF) m/z: $[M + H]^+$ Calcd for $C_7H_{10}N_3O_2^+$ 168.0768; Found 168.0770. $[\alpha]_D^{23}$ = -15.5 (c = 0.5 in 1M HCl). **HPLC:** The enantiomeric excess (90%) was determined by HPLC analysis using Method D.
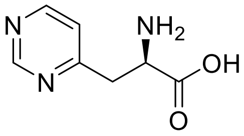	(R)-2-amino-3-(pyrimidin-4-yl)propanoic acid **2n**: white solid (12 mg, 65%)<br>**^{1}H NMR** (400 MHz, D_2O) δ 9.04 (s, 1H), 8.68 (d, J = 5.3 Hz, 1H), 7.49 (d, J = 5.3 Hz, 1H), 3.89 – 3.81 (m, 1H), 3.25 (dd, J = 14.5, 5.5 Hz, 1H), 3.09 (dd, J = 14.5, 8.0 Hz, 1H). **^{13}C NMR** (101 MHz, D_2O) δ 179.6, 168.4, 158.2, 157.9, 123.1, 56.2, 41.4. **HRMS** (ESI/QTOF) m/z: $[M - H]^-$ Calcd for $C_7H_8N_3O_2^-$ 166.0617; Found 166.0620.<br>$[\alpha]_D^{23}$ = -17.3 (c = 0.5 in 1M HCl). **HPLC:** The enantiomeric excess (90%) was determined by HPLC analysis using Method D.
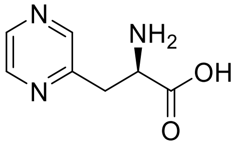	(R)-2-amino-3-(pyrazin-2-yl)propanoic acid **2o**: beige solid (13 mg, 71%)<br>**^{1}H NMR** (400 MHz, D_2O) δ 8.58 – 8.52 (m, 2H), 8.49 (d, J = 2.7 Hz, 1H), 3.66 (dd, J = 8.1, 5.9 Hz, 1H), 3.20 (dd, J = 13.9, 5.8 Hz, 1H), 3.03 (dd, J = 13.9, 8.1 Hz, 1H). **^{13}C NMR** (101 MHz, D_2O) δ 182.4, 155.5, 145.8, 144.9, 143.2, 57.4, 41.0. **HRMS** (ESI/QTOF) m/z: $[M + Na]^+$ Calcd for $C_7H_9N_3NaO_2^+$ 190.0587; Found 190.0585.<br>$[\alpha]_D^{23}$ = -17.8 (c = 0.5 in 1M HCl). **HPLC:** The enantiomeric excess (87%) was determined by HPLC analysis using Method D.

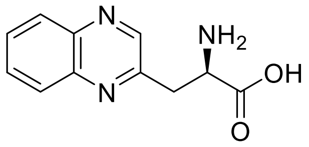	(R)-2-amino-3-(quinoxalin-2-yl)propanoic acid **2p**: brownish solid (14 mg, 60%) **^{1}H NMR** (400 MHz, D_2O) δ 8.54 (s, 1H), 7.82 – 7.62 (m, 6H), 3.66 (dd, J = 8.3, 5.8 Hz, 1H), 3.24 (dd, J = 13.8, 5.8 Hz, 1H), 3.03 (dd, J = 13.8, 8.4 Hz, 1H). **^{13}C NMR** (101 MHz, D_2O) δ 182.1, 155.5, 146.7, 141.6, 140.6, 131.8, 131.1, 128.7, 128.3, 57.5, 42.0. **HRMS** (ESI/QTOF) m/z: [M + H]$^+$ Calcd for $C_{11}H_{12}N_3O_2^+$ 218.0924; Found 218.0923. $[\alpha]_D^{23}$ = -25.2 (c = 0.5 in 1M HCl). **HPLC:** The enantiomeric excess (84%) was determined by HPLC analysis using Method E.

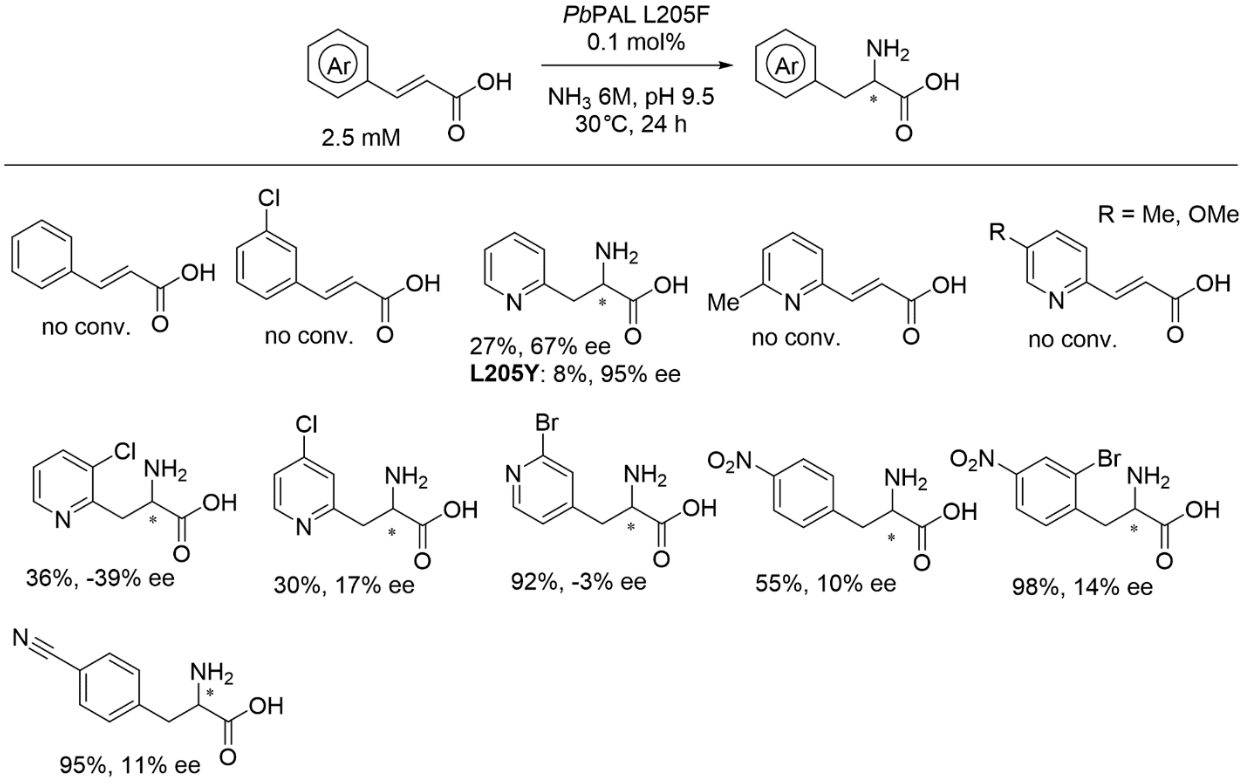

Figure S11. Limitations of hydroaminations with PbPAL L205F. **Conditions**: substrates (2.5 mM), 0.1 mol% enzyme loading, NH_3 6M, pH 9.5, 30 °C, 24 h.

8. X-ray crystallography

Table S21. Crystallographic data for compound **2a**.

**Compound**	**2a**
Formula	$C_{10.5}H_{12}N_2O_{3.5}$
$D_{calc.}$/ g cm^{-3}	1.410
μ/mm^{-1}	0.903
Formula Weight	222.22
Colour	colourless
Shape	plate-shaped
Size/mm^3	0.14×0.10×0.01
T/K	140.00(10)
Crystal System	monoclinic
Flack Parameter	-0.17(17)
Space Group	$C2$
a/Å	9.1937(4)
b/Å	7.7007(3)
c/Å	15.4070(6)
α/°	90
β/°	106.389(4)
γ/°	90
V/Å^3	1046.47(7)
Z	4
Z'	1
Wavelength/Å	1.54184
Radiation type	Cu$K\alpha$
Θ_{min}/°	2.990
Θ_{max}/°	74.405
Measured Refl's.	6299
Indep't Refl's	2011
Refl's I≥2σ(I)	1818
R_{int}	0.0301
Parameters	176
Restraints	73
Largest Peak/e Å^{-3}	0.211
Deepest Hole/e Å^{-3}	-0.211
GooF	1.063
wR_2 (all data)	0.1075
wR_2	0.1049
R_1 (all data)	0.0455
R_1	0.0408
CCDC number	2410915

Structure Quality Indicators[5–8]

**Reflections:**	d min (CuKα) 2Θ=148.8° 0.80	I/σ(I) 29.5	Rint m=3.13 3.01%	Full 135.4° 96% to 148.8° 99.7	
**Refinement:**	Shift 0.000	Max Peak 0.2	Min Peak -0.2	GooF 1.063	Hooft -.17(17)

A colourless plate-shaped crystal with dimensions 0.14 × 0.10 × 0.01 mm^3 was mounted. Data were collected using an XtaLAB Synergy R, DW system, HyPix-Arc 150 diffractometer operating at T = 140.00(10) K.

Data were measured using *w* scans with CuK_a radiation. The diffraction pattern was indexed and the total number of runs and images was based on the strategy calculation from the program CrysAlisPro system (CCD 43.134a 64-bit (release 09-08-2024)). The maximum resolution achieved was Q = 74.405° (0.80 Å).

The unit cell was refined using CrysAlisPro 1.171.43.130a (Rigaku OD, 2024) on 3247 reflections, 52% of the observed reflections.

Data reduction, scaling and absorption corrections were performed using CrysAlisPro 1.171.43.130a (Rigaku OD, 2024). The final completeness is 99.70 % out to 74.405° in Q. A Gaussian absorption correction was performed using CrysAlisPro 1.171.43.130a (Rigaku Oxford Diffraction, 2024) Numerical absorption correction based on Gaussian integration over a multifaceted crystal model. Empirical absorption correction using spherical harmonics as implemented in SCALE3 ABSPACK scaling algorithm. The absorption coefficient m of this material is 0.903 mm^{-1} at this wavelength (l = 1.54184Å) and the minimum and maximum transmissions are 0.702 and 1.000.

The structure was solved in the space group *C*2 (# 5) by the ShelXT 2018/2 (Sheldrick, 2015) structure solution program using dual methods and refined by full matrix least squares minimisation on $\boldsymbol{F}^2$ using version 2019/3 of ShelXL (Sheldrick, 2015). All non-hydrogen atoms were refined anisotropically. Hydrogen atom positions were calculated geometrically and refined using the riding model.

There is a single formula unit in the asymmetric unit, which is represented by the reported sum formula. In other words: Z is 4 and Z' is 1. The moiety formula is C10 H10 N2 O3, 0.5 (C H4 O).

The Flack parameter was refined to -0.17(17). Determination of absolute structure using Bayesian statistics on Bijvoet differences using the Olex2 results in None. The chiral atoms in this structure are: C2(R), C5(S), C6(R). Note: The Flack parameter is used to determine chirality of the crystal studied, the value should be near 0, a value of 1 means that the stereochemistry is wrong and the model should be inverted. A value of 0.5 means that the crystal consists of a racemic mixture of the two enantiomers.

Table S22. Crystallographic data for compound **2i**.

**Compound**	**2i**
Formula	$C_8H_9N_3O_4$
$D_{calc.}$/ g cm^{-3}	1.555
μ/mm^{-1}	1.091
Formula Weight	211.18
Colour	colourless
Shape	plate-shaped
Size/mm^3	0.11×0.09×0.01
T/K	139.99(10)
Crystal System	orthorhombic
Flack Parameter	0.28(17)
Space Group	$P2_12_12_1$
a/Å	5.1614(2)
b/Å	5.9970(2)
c/Å	29.1396(12)
α/°	90
β/°	90
γ/°	90
V/Å^3	901.96(7)
Z	4
Z'	1
Wavelength/Å	1.54184
Radiation type	Cu$K\alpha$
Θ_{min}/°	3.033
Θ_{max}/°	75.024
Measured Refl's.	5822
Indep't Refl's	1760
Refl's I≥2σ(I)	1633
R_{int}	0.0215
Parameters	262
Restraints	467
Largest Peak/e Å^{-3}	0.180
Deepest Hole/e Å^{-3}	-0.189
GooF	1.086
wR_2 (all data)	0.1067
wR_2	0.1048
R_1 (all data)	0.0455
R_1	0.0418
CCDC number	2410916

Structure Quality Indicators[5–8]

**Reflections:**	d min (CuKα) 2Θ=150.0° 0.80	I/σ(I) 38.3	Rint m=3.35 2.15%	Full 135.4° 97% to 150.0° 99.9	
**Refinement:**	Shift 0.000	Max Peak 0.2	Min Peak -0.2	GooF 1.086	Hooft .28(17)

A colourless plate-shaped crystal with dimensions 0.11 × 0.09 × 0.01 mm^3 was mounted. Data were collected using an XtaLAB Synergy R, DW system, HyPix-Arc 150 diffractometer operating at T = 139.99(10) K.

Data were measured using w scans with CuK_a radiation. The diffraction pattern was indexed and the total number of runs and images was based on the strategy calculation from the program CrysAlisPro system (CCD 43.137a 64-bit (release 10-09-2024)). The maximum resolution that was achieved was Q = 75.024° (0.80 Å).

The unit cell was refined using CrysAlisPro 1.171.43.135a (Rigaku OD, 2024) on 2472 reflections, 42% of the observed reflections.

Data reduction, scaling and absorption corrections were performed using CrysAlisPro 1.171.43.135a (Rigaku OD, 2024). The final completeness is 99.90 % out to 75.024° in Q. A Gaussian absorption correction was performed using CrysAlisPro 1.171.43.135a (Rigaku Oxford Diffraction, 2024) Numerical absorption correction based on Gaussian integration over a multifaceted crystal model. Empirical absorption correction using spherical harmonics as implemented in SCALE3 ABSPACK scaling algorithm. The absorption coefficient m of this material is 1.091 mm^{-1} at this wavelength (l = 1.54184Å) and the minimum and maximum transmissions are 0.869 and 1.000.

The structure was solved in the space group $P2_12_12_1$ (# 19) by the ShelXT 2018/2 (Sheldrick, 2015) structure solution program using dual methods and refined by full matrix least squares minimisation on $\boldsymbol{F^2}$ using version 2019/3 of ShelXL (Sheldrick, 2015). All non-hydrogen atoms were refined anisotropically. Hydrogen atom positions were calculated geometrically and refined using the riding model.

There is a single formula unit in the asymmetric unit, which is represented by the reported sum formula. In other words: Z is 4 and Z' is 1. The moiety formula is C8 H9 N3 O4.

The Flack parameter was refined to 0.28(17). Determination of absolute structure using Bayesian statistics on Bijvoet differences using the Olex2 results in None. The chiral atoms in this structure are: C2A(R), C2B(S). Note: The Flack parameter is used to determine chirality of the crystal studied, the value should be near 0, a value of 1 means that the stereochemistry is wrong and the model should be inverted. A value of 0.5 means that the crystal consists of a racemic mixture of the two enantiomers.

9. Molecular docking simulations

Molecular docking simulations were performed using AutoDockTools software suite (version 1.5.7).[9,10] The receptor structure was obtained based on the Cryo-EM structure of *Pb*PAL wt with bound (*R*)-APEP (PDB: 9EQ5). A point mutation at position L205F was introduced using the Mutagenesis Wizard in PyMOL (version 3.1), followed by energy minimization of the side chain conformation. In the preparation step, non-polar hydrogens were merged, and Gasteiger charges were added. All water molecules and (*R)*-APEP were removed from the receptor structure. The structure of **1a** was drawn and optimized using Pymol (version 3.1) and subsequently converted to PDB format. The molecular docking calculations were performed using flexible ligand and rigid-receptor docking. The search space was defined by embedding the binding site residues and the MIO residue. In both cases the receptor grid was defined as a cubic box with the dimension of 28 Å × 28 Å× 28 Å. The exhaustiveness search parameter of Vina was set to 100. The docking protocol generated 5 poses, and these poses were ranked based on their predicted binding affinities.

Poses 1 and 3, corresponding to the pro-(*R*) and pro-(*S*) conformations respectively, are consistent with the known binding mode of arylacrylic acids in the PAL catalytic pocket.

Table S23. Predicted binding modes, affinities and rmsds of **1a** with *Pb*PAL L205F.

**mode**	**Affinity (kcal/mol)**	**dist from best mode**	
		rmsd l.b.	rmsb u.b
**1**	-6.0	0.000	0.000
**2**	-5.9	0.891	1.751
**3**	-4.3	0.938	1.256
**4**	-3.1	1.908	6.087
**5**	-3.1	2.079	6.261

10. QM cluster calculation

The calculations presented here were performed using the Gaussian 09 program[11] with the B3LYP hybrid density functional method. Geometries were optimized with the 6-31g(d) basis set with the SMD solvation model[12] to consider the effects of the enzyme surroundings. In this method, the rest of the enzyme, that is not included in the model, is approximated by a polarizable homogeneous medium with some dielectric constant, here set to ε = 4. Thus, the model does not account for the heterogeneity of the enzyme surroundings. Dispersion corrections were obtained using the DFT-D3(BJ) method.[13] The Cryo-EM structure of *Pb*PAL (PDB: 9EQ5) was selected to construct an active site model of *Pb*PAL L205F for investigating the mechanistic basis for the observed enantioselectivity of hydroaminations. This model includes the substrate ((E)-3-(pyridin-2-yl)acrylic acid) and the following residues that form the active site pocket: MIO, Y64, G71, L90, F93, F205, N209, Y292, R295, N325, F341, K397, E426, N429, Q430.

***Pb*PAL_L205F_(*E*)-3-(pyridin-2-yl)acrylic acid_pro-(*R*)**

C	0	0.17082900	-0.49599200	-0.50155100
N	0	2.64328300	1.86620100	0.74392100
O	0	-1.73094200	-1.76918000	-1.01125200
C	0	2.20194700	0.65488500	0.33313700
C	0	2.97848200	-0.50725900	0.50075100
O	0	-1.89811400	0.46152600	-1.24286300
C	0	4.20646200	-0.41889100	1.14514200
C	0	4.64793700	0.82780100	1.59132200
C	0	3.83560200	1.93543400	1.35088100
C	0	0.85375900	0.63231100	-0.25399800
C	0	-1.24668400	-0.57737100	-0.95716400
H	0	2.62323900	-1.46801400	0.14646200
H	0	4.81054900	-1.30809000	1.29545300
H	0	5.60101600	0.94343300	2.09505800
H	0	4.15346700	2.92774500	1.66392800
C	-1	-0.91402100	-7.52911700	-6.81069800
C	0	-2.01205500	-6.71011700	-6.12109300
C	0	-1.42839000	-5.72367800	-5.13944000
C	0	-0.70624800	-4.61574000	-5.60412900
C	0	-1.53038100	-5.91363000	-3.75641100
C	0	-0.12224400	-3.71184300	-4.72192000
C	0	-0.94657700	-5.01889100	-2.85811500
C	0	-0.26029700	-3.90700200	-3.34662100
O	0	0.28415300	-2.97319300	-2.48275900
H	0	-0.42391100	-2.66640300	-1.85855300
C	-1	3.18950100	-1.72373100	-4.55052000
C	0	2.38276400	-0.46566700	-4.45083700
O	0	2.78032700	0.60635800	-4.97223300
N	0	1.22445600	-0.56759000	-3.79767500
C	-1	0.27984300	0.52059100	-3.71670800
H	0	0.96540600	-1.45288200	-3.35984100
C	-1	10.01698100	-3.24175900	0.01511800
C	0	8.52265800	-3.54083200	-0.15152200
C	0	7.69644200	-3.49187500	1.14742400
C	0	7.71798600	-2.10803900	1.80776700
C	0	6.24970300	-3.92816700	0.87738000
C	-1	12.29016700	2.05374700	1.06701400
C	0	11.49568100	2.67832100	-0.09125500
C	0	10.12656000	2.05749500	-0.25380000
C	0	8.98827800	2.65891800	0.30071900
C	0	9.97238200	0.83936400	-0.93217600
C	0	7.73104300	2.05967500	0.18714200
C	0	8.71829800	0.23945900	-1.05400000
C	0	7.59227000	0.84650800	-0.49070400
C	-1	-3.22324300	1.91784300	6.45672600
C	0	-0.35433800	0.79649300	4.40118900
C	-1	-1.37696700	-0.17695200	7.73123800
C	0	0.19016700	0.46984300	3.05310000
C	0	-2.01703300	1.31018400	5.84069000
N	0	-0.48600700	-0.67530500	2.39227000
C	0	-0.12370600	0.05567900	5.56270800
N	0	-1.53255900	1.53360400	4.61448900
O	0	0.76371700	-0.78027700	5.91349200
N	0	-1.20003600	0.41914100	6.43146800
H	0	-1.76229100	2.37776300	4.07252500
H	0	1.24980000	0.21907700	3.17344100

H	0	0.14008800	1.32042400	2.37229700
C	-1	4.33827500	-7.46238200	2.14867200
C	0	2.91477500	-7.24681600	2.66413600
C	0	2.04878600	-6.27350600	1.88414000
C	0	2.55910700	-5.37059300	0.94129800
C	0	0.66791500	-6.25096800	2.13925600
C	0	1.71602100	-4.46417400	0.28774000
C	0	-0.17157100	-5.33708500	1.50575900
C	0	0.35122800	-4.43345500	0.57760900
H	0	4.92905900	-6.54049300	2.19052700
H	0	2.96285700	-6.89923900	3.70652200
H	0	4.85018500	-8.21315300	2.75975000
H	0	3.61988500	-5.36863400	0.70987400
H	0	0.25247500	-6.95768800	2.85342400
H	0	2.12841200	-3.77697500	-0.44619000
H	0	-1.23267700	-5.32568100	1.73878600
H	0	-0.29970400	-3.70816700	0.10282400
C	-1	0.89332600	-5.18064900	5.59743000
C	0	1.75160700	-4.15771400	4.86412800
C	0	0.92337900	-3.26218800	3.96296300
N	0	1.59536400	-2.56524700	3.03139900
O	0	-0.30787100	-3.14843200	4.12312900
H	0	1.05920000	-1.97220600	2.39280500
H	0	2.55556900	-2.78853700	2.80878500
C	-1	-4.88765900	-3.62498400	6.40183100
C	0	-3.88007400	-2.48974300	6.16216400
C	0	-4.19186600	-1.77198400	4.87247600
C	0	-5.25602000	-0.85995300	4.81093100
C	0	-3.49586700	-2.07380500	3.69628600
C	0	-5.62640900	-0.27891900	3.59959000
C	0	-3.85839000	-1.48769300	2.48162600
C	0	-4.93002000	-0.59530300	2.43012200
C	-1	1.18288800	7.82065500	-0.79661900
C	0	1.84957400	6.95747100	0.27694000
C	0	2.70284100	5.82697100	-0.31797400
C	0	1.89910300	4.90492800	-1.23482200
O	0	0.87180300	4.34235600	-0.74503100
O	0	2.28897700	4.74699400	-2.43321500
C	-1	-3.48263200	6.09274000	-3.41915500
C	0	-2.19545400	5.35323400	-3.05024400
C	0	-2.39048000	3.85871300	-2.82495300
N	0	-1.35754800	3.21111400	-2.25636500
O	0	-3.43222000	3.27060400	-3.18078700
H	0	-1.46891600	2.22823100	-2.00407200
H	0	-0.54899400	3.70397300	-1.86462900
C	-1	-3.51599800	6.33536900	0.13991900
C	0	-2.90468600	5.77474900	1.41857800
C	0	-1.66525900	4.95729700	1.08126200
C	0	-1.01547500	4.27810600	2.26424000
N	0	0.26820500	3.92636100	2.08203300
O	0	-1.63934300	4.05787900	3.32550200
H	0	0.72919500	3.35381400	2.77819600
H	0	0.69117100	4.02704600	1.15394900
C	-1	6.80767600	6.38291400	-1.67026900
C	0	6.86947200	5.02335100	-2.37327000
C	0	5.65377200	4.14340500	-2.06892600
C	0	5.68739300	2.79438700	-2.79844600
C	0	4.65625300	1.79897800	-2.28359700
N	0	3.25483700	2.15714600	-2.68342200
H	0	3.06321800	1.83833200	-3.65423200
H	0	3.01240200	3.17294200	-2.59471500
H	0	2.58738000	1.65618200	-2.09106100
C	-1	-5.39438900	-1.86856200	-7.45444200
C	0	-4.73306300	-1.50383700	-6.13436000
C	0	-3.65345100	-2.51959900	-5.73316000
C	0	-2.99961700	-2.06357500	-4.45121500
N	0	-3.21670300	-2.80979400	-3.35565100
O	0	-2.37147500	-0.98913300	-4.41203100
H	0	-2.71226400	-2.55302500	-2.50812500
H	0	-3.52766700	-3.76695800	-3.45137700
C	-1	-8.50675700	3.20316600	-0.47756600
C	0	-7.55184000	4.23861300	0.13770600

C	0	-6.25884200	3.60807900	0.59925900
C	0	-5.29163700	3.22382500	-0.33926400
C	0	-6.00534400	3.33399300	1.94907000
C	0	-4.12893200	2.56418800	0.04548600
C	0	-4.83755600	2.68247300	2.35398300
C	0	-3.91093900	2.27511100	1.39218800
O	0	-2.76626200	1.58447600	1.71038000
H	0	-2.82965700	1.26714200	2.62768400
C	-1	-11.15204900	-0.70697200	-1.68727500
C	0	-9.63819000	-0.93090200	-1.78910800
C	0	-8.93458400	-0.75602000	-0.43833700
C	0	-7.46233400	-1.15870900	-0.41064200
N	0	-6.62819900	-0.28559800	-1.24630100
C	0	-5.31307900	-0.46041200	-1.41818100
N	0	-4.68000600	-1.47352600	-0.81813200
N	0	-4.61836700	0.31985400	-2.26947400
H	0	-7.04690200	0.54695400	-1.64570400
H	0	-3.65878400	-1.54125400	-0.88179500
H	0	-5.13369100	-1.98498900	-0.07478500
H	0	-3.60671400	0.36881000	-2.09412400
H	0	-5.04231500	1.19927300	-2.54095600
H	0	-11.37695800	0.30333600	-1.32383700
H	0	-11.63793600	-0.82889900	-2.66174500
H	0	-11.60745000	-1.42110400	-0.99059900
H	0	-0.81294600	-1.32222700	3.11033000
H	0	-1.31396800	-0.33331100	1.90922600
H	0	0.39568200	1.60480900	-0.40973000
H	0	0.62058900	-1.45807800	-0.29100300
H	0	-9.42817800	3.67647300	-0.83631400
H	0	-8.03583100	2.69023800	-1.32488200
H	0	-8.77940900	2.44446800	0.26425700
H	0	-7.33770000	5.01548600	-0.60723500
H	0	-8.04554900	4.73374900	0.98192500
H	0	-5.44731800	3.44309700	-1.39210300
H	0	-3.39064900	2.26045000	-0.68127200
H	0	-4.65301500	2.47163400	3.40323800
H	0	-6.73224500	3.63170300	2.70085000
H	0	-2.98601500	2.38834200	7.41690100
H	0	-3.63684800	2.67443100	5.78450000
H	0	-3.99099600	1.15492200	6.63103800
H	0	-2.43096600	-0.16841200	8.01036300
H	0	-1.02265100	-1.20845000	7.68876100
H	0	-0.80087700	0.35901500	8.49272800
H	0	-1.92932800	4.16229400	0.37213400
H	0	-0.92577900	5.57597800	0.56565700
H	0	-2.63791800	6.58848500	2.10523700
H	0	-3.63435700	5.14684400	1.93802000
H	0	-4.42634000	6.91084000	0.34657800
H	0	-2.81296900	6.99621000	-0.38123400
H	0	-3.78291100	5.52407700	-0.54183400
H	0	1.92925000	8.25730700	-1.47159800
H	0	0.48881300	7.22837400	-1.40618900
H	0	0.61219700	8.64178100	-0.34816500
H	0	2.49135500	7.58425600	0.90847600
H	0	1.08644200	6.52880900	0.93587600
H	0	3.11927400	5.21277600	0.49179700
H	0	3.54278000	6.24692400	-0.88003300
H	0	-4.24780300	5.97808700	-2.64620600
H	0	-3.28040300	7.16137600	-3.54392800
H	0	-3.89655200	5.71249600	-4.35837300
H	0	-1.73714400	5.78248000	-2.15203100
H	0	-1.45111400	5.46788500	-3.84973900
H	0	-0.32887000	-8.09208400	-6.07433800
H	0	-1.34187000	-8.24341000	-7.52395900
H	0	-0.22465900	-6.87678200	-7.35824100
H	0	-2.70479300	-7.38206300	-5.60190600
H	0	-2.59493900	-6.17579400	-6.88241900
H	0	-2.07391200	-6.77449000	-3.37483800
H	0	-0.61166900	-4.45072300	-6.67452400
H	0	0.40321800	-2.83890200	-5.09189700
H	0	-1.03505300	-5.16603600	-1.78632700
H	0	-5.90851900	-3.23181600	6.46454300

H	0	-4.85641800	-4.35001400	5.58044300
H	0	-4.66972700	-4.15716900	7.33522400
H	0	-3.91759500	-1.78720500	7.00347400
H	0	-2.86177400	-2.89454700	6.12326000
H	0	-2.66277900	-2.76876000	3.73333500
H	0	-5.80223300	-0.61750700	5.71952200
H	0	-6.44940700	0.42855700	3.56315300
H	0	-5.20864500	-0.12568400	1.49530200
H	0	-3.29469700	-1.72237200	1.58296900
H	0	0.36649800	-5.83135500	4.89346500
H	0	1.51505500	-5.80911600	6.24372700
H	0	0.14495100	-4.68425300	6.22215800
H	0	2.53646000	-4.64179600	4.27311000
H	0	2.26018200	-3.50146700	5.58309900
H	0	11.76090700	2.18461700	2.01791400
H	0	12.43273900	0.97875100	0.90747900
H	0	13.27808700	2.51910200	1.15856400
H	0	11.39004800	3.75565800	0.08127200
H	0	12.06460600	2.55654700	-1.02109200
H	0	9.08980300	3.60534100	0.82644100
H	0	6.85998000	2.54206200	0.62064400
H	0	6.61646900	0.38007300	-0.58132100
H	0	8.61869500	-0.70093400	-1.58814300
H	0	10.84442200	0.36192400	-1.37287700
H	0	10.47169900	-3.90721500	0.75972800
H	0	10.55084400	-3.38522900	-0.93148500
H	0	10.19194300	-2.20913500	0.33715700
H	0	8.08624700	-2.83462700	-0.87267100
H	0	8.40736300	-4.53959700	-0.59467500
H	0	8.14226400	-4.21133200	1.85115900
H	0	5.65361800	-3.92650200	1.79808000
H	0	5.76461700	-3.24561900	0.16676500
H	0	6.20860300	-4.93788800	0.45112600
H	0	7.13565200	-2.11178300	2.73731400
H	0	8.73501200	-1.78441200	2.05441300
H	0	7.28108100	-1.35310500	1.14397100
H	0	2.78110000	-2.53655300	-3.94605900
H	0	3.22057200	-2.04495800	-5.59782600
H	0	0.54675200	1.24096900	-2.93963200
H	0	0.24088000	1.05031500	-4.67294700
H	0	-0.70253900	0.11632900	-3.49863700
H	0	-7.09910900	-1.12315300	0.62274300
H	0	-7.35240800	-2.18997900	-0.76662200
H	0	-9.03487600	0.28074800	-0.09405700
H	0	-9.43633000	-1.38130500	0.31054600
H	0	-9.44076600	-1.94328500	-2.16684400
H	0	-9.21327300	-0.23576400	-2.52524400
H	0	4.83305300	0.79485900	-2.67463500
H	0	4.67546100	1.75407800	-1.19456800
H	0	6.66807000	2.32673700	-2.65485900
H	0	5.56153000	2.93538900	-3.88007100
H	0	5.60555700	3.95947100	-0.98568300
H	0	4.73711000	4.68288600	-2.33125500
H	0	6.94736800	5.17424500	-3.45897200
H	0	7.78155200	4.49163900	-2.06774600
H	0	6.75296700	6.25903700	-0.58142700
H	0	7.69183500	6.99089800	-1.89411700
H	0	5.92222200	6.94941400	-1.98438400
H	0	-4.08498900	-3.52036900	-5.62625500
H	0	-2.88259700	-2.56623300	-6.51066700
H	0	-4.27001400	-0.51300200	-6.20335300
H	0	-5.48424100	-1.44587200	-5.33581100
H	0	-6.15995300	-1.13517000	-7.73311000
H	0	-4.65702800	-1.90835000	-8.26560800
H	0	-5.87838300	-2.85130300	-7.39756800
H	0	2.39376600	-8.21192800	2.70494700
H	0	4.33941000	-7.81318500	1.11002500
H	0	4.21951700	-1.52185200	-4.23943400

***Pb*PAL_L205F_(*E*)-3-(pyridin-2-yl)acrylic acid_pro-(*S*)**

C	0	-0.20559800	-0.16380800	-0.54442300
N	0	-2.56878300	-1.38538500	0.45953500
O	0	2.02744400	-0.38714300	-1.19907000
C	0	-2.51554700	-0.04044500	0.32870400
C	0	-3.53948000	0.79019300	0.80752300
O	0	1.20753500	1.69876900	-1.11350500
C	0	-4.60695700	0.21969200	1.49789300
C	0	-4.63453200	-1.16300600	1.67442000
C	0	-3.60232400	-1.92089400	1.11450700
C	0	-1.31266200	0.54155600	-0.27058000
C	0	1.08408100	0.43210500	-0.98752900
H	0	-3.47962700	1.86353000	0.65622200
H	0	-5.39957900	0.84458300	1.89434500
H	0	-5.44139600	-1.64834200	2.21166000
H	0	-3.60592300	-3.00644300	1.19132000
C	-1	0.88322200	7.34363000	-7.01780800
C	0	1.96479200	6.54625500	-6.27374900
C	0	1.35587700	5.57559400	-5.29044500
C	0	0.67155300	4.44351600	-5.75437900
C	0	1.38248100	5.80897700	-3.91002800
C	0	0.03330100	3.57411200	-4.87533000
C	0	0.74372700	4.94726700	-3.01484200
C	0	0.07458600	3.82272400	-3.50052600
O	0	-0.56905200	2.94808000	-2.64463300
H	0	0.07762900	2.55899900	-1.98391200
C	-1	-3.13702300	1.52692100	-4.64013100
C	0	-2.33471800	0.27485200	-4.43269500
O	0	-2.75182600	-0.84734900	-4.81412500
N	0	-1.14545400	0.44610500	-3.85474300
C	-1	-0.19617400	-0.62564900	-3.68298900
H	0	-0.91681200	1.36447600	-3.46868200
C	-1	-10.07613600	3.04369200	-0.24562400
C	0	-8.56858000	3.29917900	-0.34925100
C	0	-7.81252300	3.30492800	0.99225000
C	0	-7.99353700	1.99626700	1.77066800
C	0	-6.32257500	3.59451200	0.76060000
C	-1	-12.25575100	-2.26299500	0.93961100
C	0	-11.45669100	-2.88576600	-0.21667800
C	0	-10.10168500	-2.23929500	-0.39612300
C	0	-8.95339200	-2.79307700	0.18657900
C	0	-9.97056500	-1.04427000	-1.11852400
C	0	-7.71027800	-2.16786600	0.06057600
C	0	-8.72983000	-0.41963800	-1.25433200
C	0	-7.59389200	-0.97643700	-0.65936400
C	-1	3.15169300	-1.61660100	6.58991700
C	0	0.35206900	-0.57222000	4.40731800
C	-1	1.23898400	0.47786200	7.76295700
C	0	-0.15035500	-0.29895000	3.03051400
C	0	1.96572900	-1.02842200	5.91937400
N	0	0.53882600	0.82674600	2.34598400
C	0	0.06329000	0.18674100	5.54543100
N	0	1.53319400	-1.28414400	4.68159200
O	0	-0.85815900	1.00355500	5.84777700
N	0	1.11695500	-0.13585100	6.46187600
H	0	1.80046700	-2.14067200	4.17555000
H	0	-1.21489900	-0.05181000	3.11081200
H	0	-0.07708400	-1.17537400	2.38386900
C	-1	-4.52708700	7.45321600	1.84661500
C	0	-3.12134400	7.25599500	2.41490200
C	0	-2.22841800	6.28743500	1.66234100
C	0	-2.72243300	5.35765800	0.73742400
C	0	-0.84769800	6.29639500	1.91659600
C	0	-1.86394000	4.45666500	0.10035900
C	0	0.00818700	5.38504300	1.30027800
C	0	-0.49891000	4.45392500	0.39133300
H	0	-5.11239000	6.52733200	1.88043900
H	0	-3.20219100	6.91132700	3.45629500
H	0	-5.06730400	8.20744500	2.42854400
H	0	-3.78267000	5.33106100	0.50593400
H	0	-0.44480000	7.02538600	2.61535100
H	0	-2.26606500	3.75175900	-0.62110500

H	0	1.06950500	5.39733300	1.53272900
H	0	0.15929600	3.72465900	-0.06694900
C	-1	-1.09751400	5.35949800	5.42737700
C	0	-1.87256000	4.26318800	4.71202700
C	0	-0.97128400	3.40379200	3.84678000
N	0	-1.58119200	2.66740900	2.90538000
O	0	0.25964500	3.35342300	4.04255800
H	0	-1.00137300	2.08225900	2.29634700
H	0	-2.54564100	2.84090500	2.66069200
C	-1	4.69956900	3.95629900	6.38235600
C	0	3.58855600	3.01248800	5.89057000
C	0	4.00589100	2.24754300	4.65509900
C	0	5.00850300	1.26940700	4.74502500
C	0	3.45455400	2.52506700	3.39737900
C	0	5.45807100	0.60084500	3.60853600
C	0	3.88922200	1.84329300	2.25578100
C	0	4.89732400	0.88482000	2.35998000
C	-1	-0.99757400	-7.84519600	-0.54143900
C	0	-1.57760300	-7.00338500	0.59835000
C	0	-2.49150400	-5.86415000	0.11083100
C	0	-1.77106200	-4.92081800	-0.85200900
O	0	-0.85362300	-4.17939000	-0.37805100
O	0	-2.10590400	-4.93277300	-2.07700300
C	-1	3.67698400	-6.10243500	-3.13794600
C	0	2.35854100	-5.39930000	-2.79179400
C	0	2.49830000	-3.89523100	-2.60067800
N	0	1.47294300	-3.25577400	-2.01384300
O	0	3.51083600	-3.28610200	-3.00775300
H	0	1.57264100	-2.25845600	-1.81036500
H	0	0.67713900	-3.74111600	-1.59290000
C	-1	3.65131600	-6.22893300	0.42721300
C	0	3.04414100	-5.63722700	1.69365700
C	0	1.79603200	-4.83401400	1.34957800
C	0	1.16171000	-4.13294100	2.52859100
N	0	-0.13700100	-3.82240100	2.38285000
O	0	1.81612800	-3.85805600	3.55900800
H	0	-0.58076600	-3.23696500	3.08066900
H	0	-0.58534000	-3.95661700	1.46779800
C	-1	-6.63476100	-6.55867700	-1.55902700
C	0	-6.66133600	-5.24202900	-2.33944800
C	0	-5.49208700	-4.32149400	-1.98059500
C	0	-5.47645000	-3.02283700	-2.79642900
C	0	-4.52611800	-1.97043400	-2.24333800
N	0	-3.07863700	-2.32653300	-2.43275100
H	0	-2.76253100	-1.97385800	-3.35764600
H	0	-2.86238800	-3.34715700	-2.36557100
H	0	-2.50776600	-1.87967200	-1.70163400
C	-1	5.49283000	1.76359300	-7.39809100
C	0	4.92267800	1.32461600	-6.05202900
C	0	3.75314500	2.20609400	-5.59556600
C	0	3.14406600	1.68676200	-4.30787200
N	0	2.96791800	2.57575800	-3.31544300
O	0	2.85433400	0.48090600	-4.18431000
H	0	2.43009100	2.28872800	-2.49215000
H	0	3.02761000	3.56561900	-3.51636800
C	-1	8.58523200	-3.01098000	-0.20598200
C	0	7.69906400	-4.03335800	0.52434900
C	0	6.36487200	-3.43749400	0.90678400
C	0	5.41981900	-3.13883600	-0.08396300
C	0	6.05886300	-3.09239700	2.22856500
C	0	4.23756700	-2.47137200	0.21832700
C	0	4.86326600	-2.44781900	2.55450200
C	0	3.96844900	-2.10828600	1.53816300
O	0	2.80646100	-1.41494400	1.77842500
H	0	2.84115300	-1.04028100	2.67547500
C	-1	11.16884600	0.91432800	-1.49710200
C	0	9.68652200	1.15990000	-1.76349600
C	0	8.85230200	1.05675900	-0.48320600
C	0	7.35003000	1.19077600	-0.70545300
N	0	6.87190100	0.08918300	-1.54156900
C	0	5.59312100	-0.15336800	-1.84332900
N	0	4.61648600	0.58994700	-1.33132100

N	0	5.31132100	-1.11731700	-2.74617400
H	0	7.56258400	-0.52897700	-1.94754400
H	0	3.63022800	0.28529800	-1.40851100
H	0	4.83105600	1.26491300	-0.61049200
H	0	4.38530500	-1.54901400	-2.73931800
H	0	6.05245800	-1.78497900	-2.92590800
H	0	11.33079800	-0.09009500	-1.08717500
H	0	11.76044900	1.00077100	-2.41552400
H	0	11.56634100	1.63640200	-0.77316400
H	0	0.89573100	1.47026100	3.05194400
H	0	1.34735400	0.46911300	1.84202500
H	0	-1.31860500	1.61827600	-0.40085200
H	0	-0.19055900	-1.23612900	-0.37932300
H	0	9.55688300	-3.44586600	-0.46823600
H	0	8.10683300	-2.67057200	-1.13122700
H	0	8.76286500	-2.13224300	0.42411500
H	0	7.54793200	-4.90461900	-0.12603000
H	0	8.21520600	-4.39143000	1.42253400
H	0	5.61675900	-3.41862200	-1.11462100
H	0	3.52697000	-2.21290100	-0.55344700
H	0	4.63561800	-2.18340800	3.58263800
H	0	6.76754300	-3.32429000	3.01985000
H	0	2.88383500	-2.05865200	7.55563400
H	0	3.58663000	-2.39174200	5.95317400
H	0	3.91302100	-0.84761500	6.76795800
H	0	2.29018500	0.62287900	8.01706900
H	0	0.73820700	1.44649200	7.72569300
H	0	0.76426200	-0.13469700	8.53649300
H	0	2.05020000	-4.05175300	0.62319900
H	0	1.05480000	-5.46707700	0.85405000
H	0	2.78823900	-6.43389400	2.40408500
H	0	3.77370200	-4.99121200	2.19040400
H	0	4.56879900	-6.78876700	0.64476300
H	0	2.95183100	-6.91240300	-0.06883400
H	0	3.90553800	-5.43571300	-0.28006300
H	0	-1.79431300	-8.27034500	-1.16394200
H	0	-0.35290900	-7.24202200	-1.19326800
H	0	-0.39424200	-8.67283200	-0.15192000
H	0	-2.15822400	-7.64639000	1.27127400
H	0	-0.76352800	-6.58062000	1.19820700
H	0	-2.83673400	-5.28290800	0.97501900
H	0	-3.37113800	-6.28301400	-0.38863300
H	0	4.43189900	-5.94367400	-2.36274700
H	0	3.51029800	-7.17949500	-3.23874600
H	0	4.08191000	-5.72884200	-4.08335500
H	0	1.90786100	-5.82588800	-1.88892200
H	0	1.62648000	-5.55535200	-3.59567000
H	0	0.27001600	7.91738200	-6.31337300
H	0	1.33130700	8.04565100	-7.73065400
H	0	0.21755600	6.67443300	-7.57423400
H	0	2.63464300	7.23518100	-5.74683800
H	0	2.57586100	6.00145000	-7.00474400
H	0	1.90098600	6.68471500	-3.52738300
H	0	0.63917500	4.23942400	-6.82182600
H	0	-0.47677400	2.69349400	-5.24820500
H	0	0.76491800	5.13898900	-1.94705300
H	0	5.60801500	3.39618200	6.62970200
H	0	4.95673400	4.69077400	5.61044000
H	0	4.38169000	4.50038900	7.27913400
H	0	3.34241500	2.30696800	6.69430200
H	0	2.67640700	3.58174700	5.67890800
H	0	2.66688100	3.26764100	3.31374400
H	0	5.44404000	1.04065500	5.71490800
H	0	6.23527000	-0.15230700	3.69255400
H	0	5.22850200	0.33757200	1.48552500
H	0	3.42816800	2.04916100	1.29206700
H	0	-0.60817700	6.02862300	4.71345100
H	0	-1.76764100	5.95938500	6.05223400
H	0	-0.32376700	4.93148600	6.07151800
H	0	-2.67996000	4.67737200	4.09858500
H	0	-2.34458600	3.59175300	5.44159100
H	0	-11.71702900	-2.37081500	1.88807500

H	0	-12.42153400	-1.19324300	0.76756200
H	0	-13.23309600	-2.74748700	1.04427700
H	0	-11.32781800	-3.95837700	-0.03072300
H	0	-12.03416400	-2.78776700	-1.14394800
H	0	-9.03582700	-3.72224600	0.74540000
H	0	-6.83311100	-2.61323800	0.51930600
H	0	-6.62920500	-0.48804100	-0.75674700
H	0	-8.64946400	0.50083900	-1.82466400
H	0	-10.84988000	-0.60440900	-1.58321000
H	0	-10.55056200	3.74969800	0.44773400
H	0	-10.55898000	3.16047200	-1.22283500
H	0	-10.29265100	2.02970000	0.10811500
H	0	-8.11520100	2.54034800	-1.00263300
H	0	-8.40151800	4.26632400	-0.84295400
H	0	-8.22587900	4.12146400	1.60378600
H	0	-5.77216100	3.64637600	1.70796900
H	0	-5.86288900	2.80513700	0.15145900
H	0	-6.17912800	4.54668700	0.23501300
H	0	-7.41844500	2.01592500	2.70443600
H	0	-9.04208000	1.81759300	2.03220200
H	0	-7.64790200	1.13853500	1.18149400
H	0	-2.76609300	2.36456100	-4.04670600
H	0	-3.09528800	1.80423100	-5.69999300
H	0	-0.42031500	-1.23890300	-2.80648200
H	0	-0.21279000	-1.27587500	-4.56257900
H	0	0.80189500	-0.20557700	-3.58017900
H	0	6.83532500	1.16857000	0.26004100
H	0	7.11449700	2.14677500	-1.18999800
H	0	9.05141100	0.09795000	0.01333700
H	0	9.15648700	1.84409100	0.21615700
H	0	9.53925500	2.15204500	-2.21034600
H	0	9.33734000	0.43259500	-2.50793400
H	0	-4.66492100	-1.00487900	-2.73289000
H	0	-4.68959400	-1.83933400	-1.17468100
H	0	-6.47612600	-2.57252100	-2.78212300
H	0	-5.23858800	-3.22781900	-3.84837500
H	0	-5.54973600	-4.06910700	-0.91204600
H	0	-4.54810900	-4.86041400	-2.11913900
H	0	-6.64256600	-5.45269000	-3.41778700
H	0	-7.60525800	-4.71462700	-2.14223500
H	0	-6.67429800	-6.37442300	-0.47803200
H	0	-7.48544300	-7.19865100	-1.82047100
H	0	-5.71563500	-7.12092400	-1.76604100
H	0	4.07157100	3.24792100	-5.48174500
H	0	2.96195800	2.19208800	-6.35731700
H	0	4.57513600	0.28737900	-6.11161000
H	0	5.70810700	1.34683900	-5.28490500
H	0	6.32574600	1.12029600	-7.70399200
H	0	4.72730300	1.71863400	-8.18247600
H	0	5.86431500	2.79481400	-7.35533200
H	0	-2.61039400	8.22567400	2.46876700
H	0	-4.49327700	7.79017800	0.80404800
H	0	-4.18510700	1.33674100	-4.39020300
O	0	-2.88905900	0.78176900	0.94102500

11. References

(1) Trujillo-Ferrara, J. G.; Padilla-Martínez, I. I.; Martínez-Martínez, F. J.; Höpfl, H.; Farfan-García, N.; García-Báez, E. V. The *E* and *Z* Isomers of 3-(Benzoxazol-2-Yl)Prop-2-Enoic Acid. *Acta Crystallogr C Cryst Struct Commun* **2004**, *60* (10), o723–o726.

(2) Ahmed, S. T.; Parmeggiani, F.; Weise, N. J.; Flitsch, S. L.; Turner, N. J. Synthesis of Enantiomerically Pure Ring-Substituted ʟ -Pyridylalanines by Biocatalytic Hydroamination. *Org. Lett.* **2016**, *18* (21), 5468–5471.

(3) Giordanetto, F.; Knerr, L.; Nordberg, P.; Pettersen, D.; Selmi, N.; Beisel, H.-G.; De La Motte, H.; Månsson, Å.; Dahlström, M.; Broddefalk, J.; Saarinen, G.; Klingegård, F.; Hurt-Camejo, E.; Rosengren, B.; Wikström, J.; Wågberg, M.; Brengdahl, J.; Rohman, M.; Sandmark, J.; Åkerud, T.; Roth, R. G.; Jansen, F.; Ahlqvist, M. Design of Selective $sPLA_2$ -X Inhibitor (−)-2-{2-[Carbamoyl-6-(Trifluoromethoxy)-1 *H* -Indol-1-Yl]Pyridine-2-Yl}propanoic Acid. *ACS Med. Chem. Lett.* **2018**, *9* (7), 600–605.

(4) Antihelmintic Depsipetide Compounds. WO2016/187534A, 2016.

(5) CrysAlisPro Software System, Rigaku Oxford Diffraction, 2024.

(6) Sheldrick, G. M. Crystal Structure Refinement with *SHELXL*. *Acta Crystallogr C Struct Chem* **2015**, *71* (1), 3–8.

(7) Dolomanov, O. V.; Bourhis, L. J.; Gildea, R. J.; Howard, J. A. K.; Puschmann, H. *OLEX2* : A Complete Structure Solution, Refinement and Analysis Program. *J Appl Crystallogr* **2009**, *42* (2), 339–341.

(8) Sheldrick, G. M. *SHELXT* – Integrated Space-Group and Crystal-Structure Determination. *Acta Crystallogr A Found Adv* **2015**, *71* (1), 3–8.

(9) Trott, O.; Olson, A. J. AutoDock Vina: Improving the Speed and Accuracy of Docking with a New Scoring Function, Efficient Optimization, and Multithreading. *Journal of Computational Chemistry* **2010**, *31* (2), 455–461.

(10) Eberhardt, J.; Santos-Martins, D.; Tillack, A. F.; Forli, S. AutoDock Vina 1.2.0: New Docking Methods, Expanded Force Field, and Python Bindings. *J. Chem. Inf. Model.* **2021**, *61* (8), 3891–3898.

(11) M. J. Frisch, G. W. T., H. B. Schlegel, G. E. Scuseria, M. A. Robb, J. R. Cheeseman, G. Scalmani, V. Barone, G. A. Petersson, H. Nakatsuji, X. Li, M. Caricato, A. Marenich, J. Bloino, B. G. Janesko, R. Gomperts, B. Mennucci, H. P. Hratchian, J. V. Ortiz, A. F. Izmaylov, J. L. Sonnenberg, D. Williams-Young, F. Ding, F. Lipparini, F. Egidi, J. Goings, B. Peng, A. Petrone, T. Henderson, D. Ranasinghe, V. G. Zakrzewski, J. Gao, N. Rega, G. Zheng, W. Liang, M. Hada, M. Ehara, K. Toyota, R. Fukuda, J. Hasegawa, M. Ishida, T. Nakajima, Y. Honda, O. Kitao, H. Nakai, T. Vreven, K. Throssell, J. A. Montgomery, Jr., J. E. Peralta, F. Ogliaro, M. Bearpark, J. J. Heyd, E. Brothers, K. N. Kudin, V. N. Staroverov, T. Keith, R. Kobayashi, J. Normand, K. Raghavachari, A. Rendell, J. C. Burant, S. S. Iyengar, J. Tomasi, M. Cossi, J. M. Millam, M. Klene, C. Adamo, R. Cammi, J. W. Ochterski, R. L. Martin, K. Morokuma, O. Farkas, J. B. Foresman, and D. J. Fox,. Gaussian 09, Revision D.01. Gaussian, Inc.: Wallingford CT, 2016., 2016.

(12) Marenich, A. V.; Cramer, C. J.; Truhlar, D. G. Universal Solvation Model Based on Solute Electron Density and on a Continuum Model of the Solvent Defined by the Bulk Dielectric Constant and Atomic Surface Tensions. *J. Phys. Chem. B* **2009**, *113* (18), 6378–6396.

(13) Grimme, S.; Antony, J.; Ehrlich, S.; Krieg, H. A Consistent and Accurate *Ab Initio* Parametrization of Density Functional Dispersion Correction (DFT-D) for the 94 Elements H-Pu. *The Journal of Chemical Physics* **2010**, *132* (15), 154104.

12. HPLC chromatograms

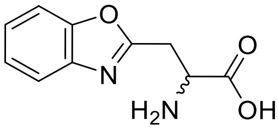

D/L-2a

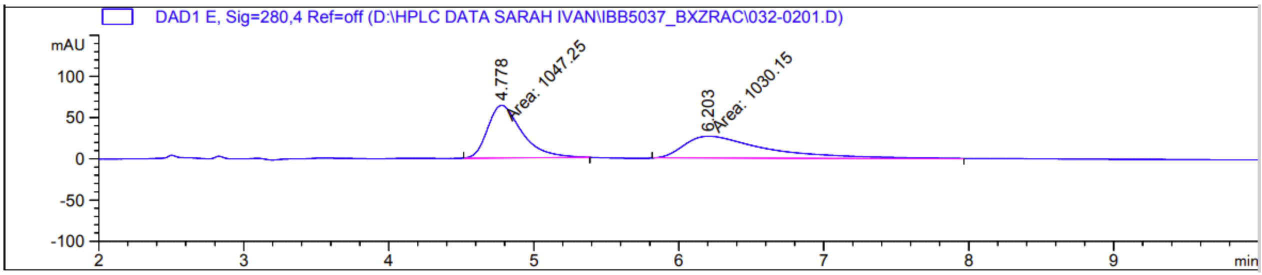

Signal 5: DAD1 E, Sig=280,4 Ref=off

Peak #	RetTime [min]	Type	Width [min]	Area [mAU*s]	Height [mAU]	Area %
1	4.778	MM	0.2730	1047.25391	63.93689	50.4116
2	6.203	MM	0.6498	1030.15137	26.42186	49.5884

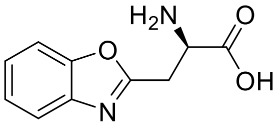

2a

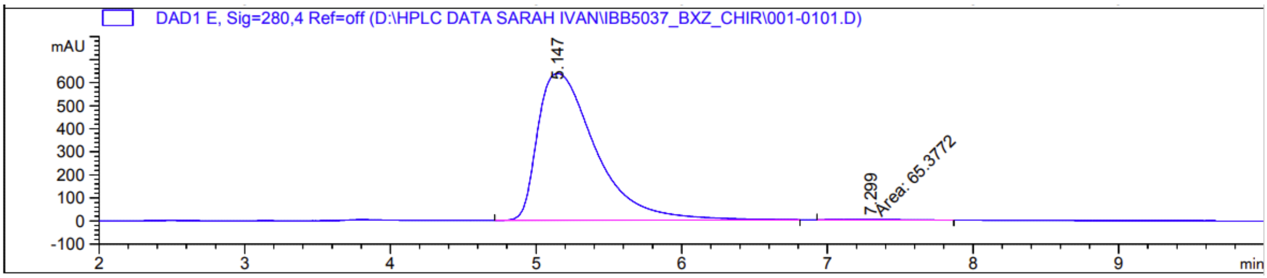

Signal 5: DAD1 E, Sig=280,4 Ref=off

Peak #	RetTime [min]	Type	Width [min]	Area [mAU*s]	Height [mAU]	Area %
1	5.147	BB	0.4225	1.76113e4	637.10968	99.6301
2	7.299	MM	0.5852	65.37724	1.86190	0.3699

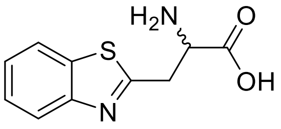

D/L-2b

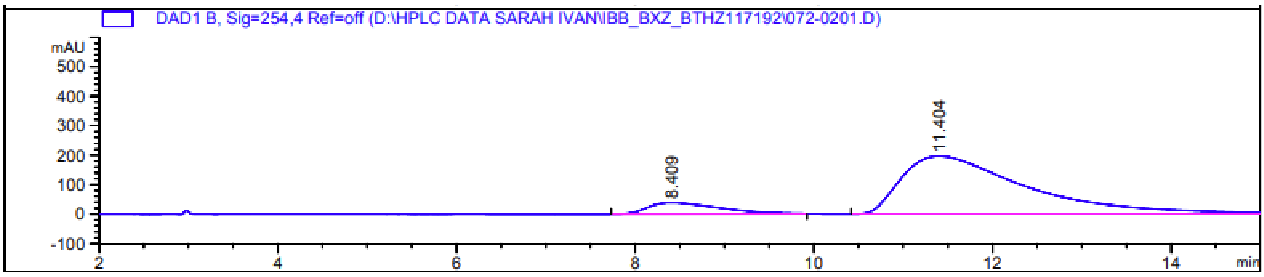

Signal 2: DAD1 B, Sig=254,4 Ref=off

Peak #	RetTime [min]	Type	Width [min]	Area [mAU*s]	Height [mAU]	Area %
1	8.409	BB	0.6555	2121.81714	38.70720	10.1773
2	11.404	BB	1.1289	1.87268e4	196.48413	89.8227

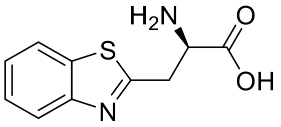

2b

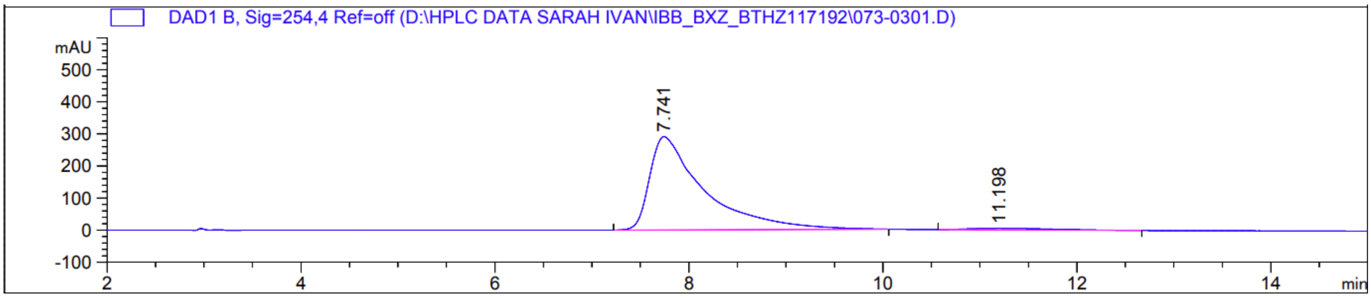

Signal 2: DAD1 B, Sig=254,4 Ref=off

Peak #	RetTime [min]	Type	Width [min]	Area [mAU*s]	Height [mAU]	Area %
1	7.741	BB	0.5578	1.13834e4	291.36334	97.2884
2	11.198	BB	0.7460	317.27911	5.00872	2.7116

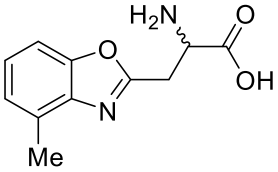

D/L-2c

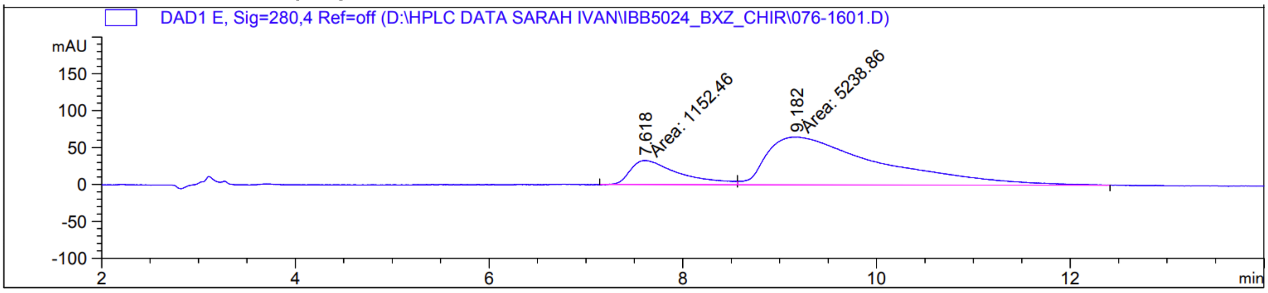

Signal 5: DAD1 E, Sig=280,4 Ref=off

Peak #	RetTime [min]	Type	Width [min]	Area [mAU*s]	Height [mAU]	Area %
1	7.618	MF	0.5904	1152.46289	32.53391	18.0317
2	9.182	FM	1.3480	5238.85693	64.77474	81.9683

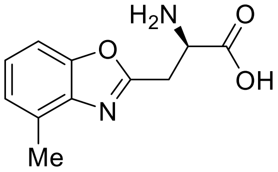

2c

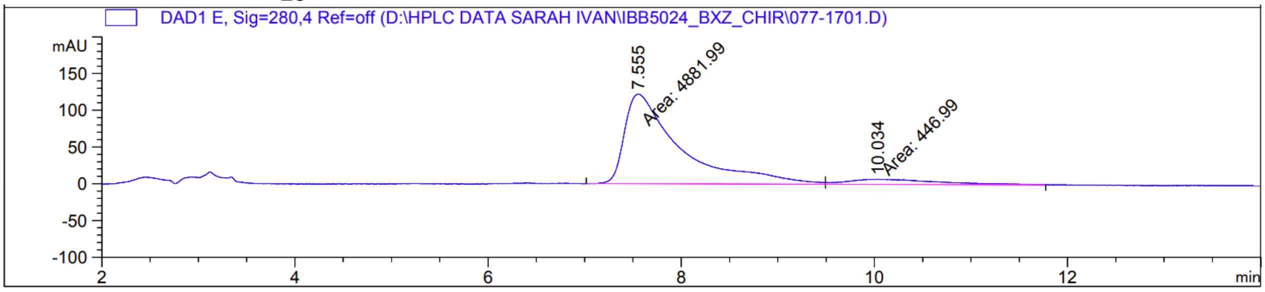

Signal 5: DAD1 E, Sig=280,4 Ref=off

Peak #	RetTime [min]	Type	Width [min]	Area [mAU*s]	Height [mAU]	Area %
1	7.555	MF	0.6674	4881.98828	121.92346	91.6121
2	10.034	FM	1.0889	446.99045	6.84187	8.3879

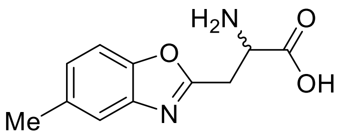

D/L-2d

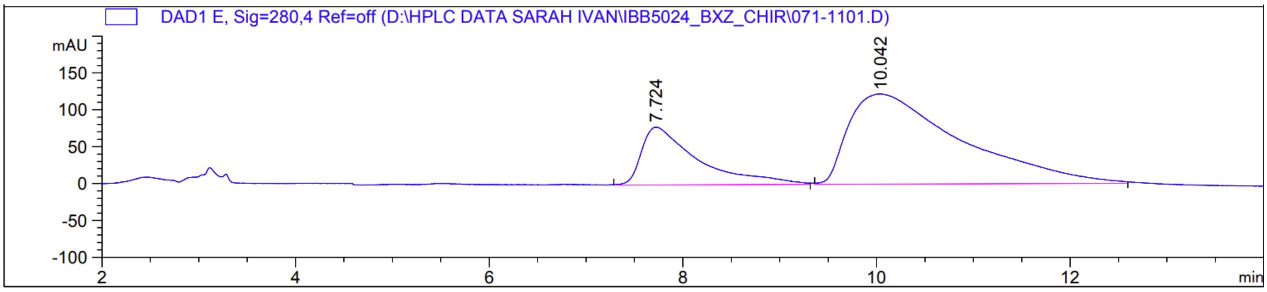

Signal 5: DAD1 E, Sig=280,4 Ref=off

Peak #	RetTime [min]	Type	Width [min]	Area [mAU*s]	Height [mAU]	Area %
1	7.724	VV	0.4800	3167.00732	78.28344	24.3759
2	10.042	BV	0.9412	9825.34863	122.18227	75.6241

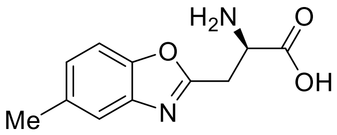

2d

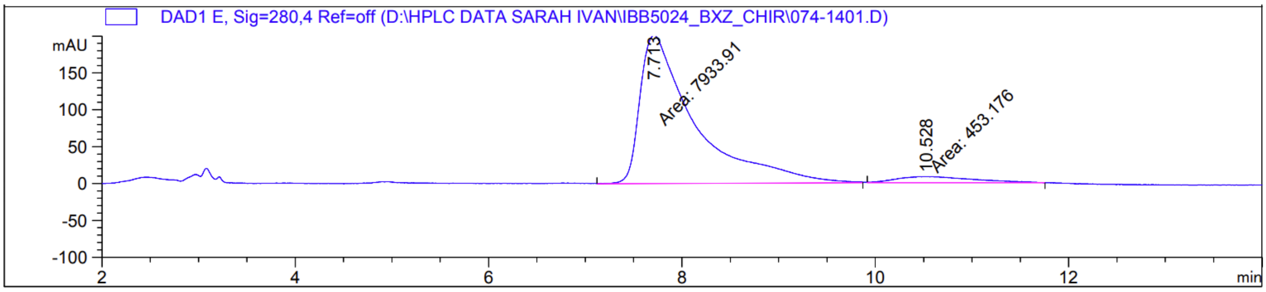

Signal 5: DAD1 E, Sig=280,4 Ref=off

Peak #	RetTime [min]	Type	Width [min]	Area [mAU*s]	Height [mAU]	Area %
1	7.713	MM	0.6581	7933.90918	200.94144	94.5967
2	10.528	MM	0.9161	453.17645	8.24499	5.4033

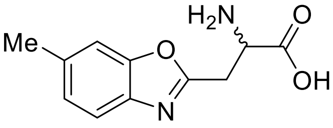

D/L-**2e**

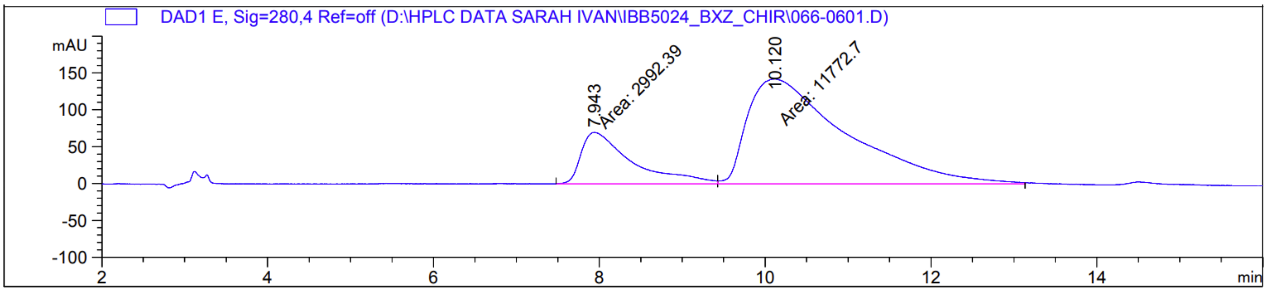

Signal 5: DAD1 E, Sig=280,4 Ref=off

Peak #	RetTime [min]	Type	Width [min]	Area [mAU*s]	Height [mAU]	Area %
1	7.943	MF	0.7142	2992.38696	69.82706	20.2667
2	10.120	FM	1.3781	1.17727e4	142.38095	79.7333

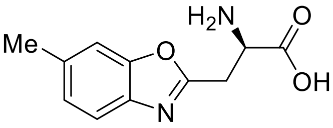

2e

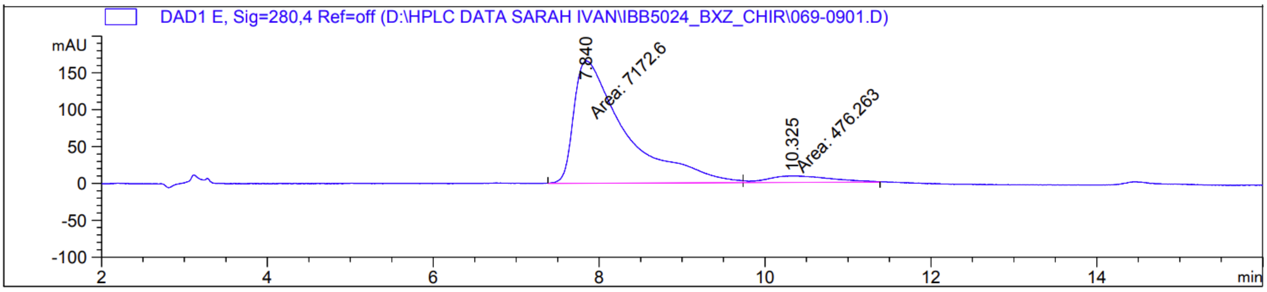

Signal 5: DAD1 E, Sig=280,4 Ref=off

Peak #	RetTime [min]	Type	Width [min]	Area [mAU*s]	Height [mAU]	Area %
1	7.840	MF	0.7202	7172.59912	165.98148	93.7734
2	10.325	FM	0.8994	476.26294	8.82589	6.2266

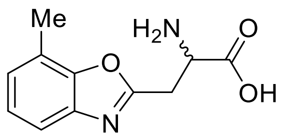

D/L-**2f**

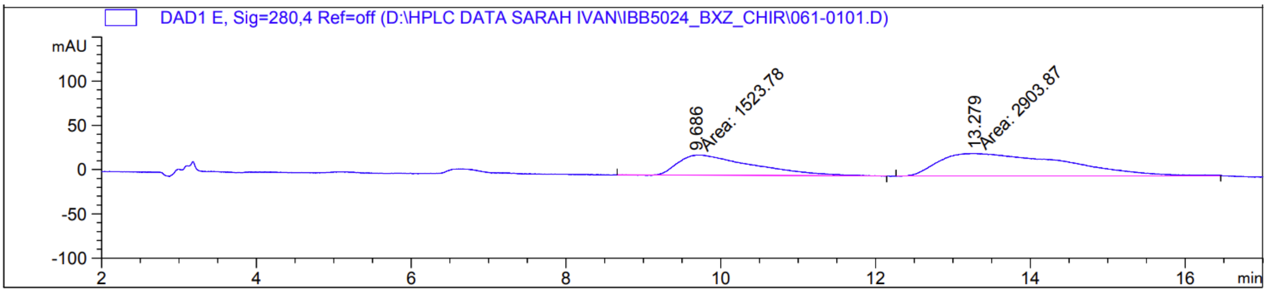

Signal 5: DAD1 E, Sig=280,4 Ref=off

Peak #	RetTime [min]	Type	Width [min]	Area [mAU*s]	Height [mAU]	Area %
1	9.686	MM	1.1145	1523.77637	22.78768	34.4150
2	13.279	MM	1.9106	2903.87085	25.33101	65.5850

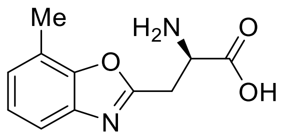

2f

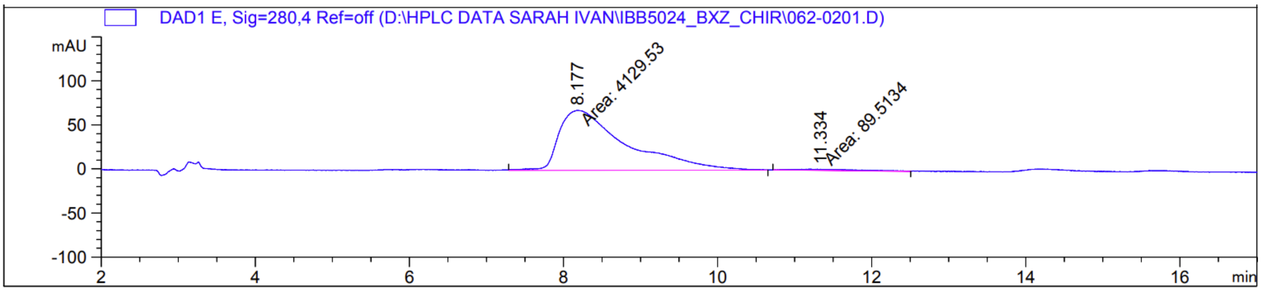

Signal 5: DAD1 E, Sig=280,4 Ref=off

Peak #	RetTime [min]	Type	Width [min]	Area [mAU*s]	Height [mAU]	Area %
1	8.177	MM	1.0106	4129.53418	68.10590	97.8784
2	11.334	MM	1.0539	89.51341	1.41556	2.1216

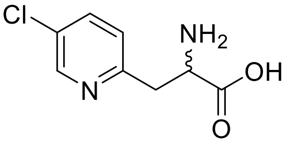

D/L-**2g**

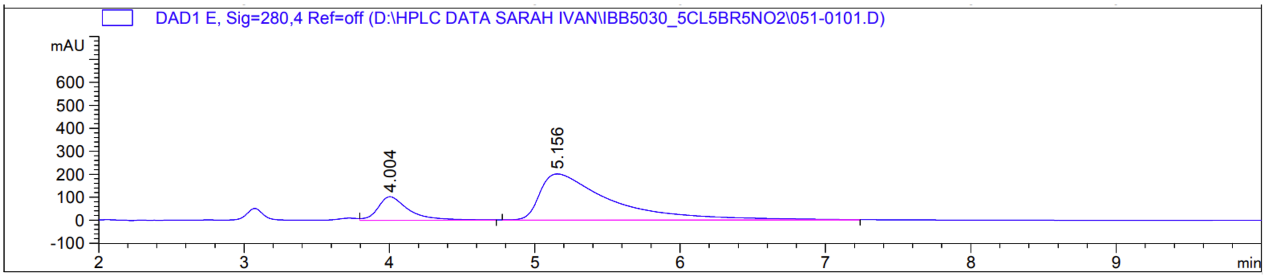

Signal 5: DAD1 E, Sig=280,4 Ref=off

Peak #	RetTime [min]	Type	Width [min]	Area [mAU*s]	Height [mAU]	Area %
1	4.004	VB	0.2188	1528.04419	102.54663	18.3218
2	5.156	BB	0.4813	6811.97070	201.04160	81.6782

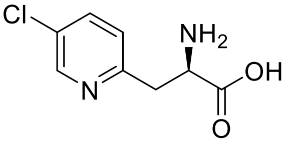

2g

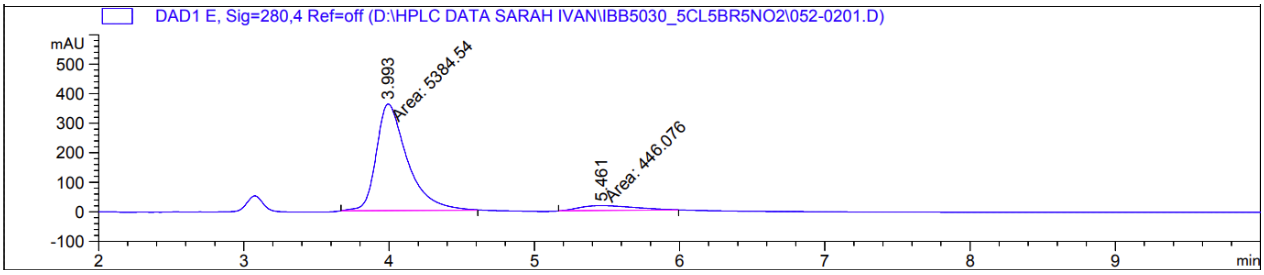

Signal 5: DAD1 E, Sig=280,4 Ref=off

Peak #	RetTime [min]	Type	Width [min]	Area [mAU*s]	Height [mAU]	Area %
1	3.993	MM	0.2482	5384.53516	361.56384	92.3494
2	5.461	MM	0.3145	446.07559	16.72076	7.6506

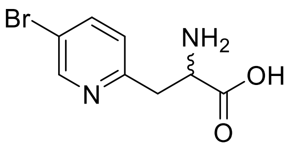

D/L-2h

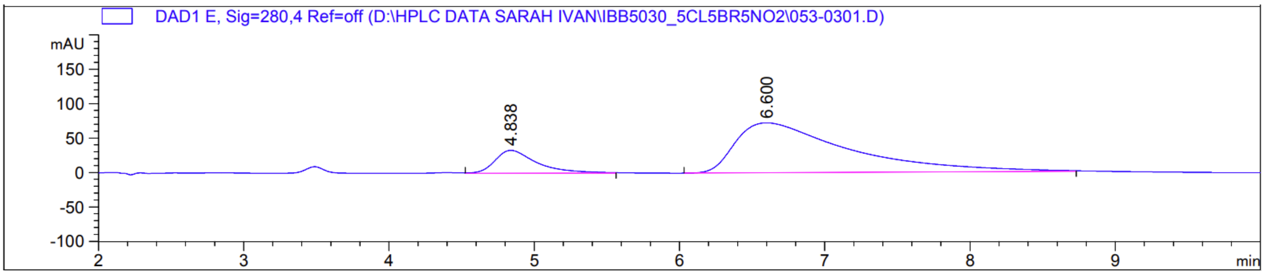

Signal 5: DAD1 E, Sig=280,4 Ref=off

Peak #	RetTime [min]	Type	Width [min]	Area [mAU*s]	Height [mAU]	Area %
1	4.838	VB	0.2932	658.76202	33.10035	14.3949
2	6.600	BB	0.7444	3917.59058	72.50579	85.6051

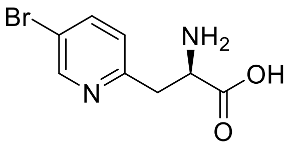

2h

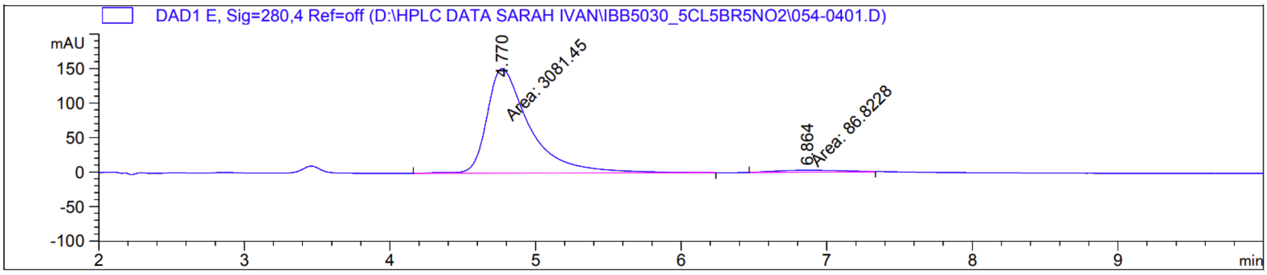

Signal 5: DAD1 E, Sig=280,4 Ref=off

Peak #	RetTime [min]	Type	Width [min]	Area [mAU*s]	Height [mAU]	Area %
1	4.770	MM	0.3390	3081.44775	151.49141	97.2596
2	6.864	MM	0.3762	86.82276	2.71051	2.7404

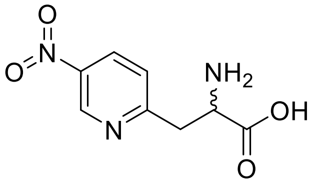

D/L-**2i**

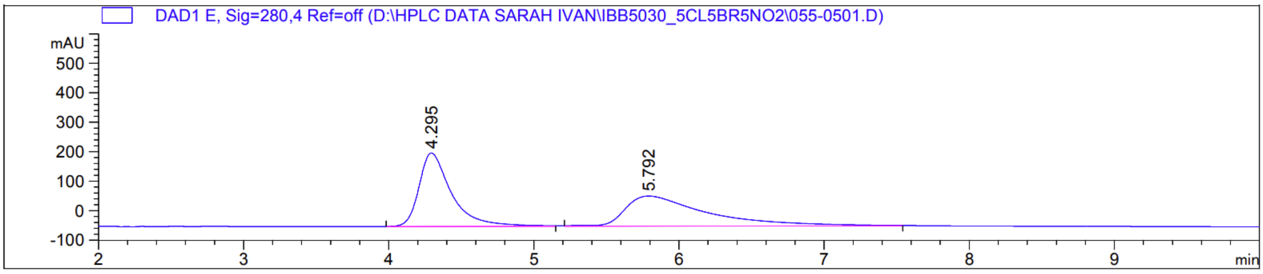

Signal 5: DAD1 E, Sig=280,4 Ref=off

Peak #	RetTime [min]	Type	Width [min]	Area [mAU*s]	Height [mAU]	Area %
1	4.295	BB	0.2288	3886.46777	249.47220	49.4132
2	5.792	BB	0.5327	3978.76782	102.08176	50.5868

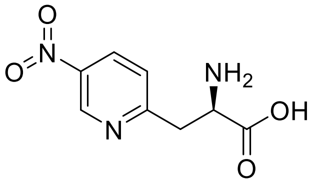

2i

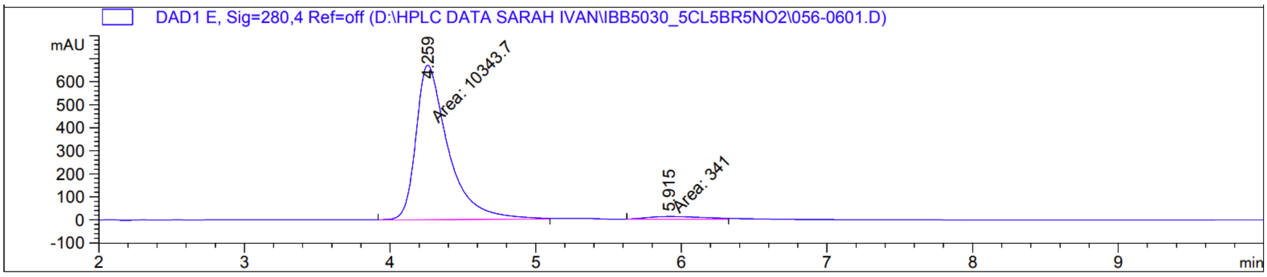

Signal 5: DAD1 E, Sig=280,4 Ref=off

Peak #	RetTime [min]	Type	Width [min]	Area [mAU*s]	Height [mAU]	Area %
1	4.259	MM	0.2567	1.03437e4	671.60339	96.8085
2	5.915	MM	0.4653	341.00049	12.21402	3.1915

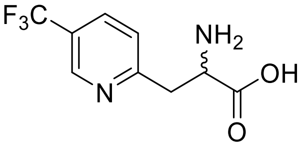

D/L-2j

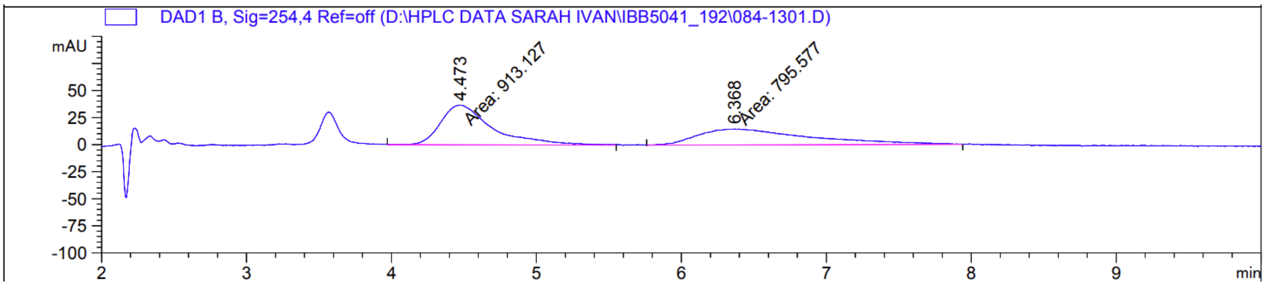

Signal 2: DAD1 B, Sig=254,4 Ref=off

Peak #	RetTime [min]	Type	Width [min]	Area [mAU*s]	Height [mAU]	Area %
1	4.473	MM	0.4140	913.12665	36.75885	53.4397
2	6.368	MM	0.9048	795.57666	14.65464	46.5603

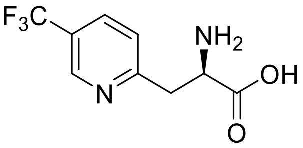

2j

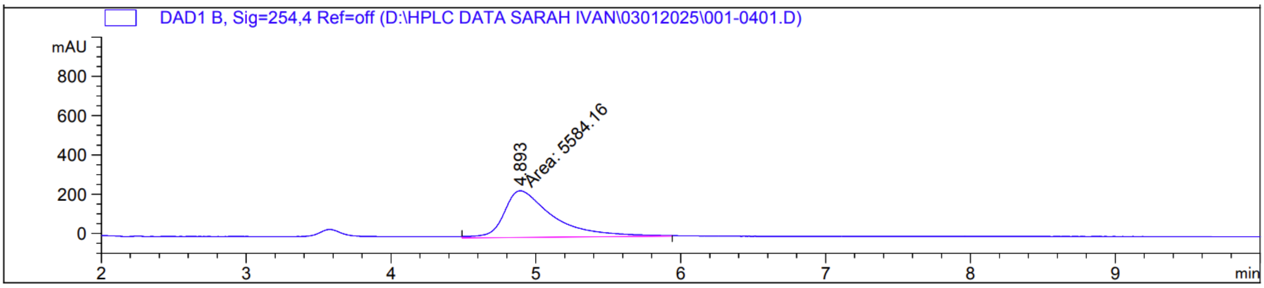

Signal 2: DAD1 B, Sig=254,4 Ref=off

Peak #	RetTime [min]	Type	Width [min]	Area [mAU*s]	Height [mAU]	Area %
1	4.893	MM	0.3923	5584.15869	237.25241	100.0000

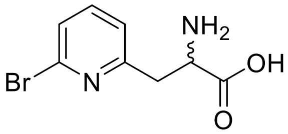

D/L-2k

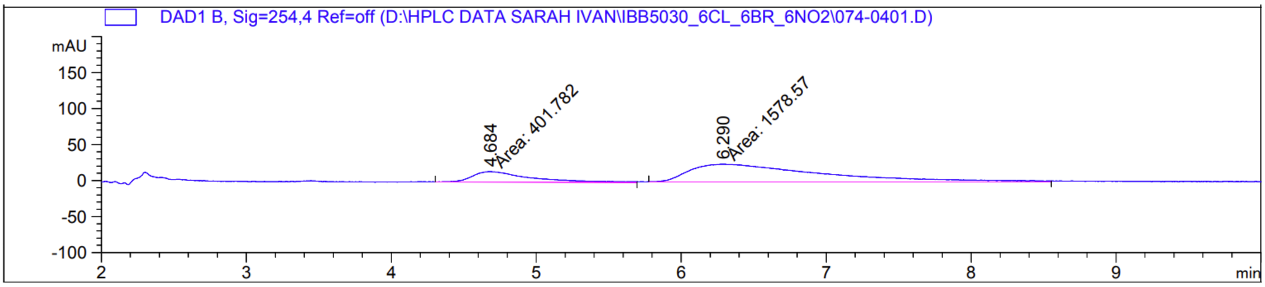

Signal 2: DAD1 B, Sig=254,4 Ref=off

Peak #	RetTime [min]	Type	Width [min]	Area [mAU*s]	Height [mAU]	Area %
1	4.684	MM	0.4592	401.78223	14.58412	20.2884
2	6.290	MM	1.0622	1578.56995	24.76907	79.7116

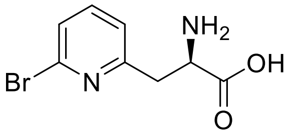

2k

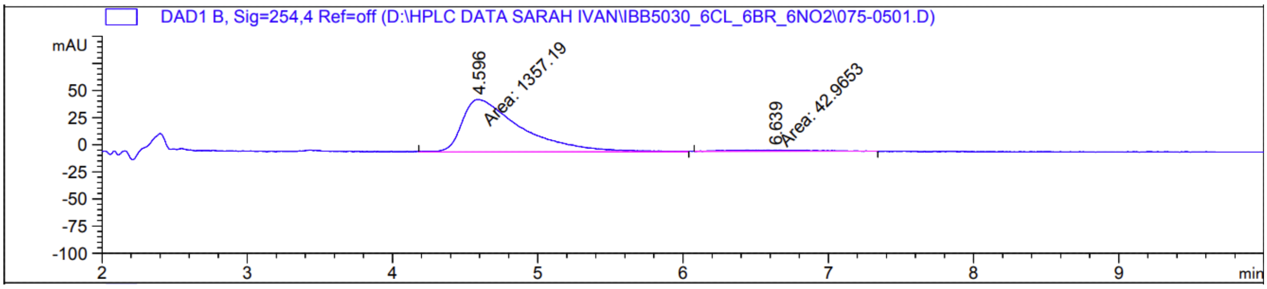

Signal 2: DAD1 B, Sig=254,4 Ref=off

Peak #	RetTime [min]	Type	Width [min]	Area [mAU*s]	Height [mAU]	Area %
1	4.596	MM	0.4689	1357.18896	48.24038	96.9314
2	6.639	MM	0.5922	42.96529	1.20917	3.0686

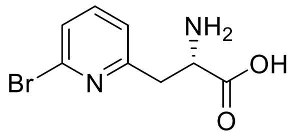

L-2k

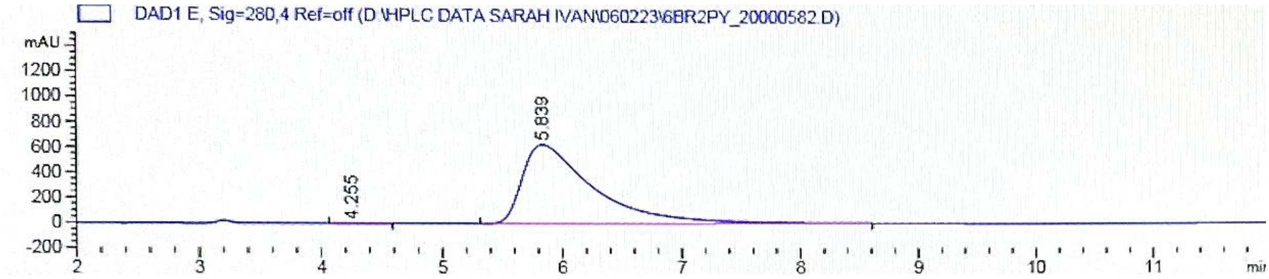

Signal 5: DAD1 E, Sig=280,4 Ref=off

Peak #	RetTime [min]	Type	Width [min]	Area [mAU*s]	Height [mAU]	Area %
1	4.255	BB	0.1837	38.21334	2.92044	0.1448
2	5.839	BB	0.6022	2.63503e4	632.26746	99.8552

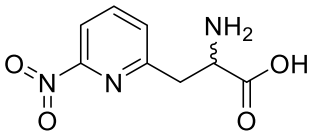

D/L-2l

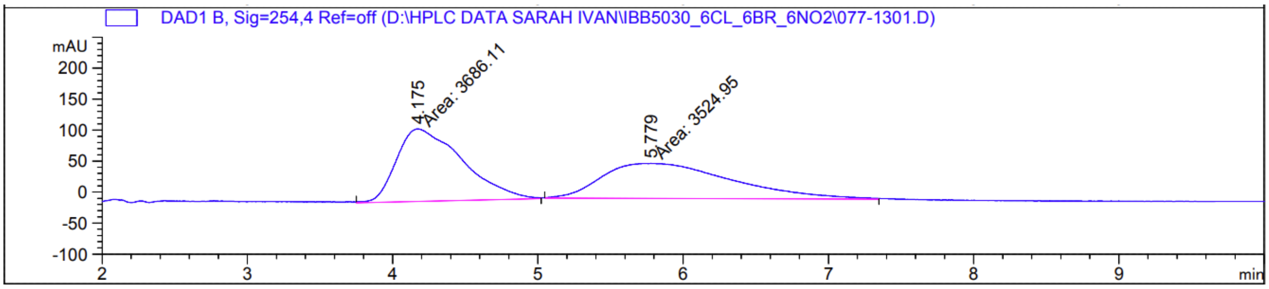

Signal 2: DAD1 B, Sig=254,4 Ref=off

Peak #	RetTime [min]	Type	Width [min]	Area [mAU*s]	Height [mAU]	Area %
1	4.175	MM	0.5249	3686.11450	117.03957	51.1175
2	5.779	MM	1.0368	3524.94995	56.66400	48.8825

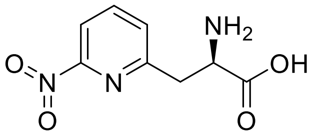

2l

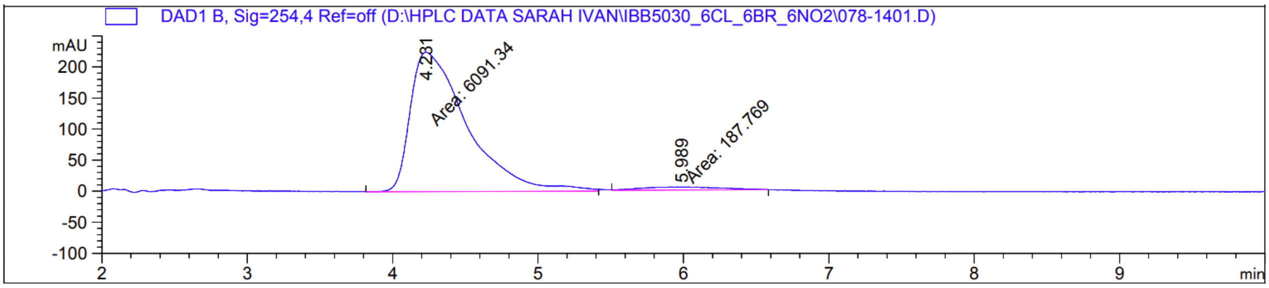

Signal 2: DAD1 B, Sig=254,4 Ref=off

Peak #	RetTime [min]	Type	Width [min]	Area [mAU*s]	Height [mAU]	Area %
1	4.231	MM	0.4526	6091.34424	224.30164	97.0096
2	5.989	MM	0.6294	187.76881	4.97209	2.9904

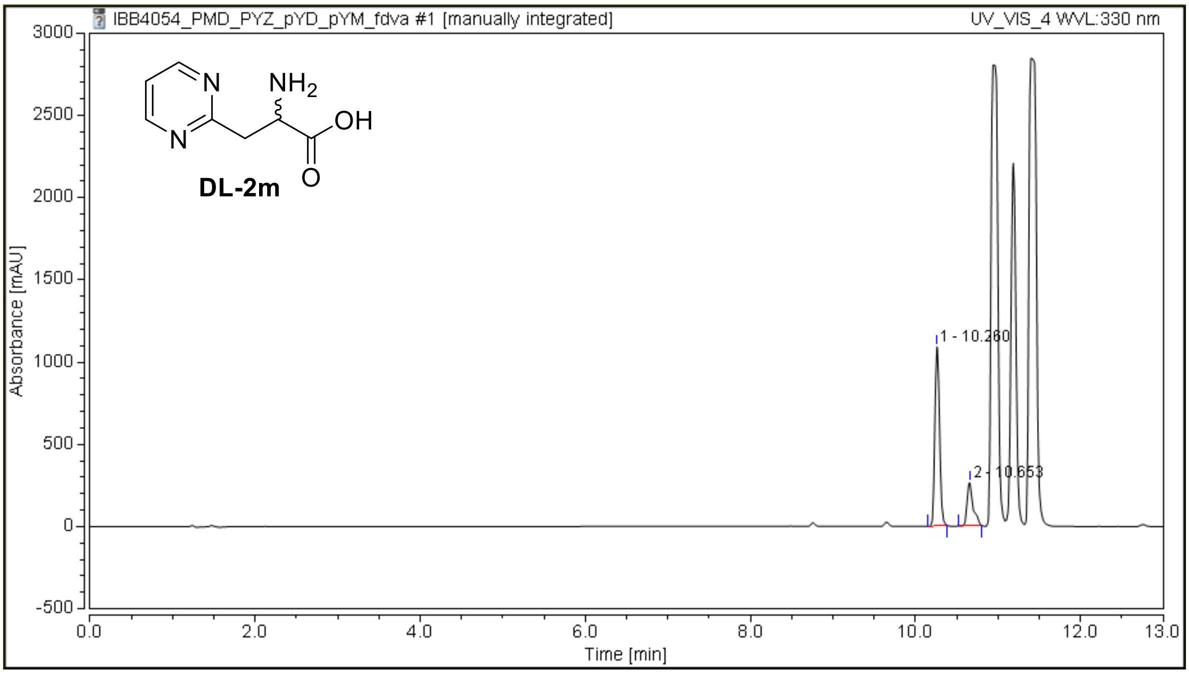

Integration Results

No.	Peak Name	Retention Time<br>min	Area<br>mAU*min	Height<br>mAU	Relative Area<br>%	Relative Height<br>%	Amount<br>n.a.
1		10.260	71.388	1089.269	76.48	80.54	n.a.
2		10.653	21.952	263.155	23.52	19.46	n.a.
**Total:**			**93.340**	**1352.424**	**100.00**	**100.00**	

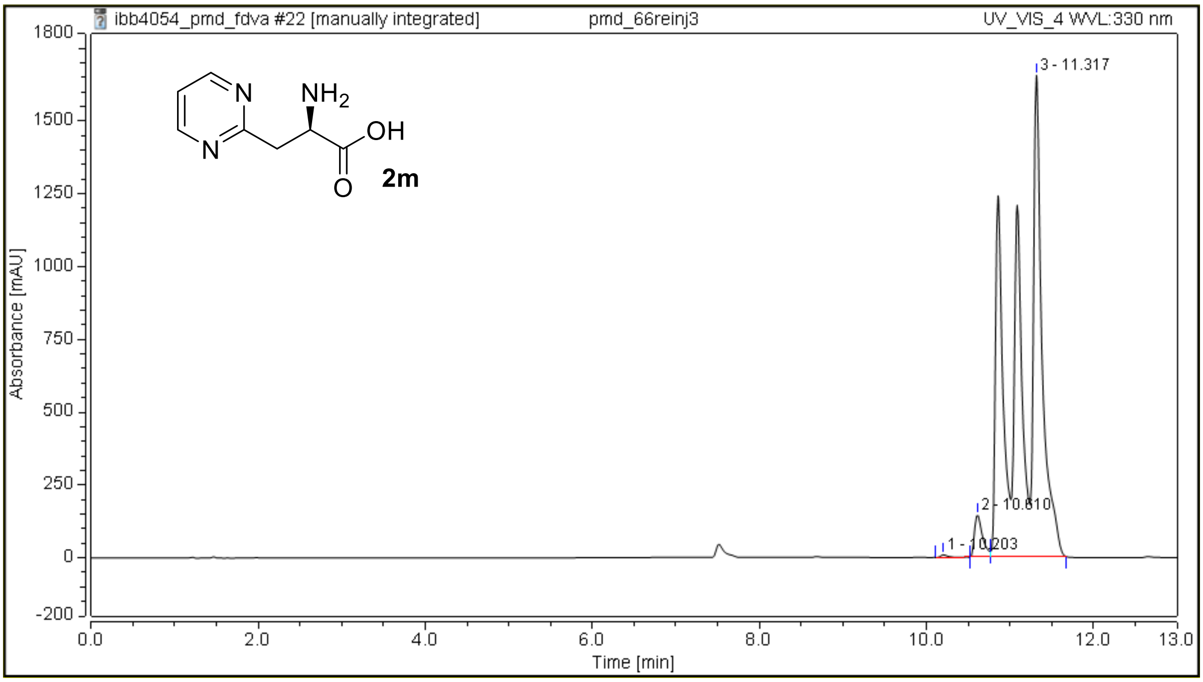

Integration Results

No.	Peak Name	Retention Time<br>min	Area<br>mAU*min	Height<br>mAU	Relative Area<br>%	Relative Height<br>%	Amount<br>n.a.
1		10.203	0.836	8.983	0.17	0.50	n.a.
2		10.610	16.205	145.682	3.38	8.05	n.a.
3		11.317	462.244	1654.315	96.44	91.45	n.a.
**Total:**			**479.286**	**1808.980**	**100.00**	**100.00**	

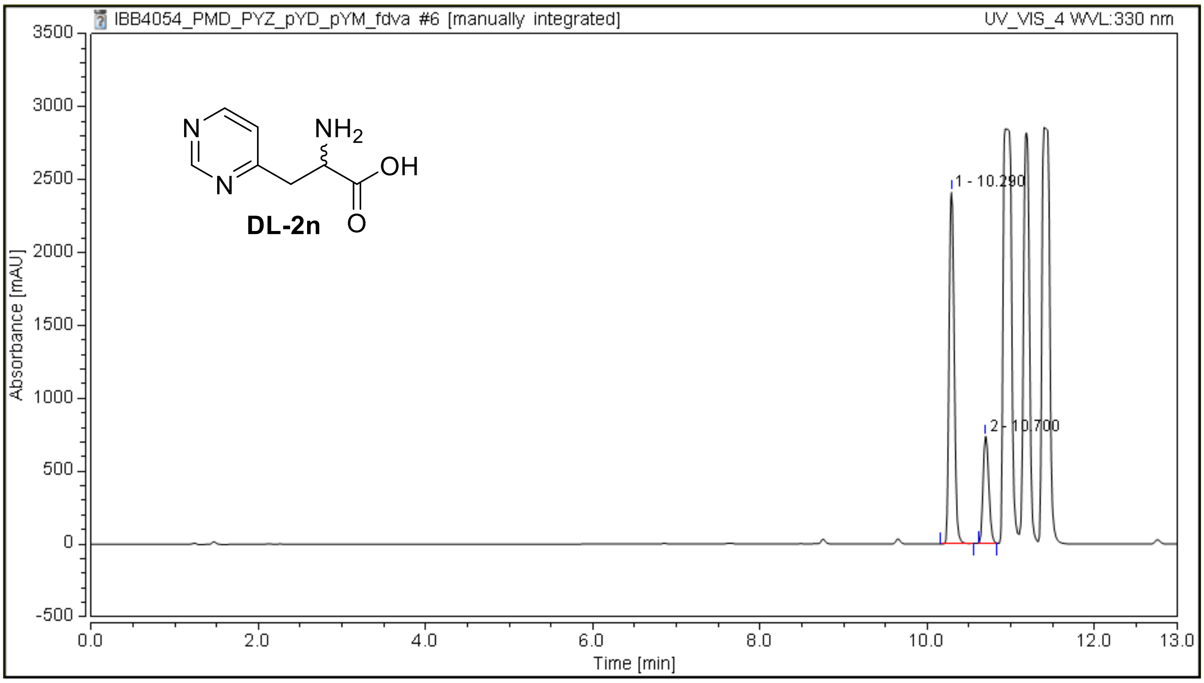

Integration Results							
No.	Peak Name	Retention Time min	Area mAU*min	Height mAU	Relative Area %	Relative Height %	Amount n.a.
1		10.290	171.664	2409.159	74.74	76.67	n.a.
2		10.700	58.005	733.142	25.26	23.33	n.a.
**Total:**			**229.669**	**3142.301**	**100.00**	**100.00**	

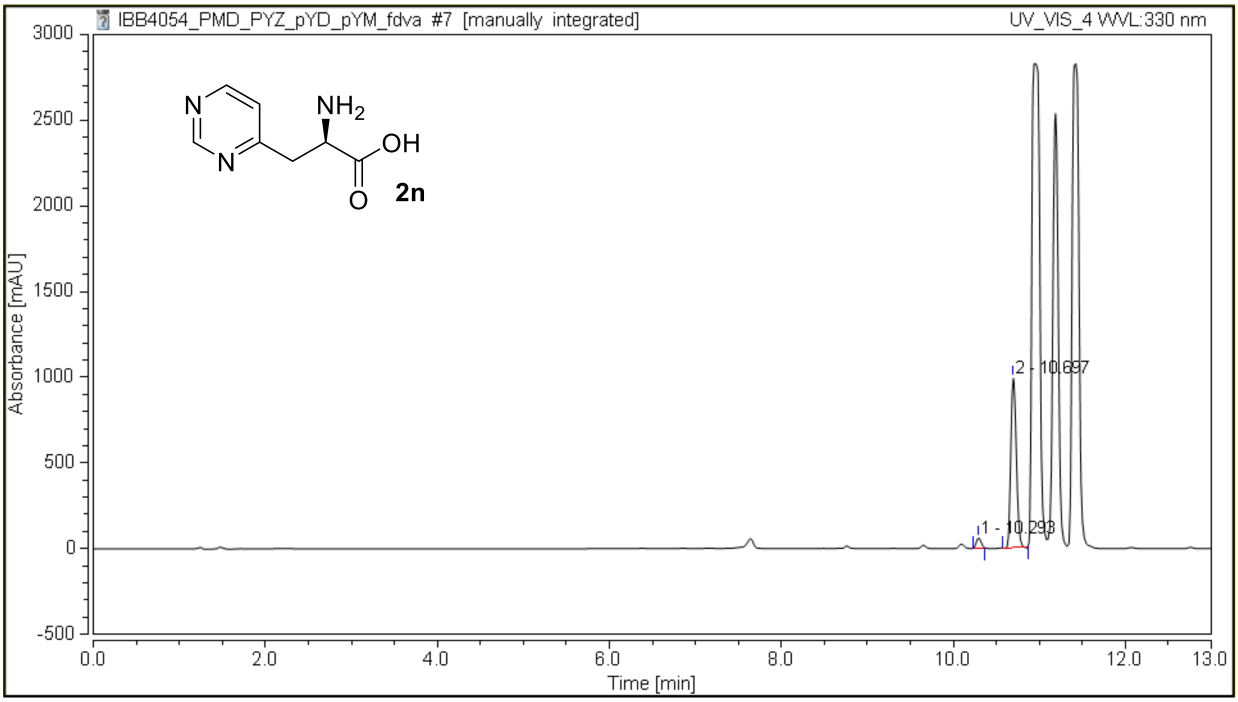

Integration Results							
No.	Peak Name	Retention Time min	Area mAU*min	Height mAU	Relative Area %	Relative Height %	Amount n.a.
1		10.293	3.853	59.844	4.97	5.71	n.a.
2		10.697	73.701	988.581	95.03	94.29	n.a.
**Total:**			**77.554**	**1048.425**	**100.00**	**100.00**	

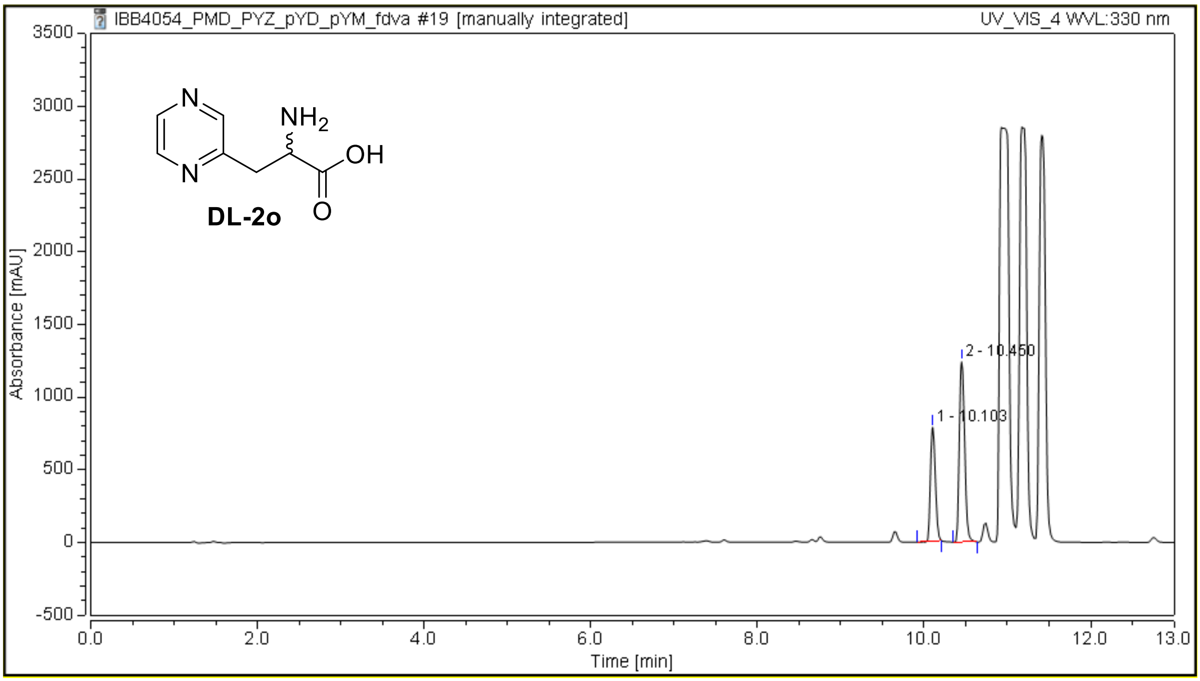

Integration Results							
No.	Peak Name	Retention Time min	Area mAU*min	Height mAU	Relative Area %	Relative Height %	Amount n.a.
1		10.103	53.170	783.131	36.55	38.71	n.a.
2		10.450	92.298	1239.797	63.45	61.29	n.a.
**Total:**			**145.468**	**2022.928**	**100.00**	**100.00**	

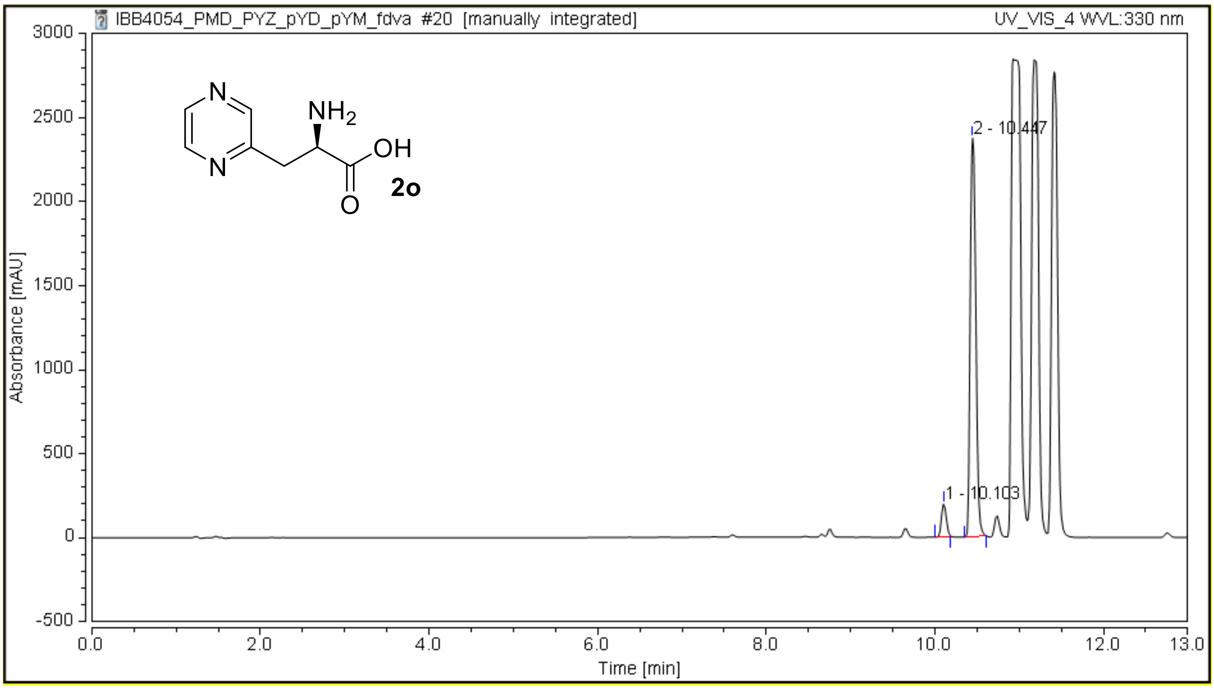

Integration Results							
No.	Peak Name	Retention Time min	Area mAU*min	Height mAU	Relative Area %	Relative Height %	Amount n.a.
1		10.103	13.066	195.993	6.68	7.63	n.a.
2		10.447	182.565	2374.060	93.32	92.37	n.a.
**Total:**			**195.631**	**2570.053**	**100.00**	**100.00**	

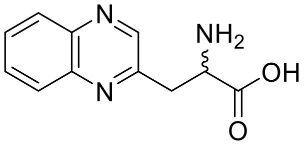

D/L-**2p**

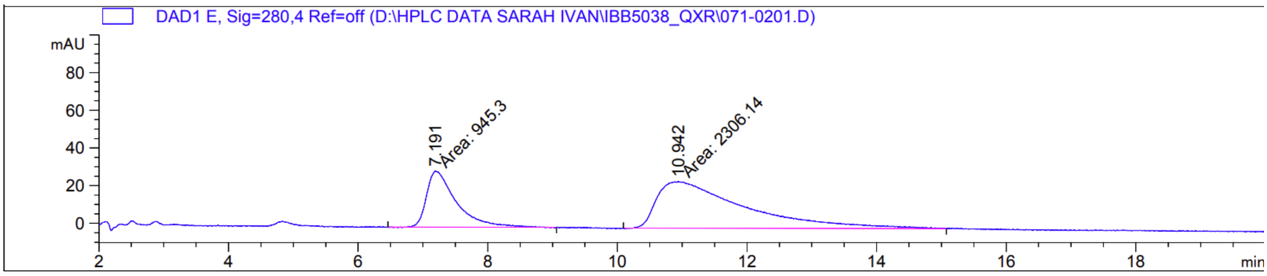

Signal 5: DAD1 E, Sig=280,4 Ref=off

Peak #	RetTime [min]	Type	Width [min]	Area [mAU*s]	Height [mAU]	Area %
1	7.191	MM	0.5297	945.29999	29.74097	29.0733
2	10.942	MM	1.5494	2306.13696	24.80697	70.9267

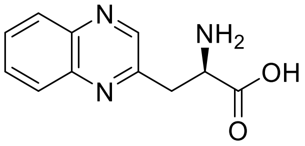

2p

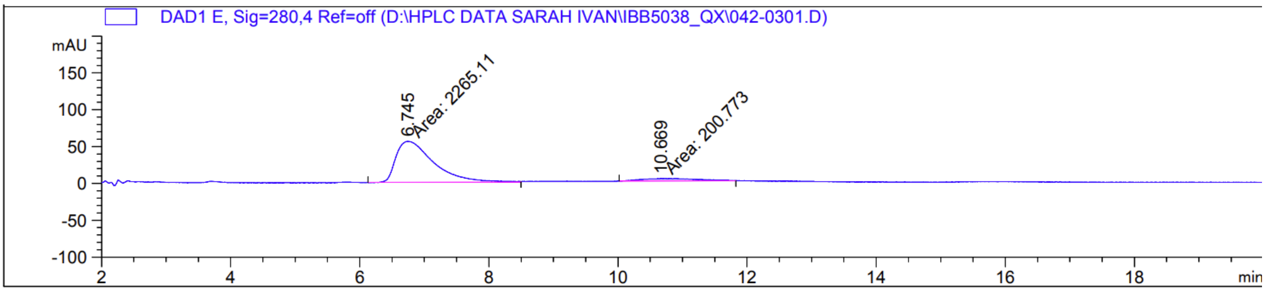

Signal 5: DAD1 E, Sig=280,4 Ref=off

Peak #	RetTime [min]	Type	Width [min]	Area [mAU*s]	Height [mAU]	Area %
1	6.745	MM	0.6783	2265.11401	55.65590	91.8580
2	10.669	MM	1.0024	200.77316	3.33817	8.1420

13. ^{1}H, ^{13}C and ^{19}F NMR spectra of compounds 2a-2p

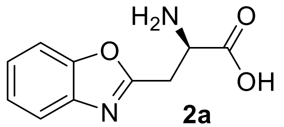

2a

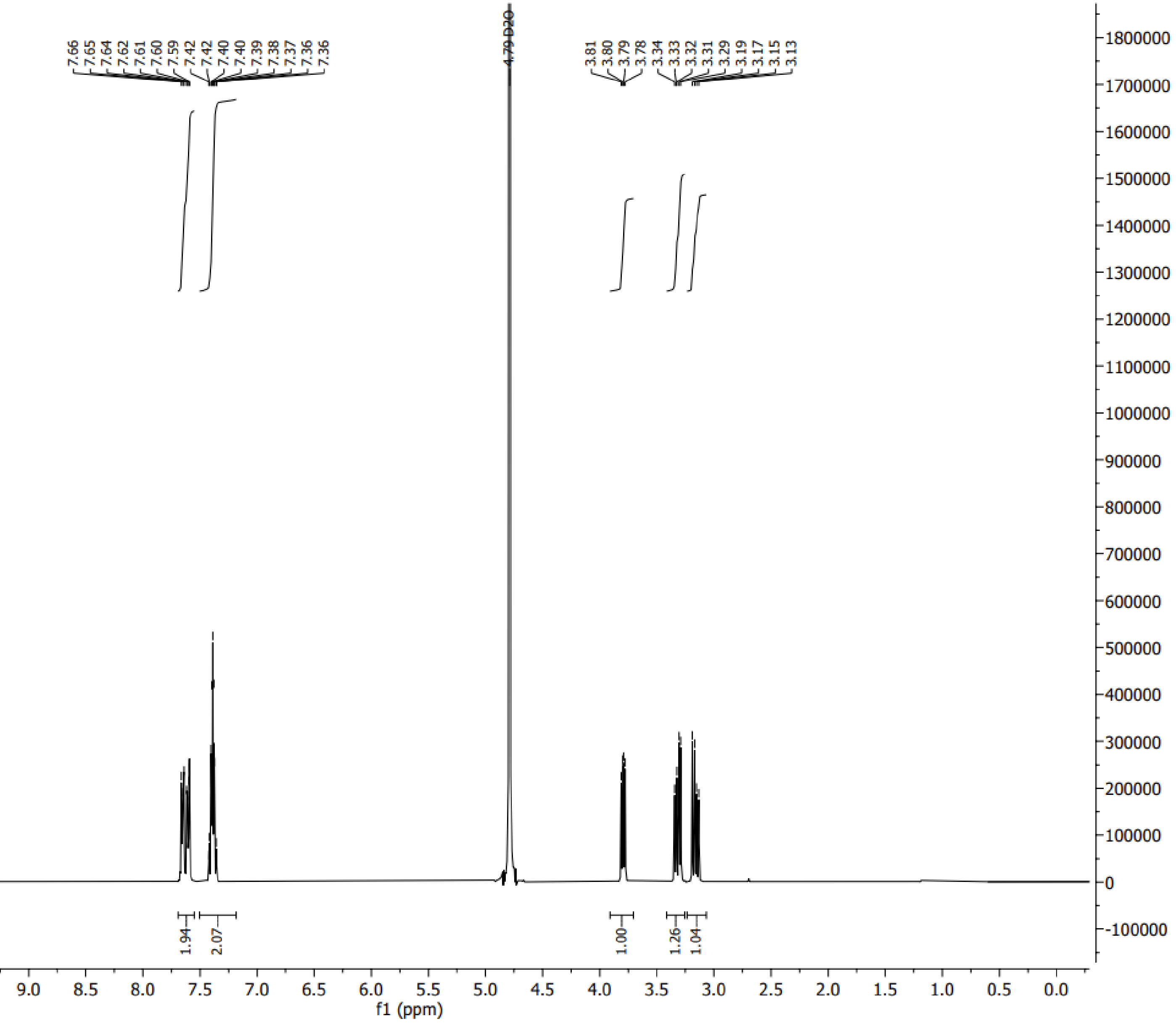

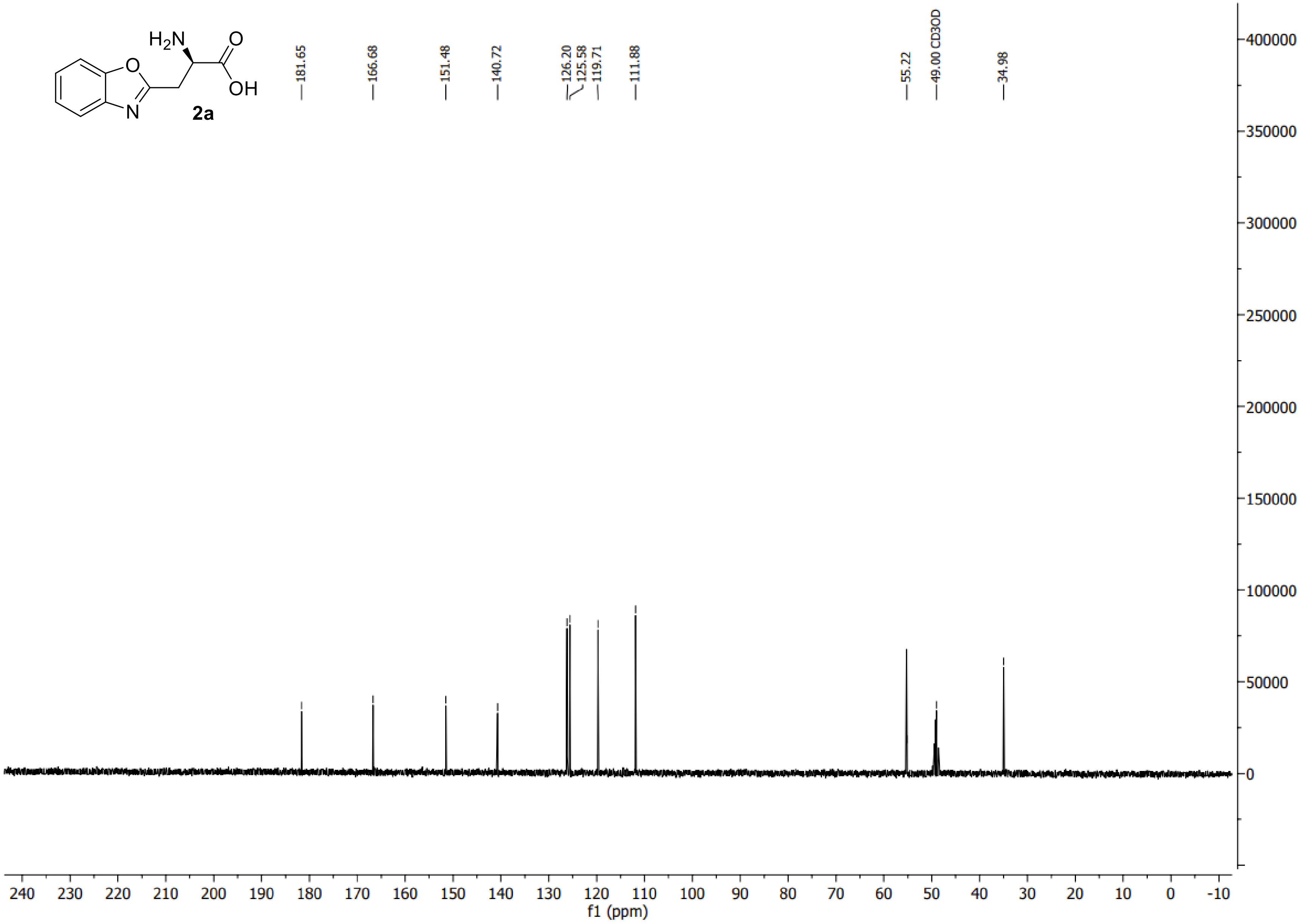

H2N
O
OH
O
N
2a
181.65
166.68
151.48
140.72
126.20
125.58
119.71
111.88
55.22
49.00 CD3OD
34.98
400000
350000
300000
250000
200000
150000
100000
50000
0
240 230 220 210 200 190 180 170 160 150 140 130 120 110 100 90 80 70 60 50 40 30 20 10 0 -10
f1 (ppm)

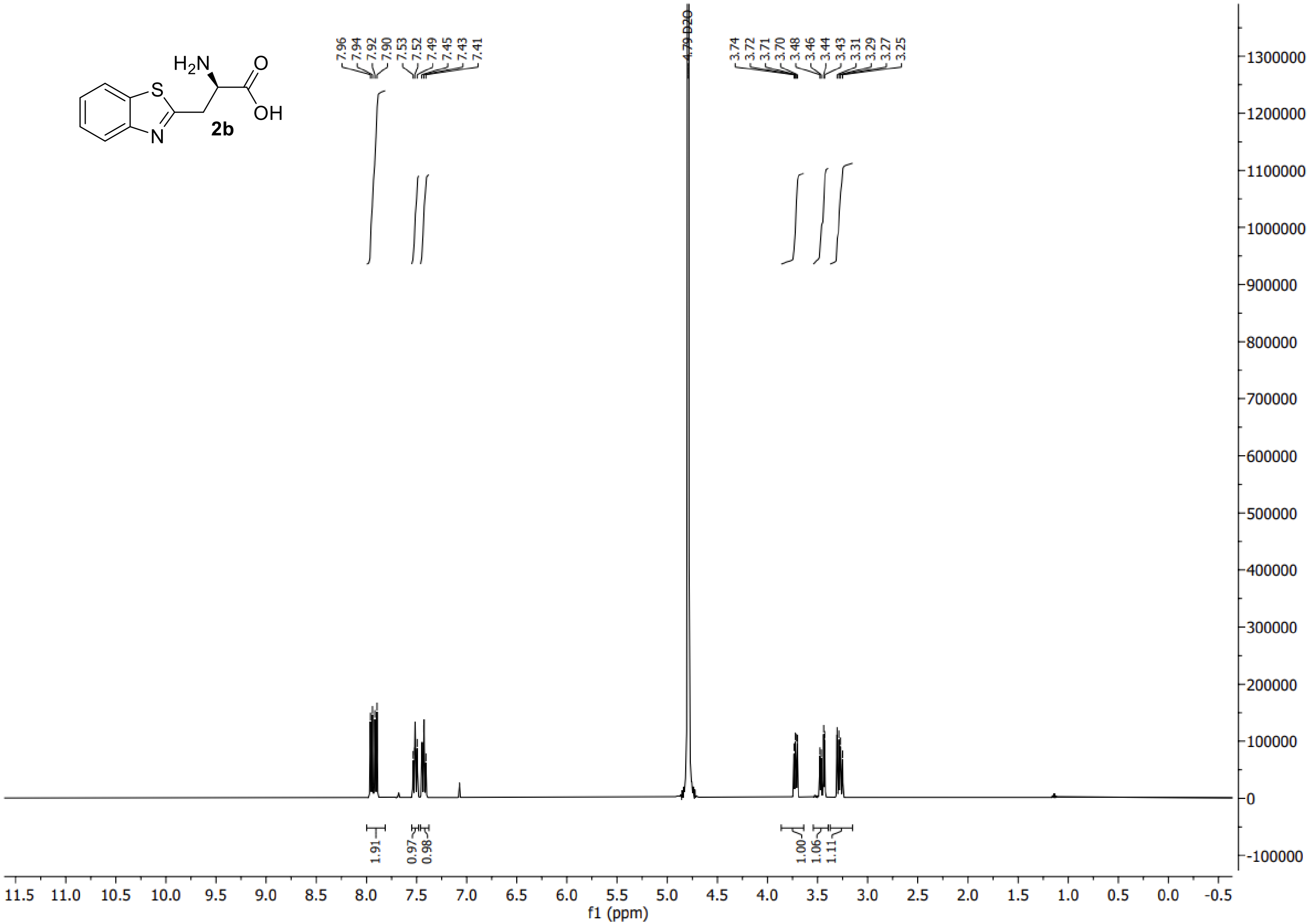
H2N
O
OH
S
N
2b
7.96
7.94
7.92
7.90
7.53
7.52
7.49
7.45
7.43
7.41
4.79 D2O
3.74
3.72
3.71
3.70
3.48
3.46
3.44
3.43
3.31
3.29
3.27
3.25
1.91
0.97
0.98
1.00
1.06
1.11
f1 (ppm)

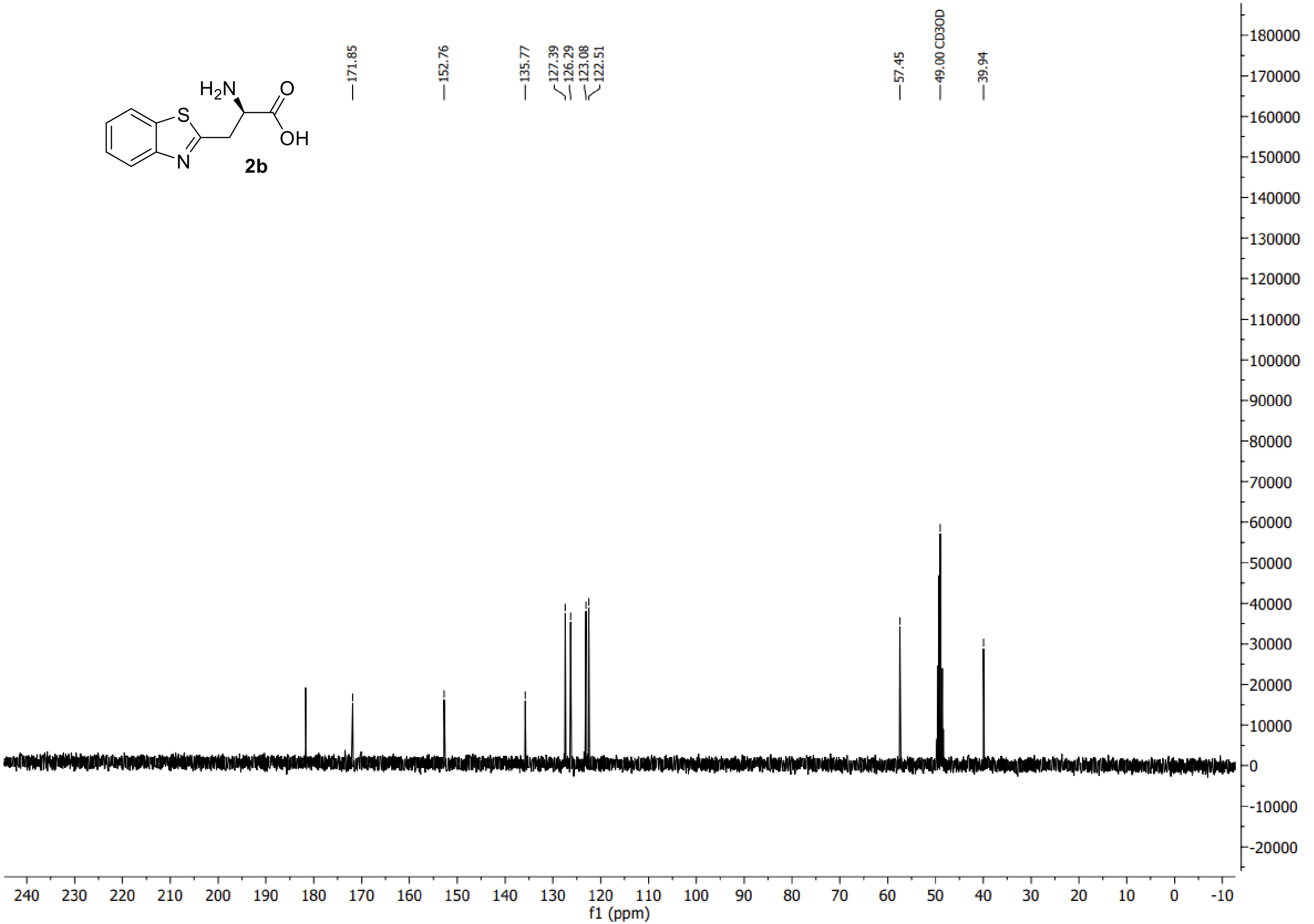
H2N
O
OH
S
N
2b
171.85
152.76
135.77
127.39
126.29
123.08
122.51
57.45
49.00 CD3OD
39.94
f1 (ppm)

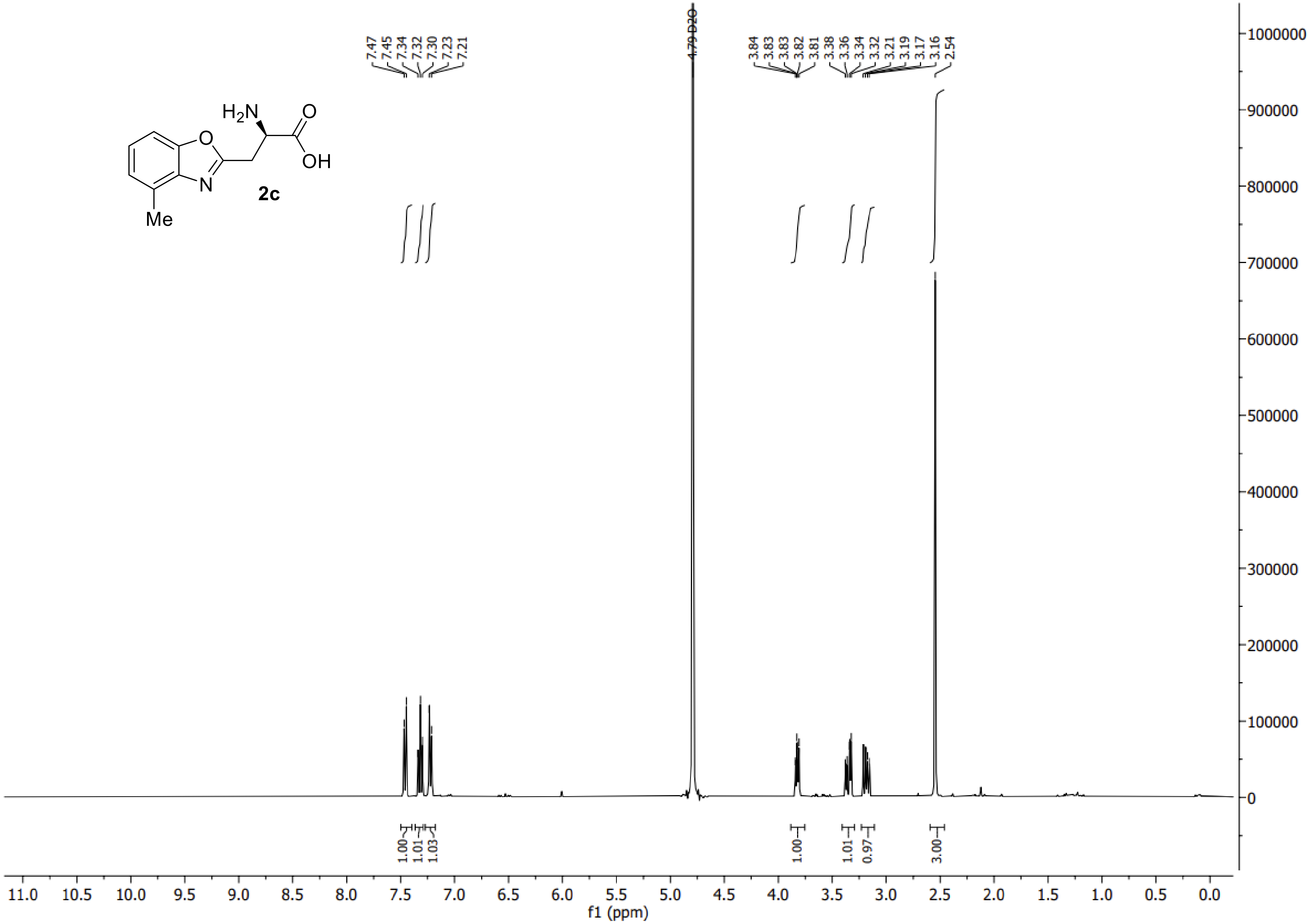
7.47
7.45
7.34
7.32
7.30
7.23
7.21
4.79 D2O
3.84
3.83
3.83
3.82
3.81
3.38
3.36
3.34
3.32
3.21
3.19
3.17
3.16
2.54
H2N
O
OH
O
N
Me
2c
1.00
1.01
1.03
1.00
1.01
0.97
3.00
f1 (ppm)

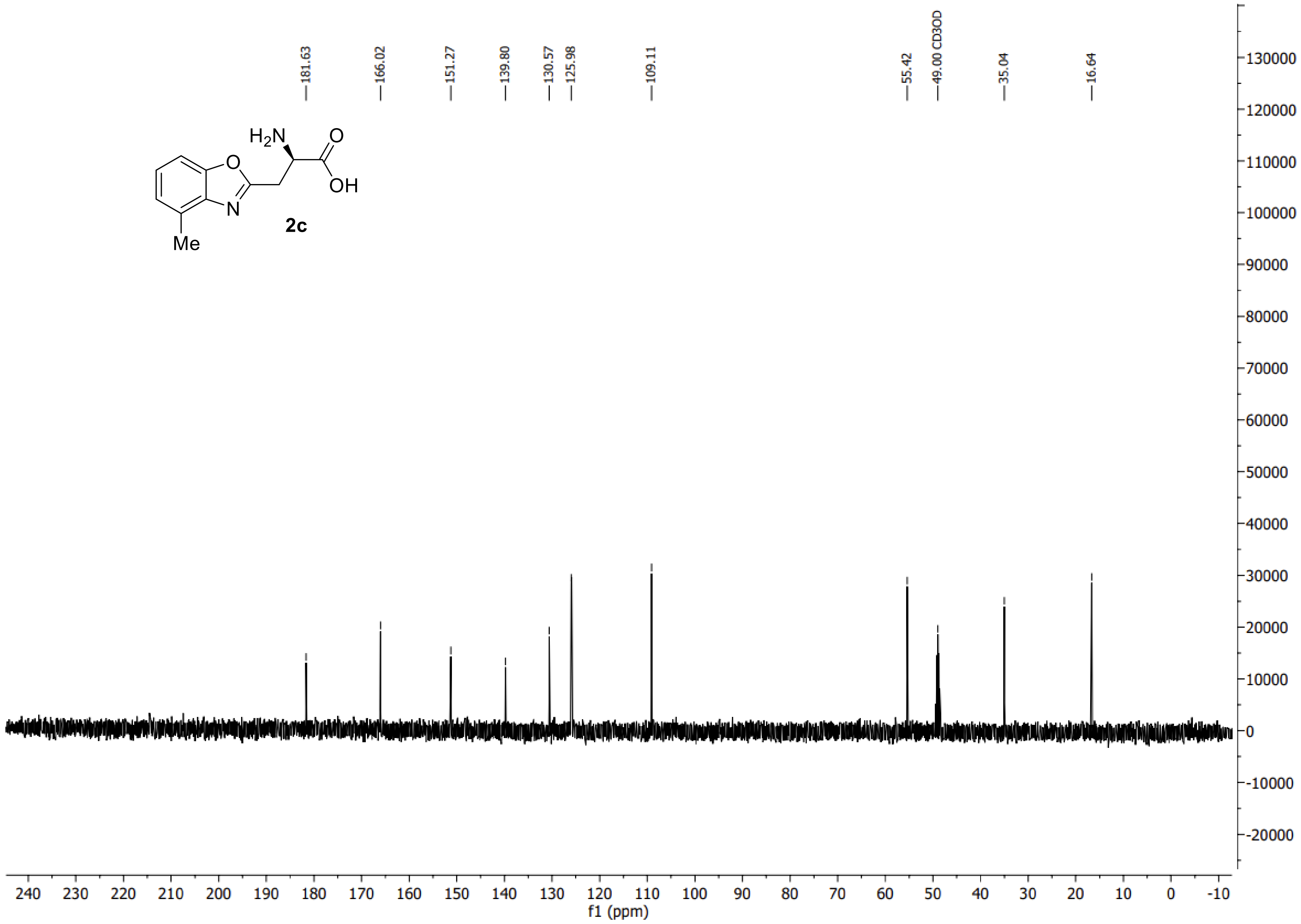
181.63
166.02
151.27
139.80
130.57
125.98
109.11
55.42
49.00 CD3OD
35.04
16.64
H2N
O
OH
O
N
Me
2c
f1 (ppm)

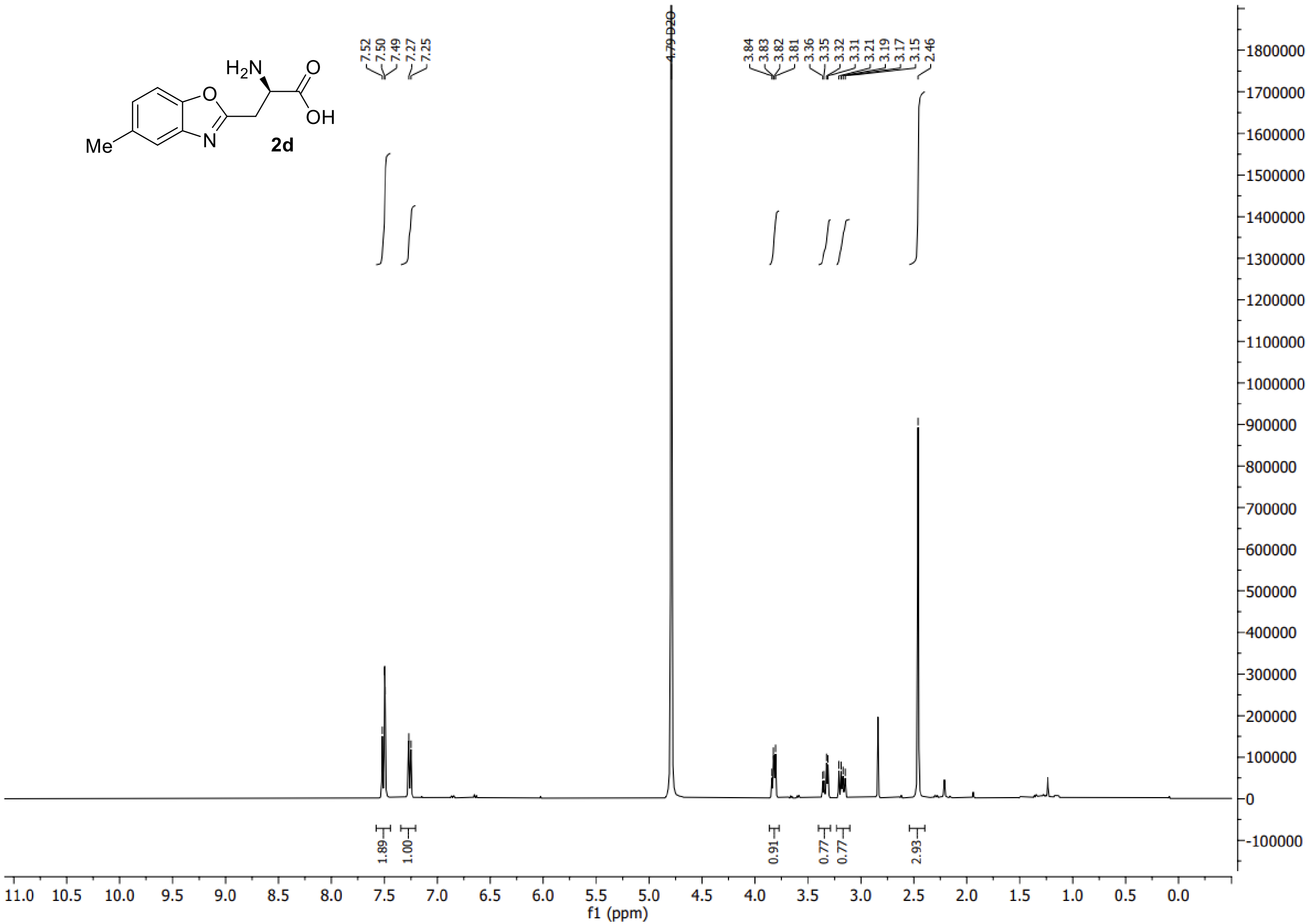
H2N
O
OH
Me
N
O
2d
7.52
7.50
7.49
7.27
7.25
4.79 D2O
3.84
3.83
3.82
3.81
3.36
3.35
3.32
3.31
3.21
3.19
3.17
3.15
2.46
1.89
1.00
0.91
0.77
0.77
2.93
f1 (ppm)

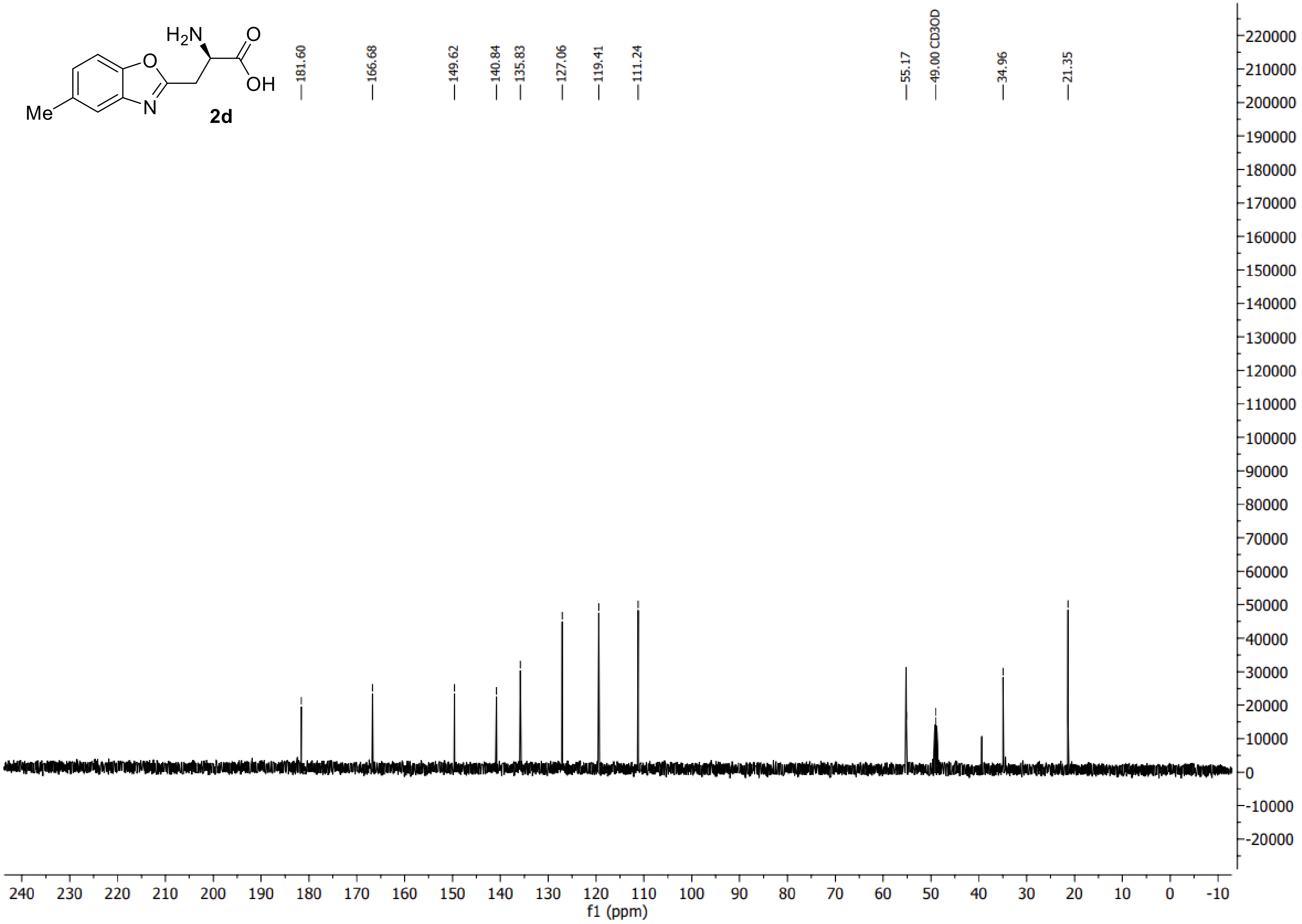
H2N
O
OH
O
N
Me
2d
181.60
166.68
149.62
140.84
135.83
127.06
119.41
111.24
55.17
49.00 CD3OD
34.96
21.35
f1 (ppm)

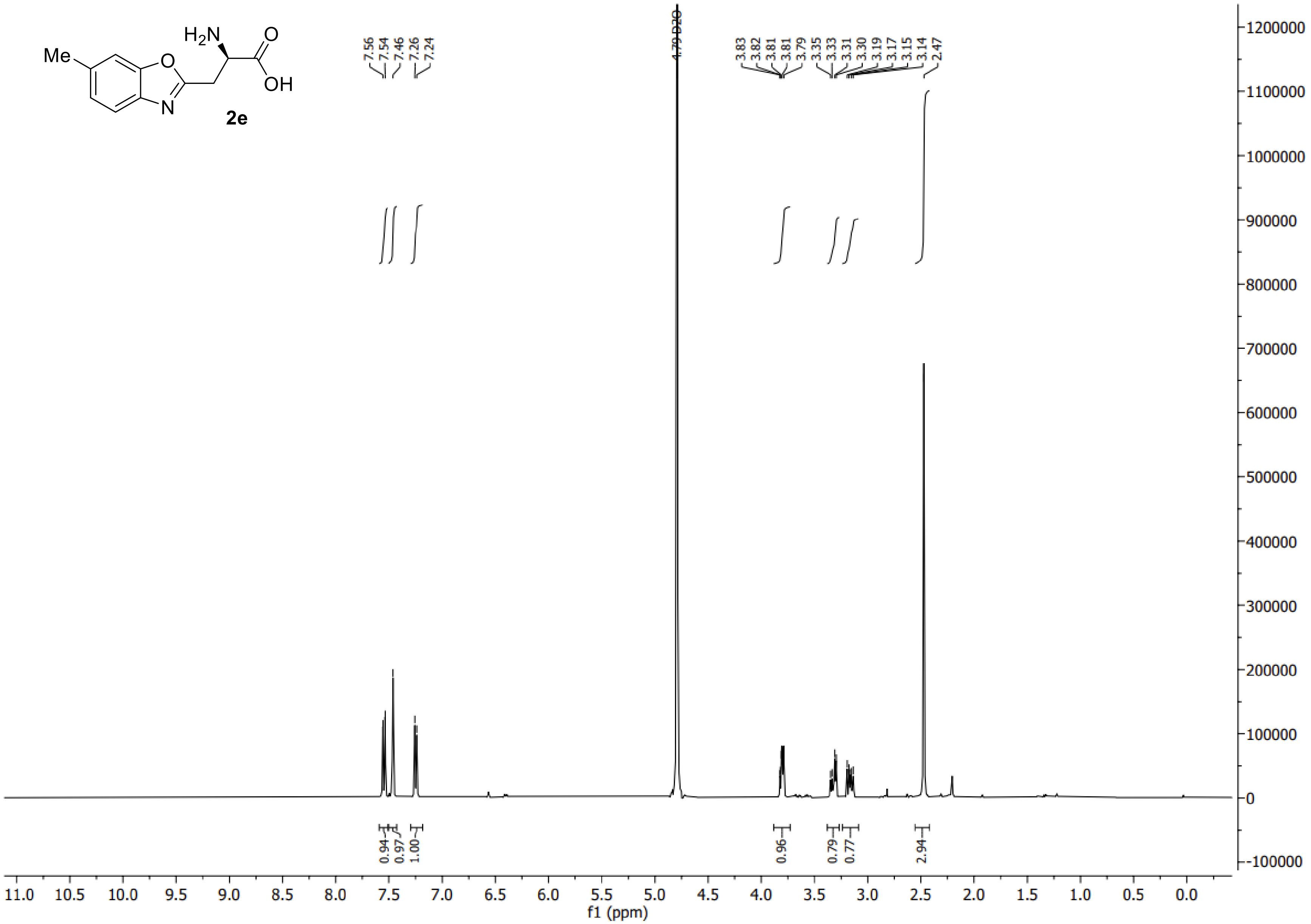
Me
O
H2N
O
OH
N
7.56
7.54
7.46
7.26
7.24
4.79 D2O
3.83
3.82
3.81
3.81
3.79
3.35
3.33
3.31
3.30
3.19
3.17
3.15
3.14
2.47
0.94
0.97
1.00
0.96
0.79
0.77
2.94
f1 (ppm)

2e

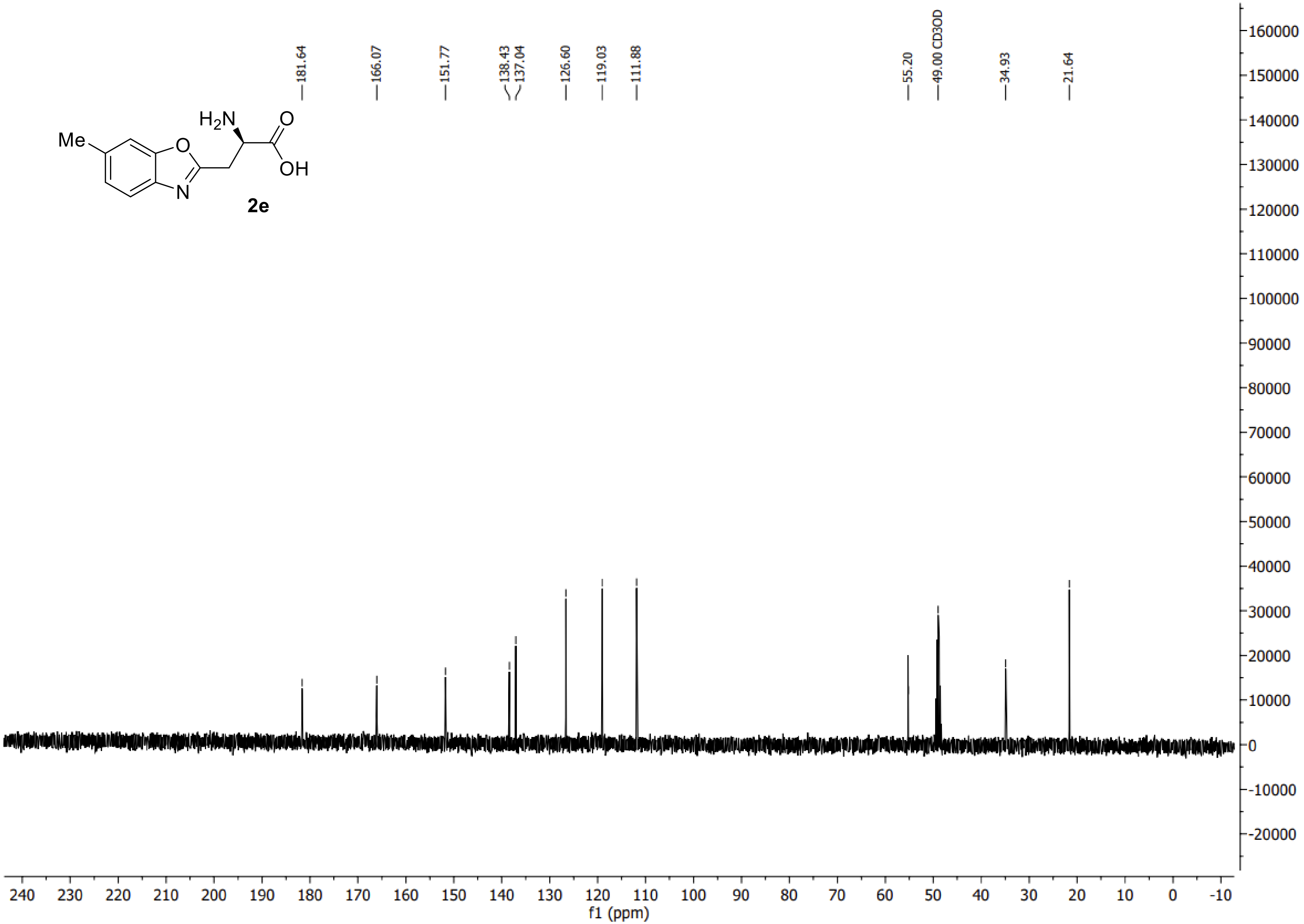

181.64
166.07
151.77
138.43
137.04
126.60
119.03
111.88
55.20
49.00 CD3OD
34.93
21.64
Me
H_2N
O
OH
N
f1 (ppm)

2e

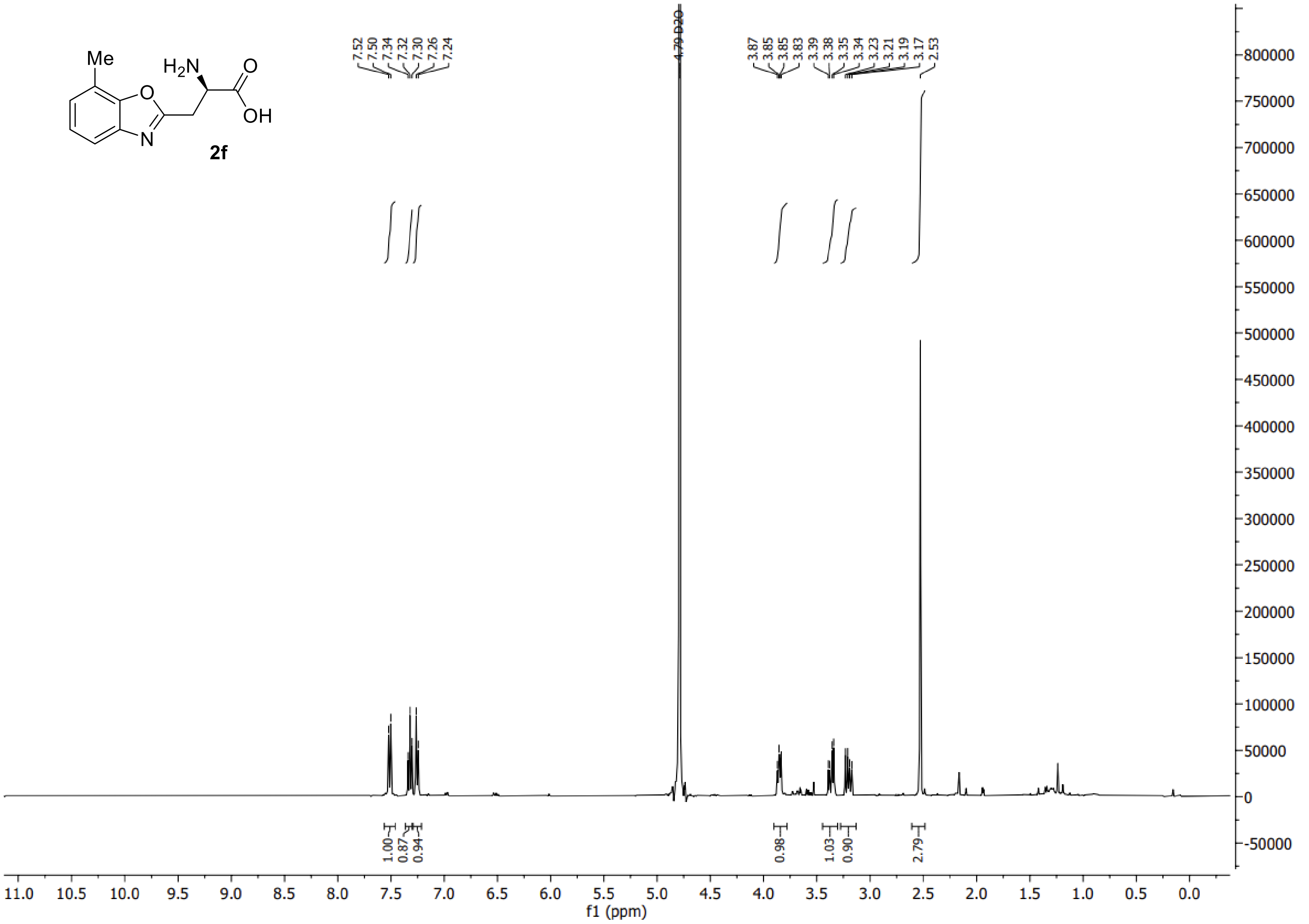
Me
H2N
O
OH
N
2f
7.52
7.50
7.34
7.32
7.30
7.26
7.24
4.79 D2O
3.87
3.85
3.85
3.83
3.39
3.38
3.35
3.34
3.23
3.21
3.19
3.17
2.53
1.00
0.87
0.94
0.98
1.03
0.90
2.79
f1 (ppm)

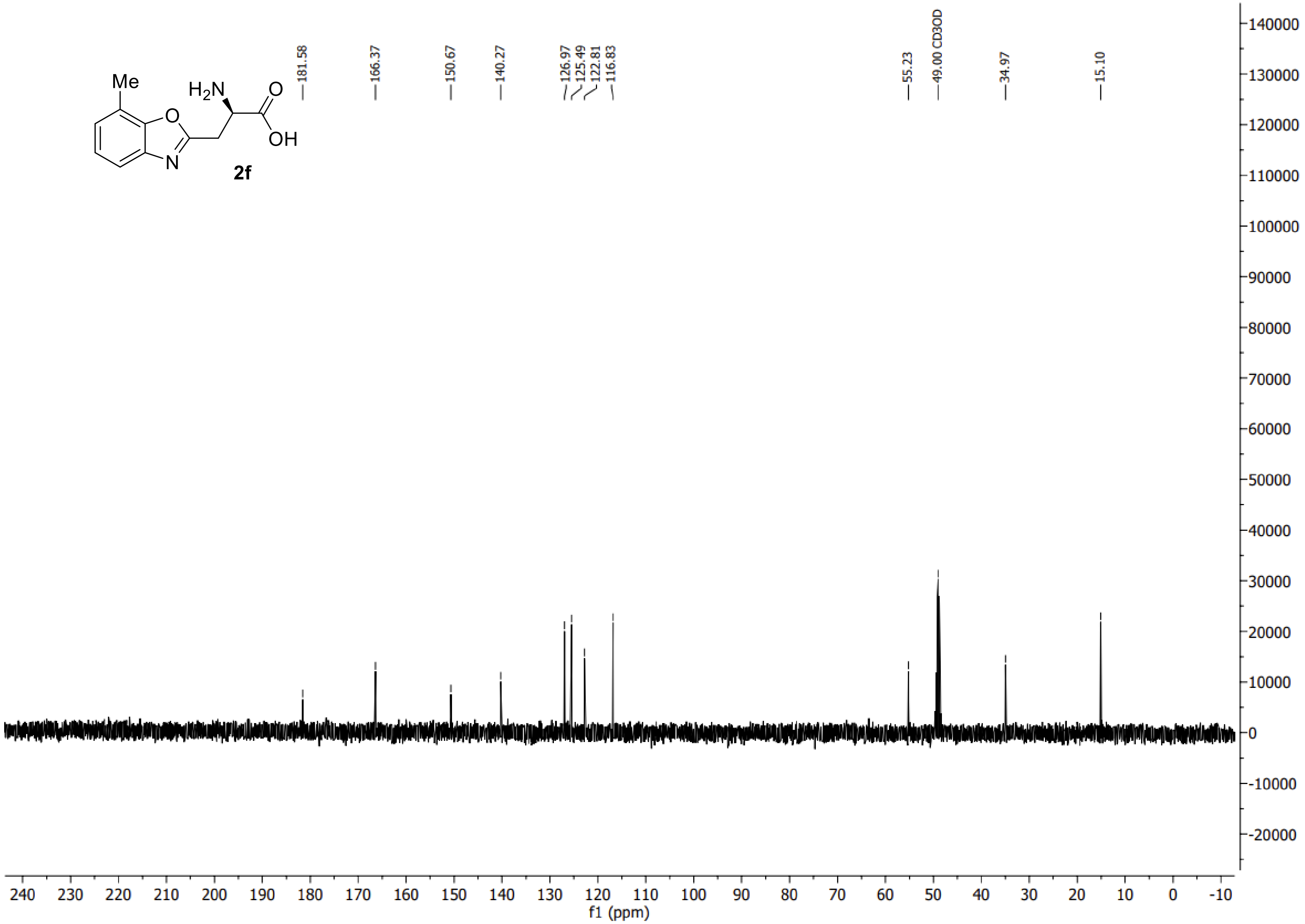

Me
H_2N
O
OH
O
N
2f
181.58
166.37
150.67
140.27
126.97
125.49
122.81
116.83
55.23
49.00 CD3OD
34.97
15.10
f1 (ppm)

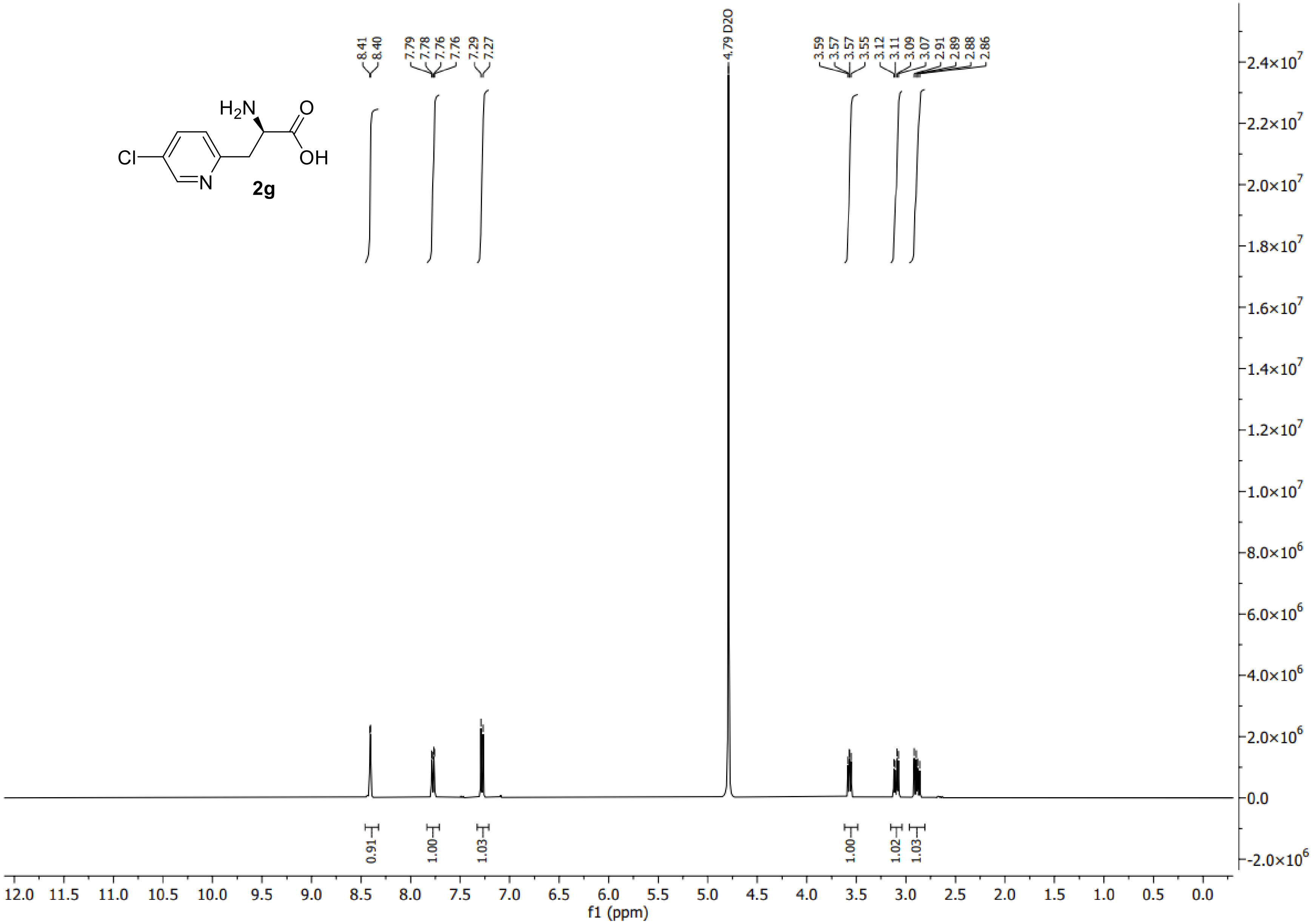

H2N
O
OH
Cl
N
2g
8.41
8.40
7.79
7.78
7.76
7.76
7.29
7.27
4.79 D2O
3.59
3.57
3.57
3.55
3.12
3.11
3.09
3.07
2.91
2.89
2.88
2.86
0.91
1.00
1.03
1.00
1.02
1.03
f1 (ppm)

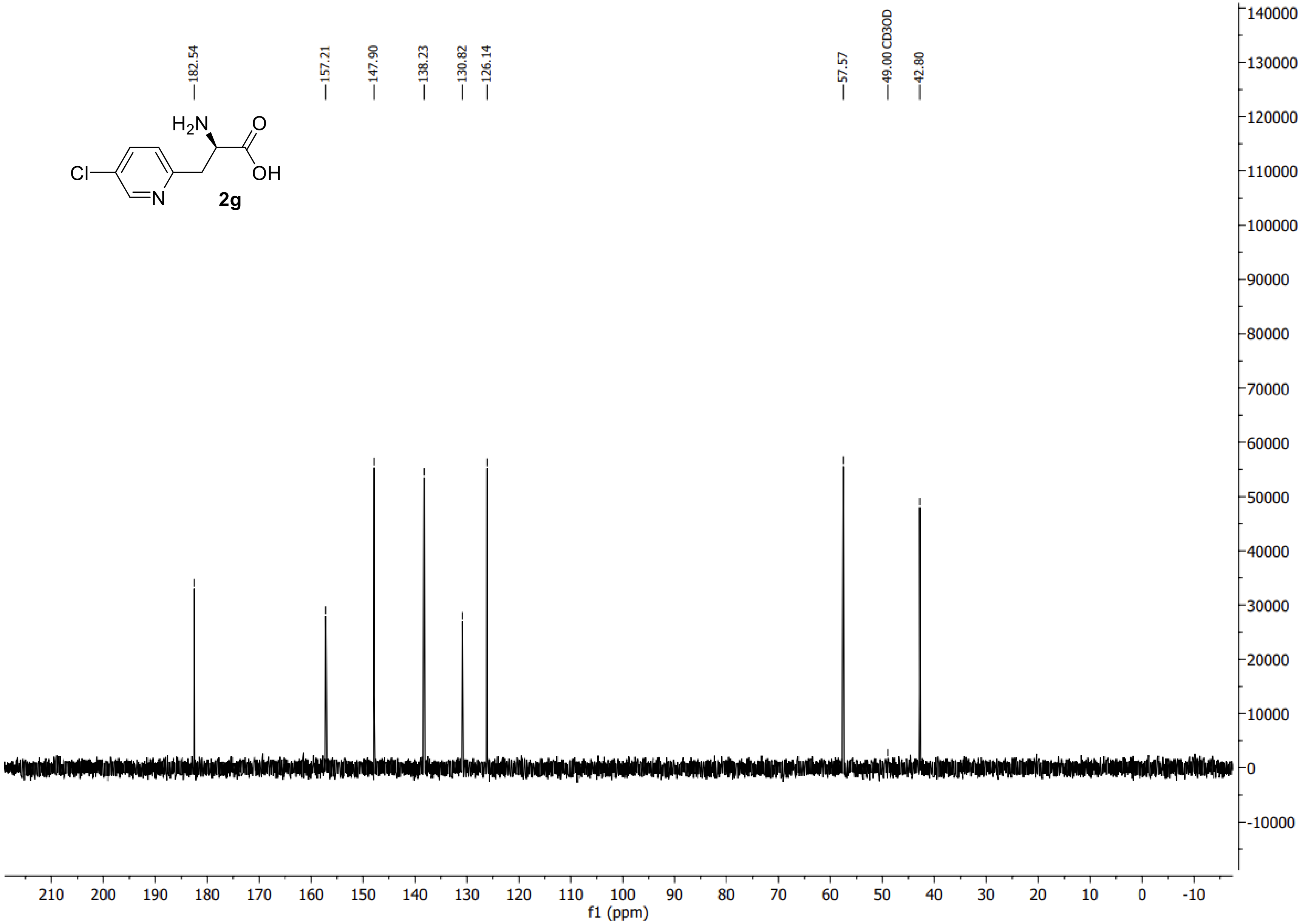
182.54
157.21
147.90
138.23
130.82
126.14
57.57
49.00 CD3OD
42.80
H2N
O
OH
Cl
N
2g
140000
130000
120000
110000
100000
90000
80000
70000
60000
50000
40000
30000
20000
10000
0
-10000
210 200 190 180 170 160 150 140 130 120 110 100 90 80 70 60 50 40 30 20 10 0 -10
f1 (ppm)

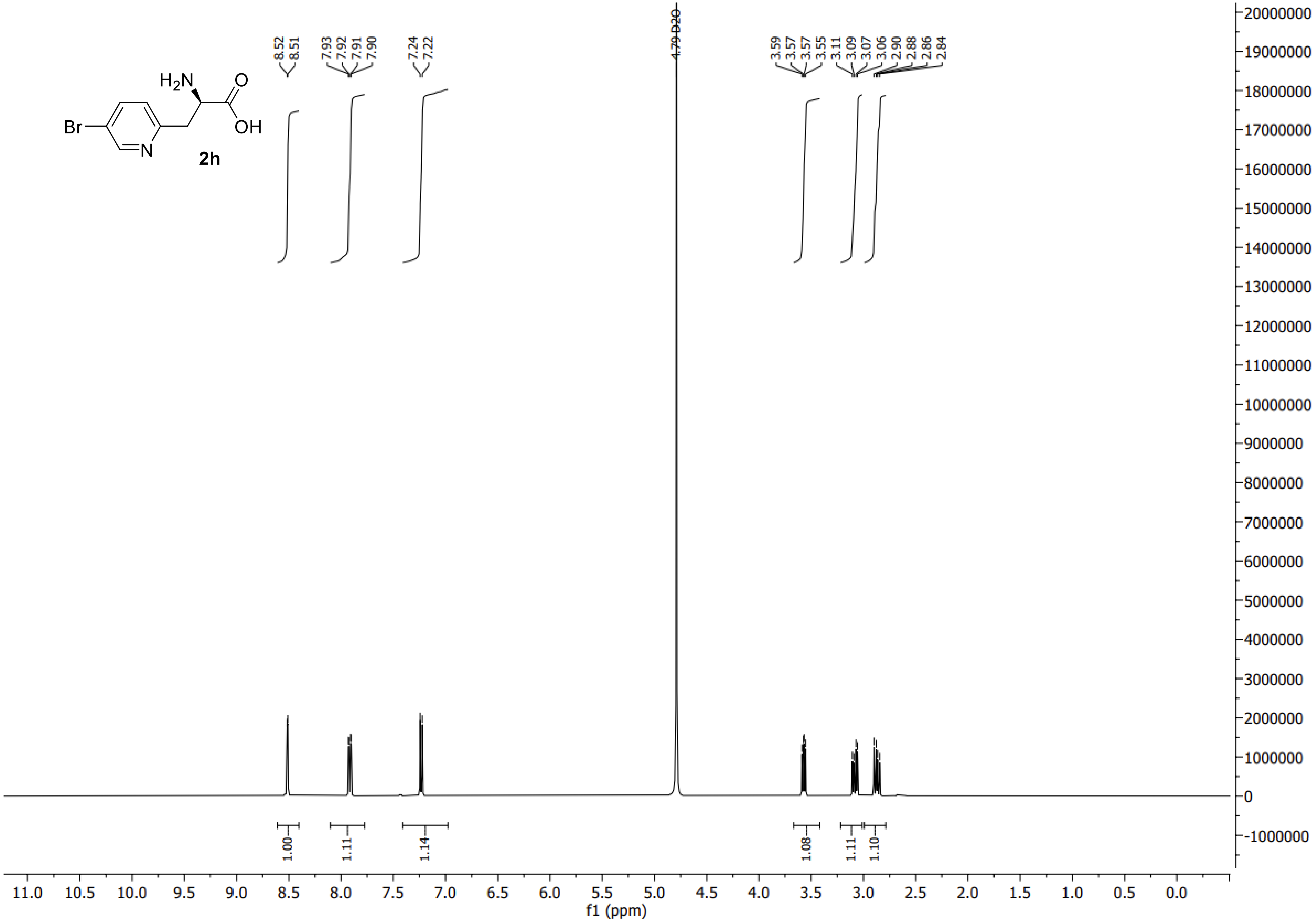

2h
8.52
8.51
7.93
7.92
7.91
7.90
7.24
7.22
4.79 D2O
3.59
3.57
3.57
3.55
3.11
3.09
3.07
3.06
2.90
2.88
2.86
2.84
1.00
1.11
1.14
1.08
1.11
1.10
f1 (ppm)

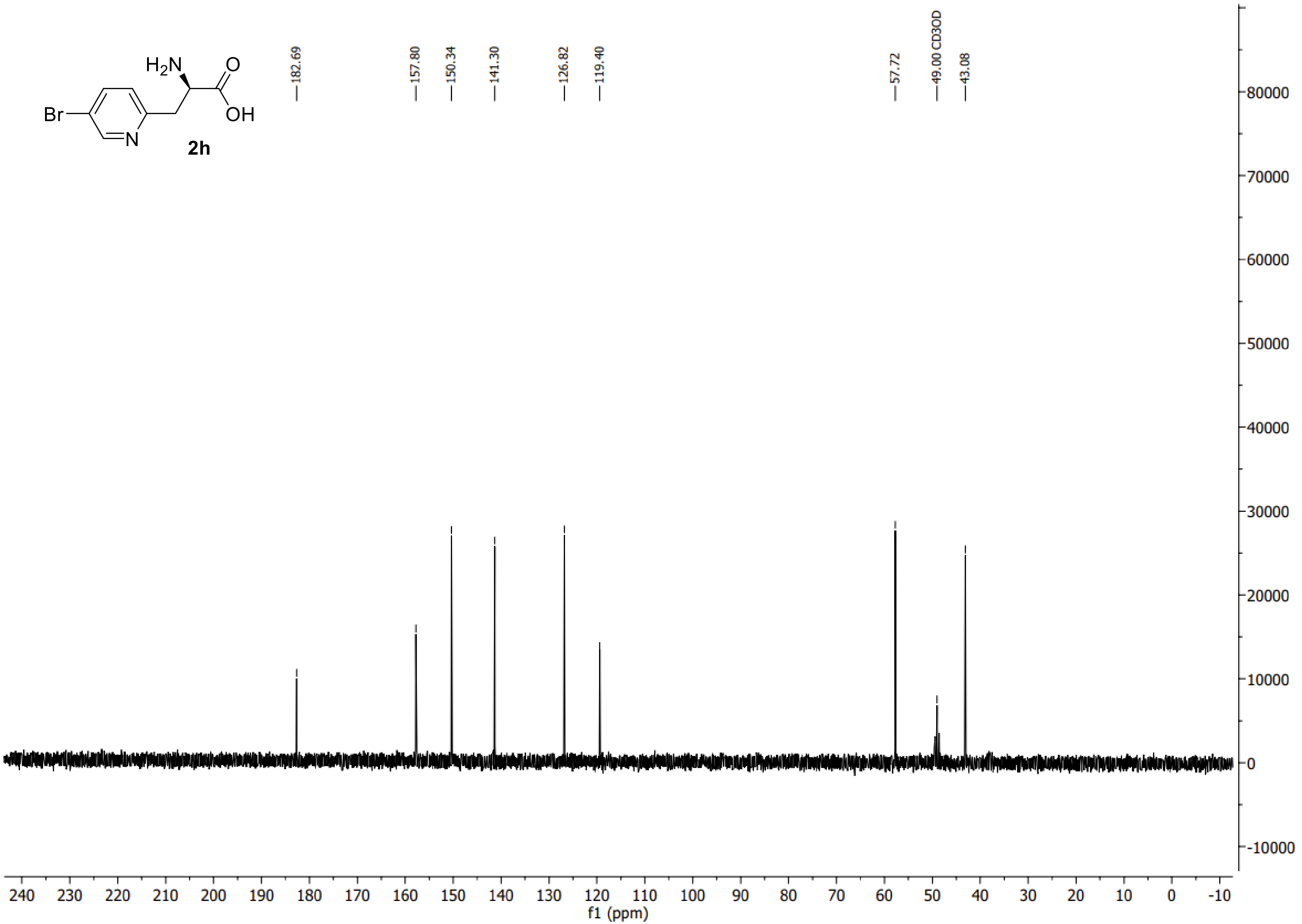

H2N
O
OH
Br
N
2h
182.69
157.80
150.34
141.30
126.82
119.40
57.72
49.00 CD3OD
43.08
80000
70000
60000
50000
40000
30000
20000
10000
0
-10000
240 230 220 210 200 190 180 170 160 150 140 130 120 110 100 90 80 70 60 50 40 30 20 10 0 -10
f1 (ppm)

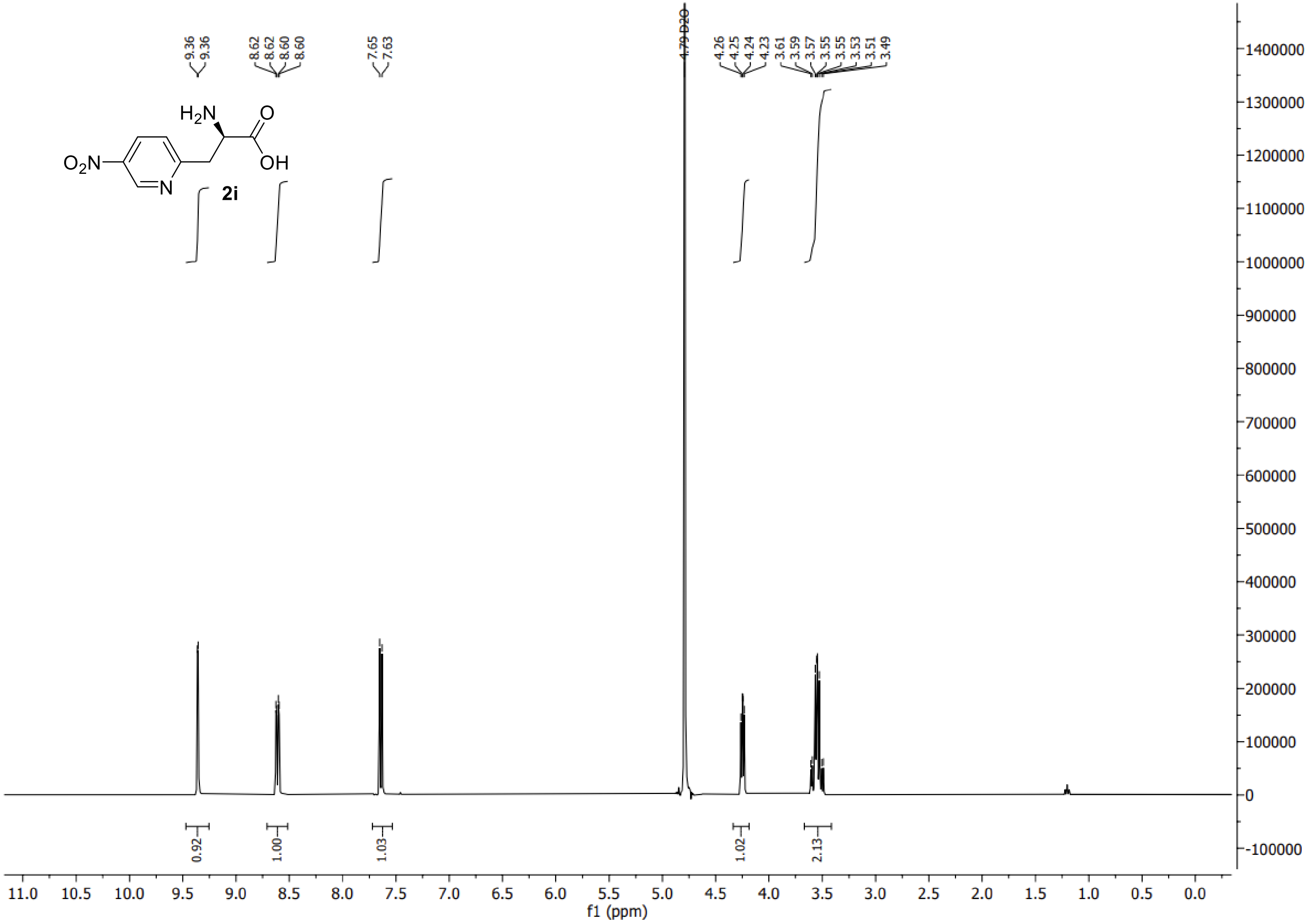

9.36
9.36
8.62
8.62
8.60
8.60
7.65
7.63
4.79 D2O
4.26
4.25
4.24
4.23
3.61
3.59
3.57
3.55
3.55
3.53
3.51
3.49
H2N
O
OH
O2N
N
2i
0.92
1.00
1.03
1.02
2.13
f1 (ppm)

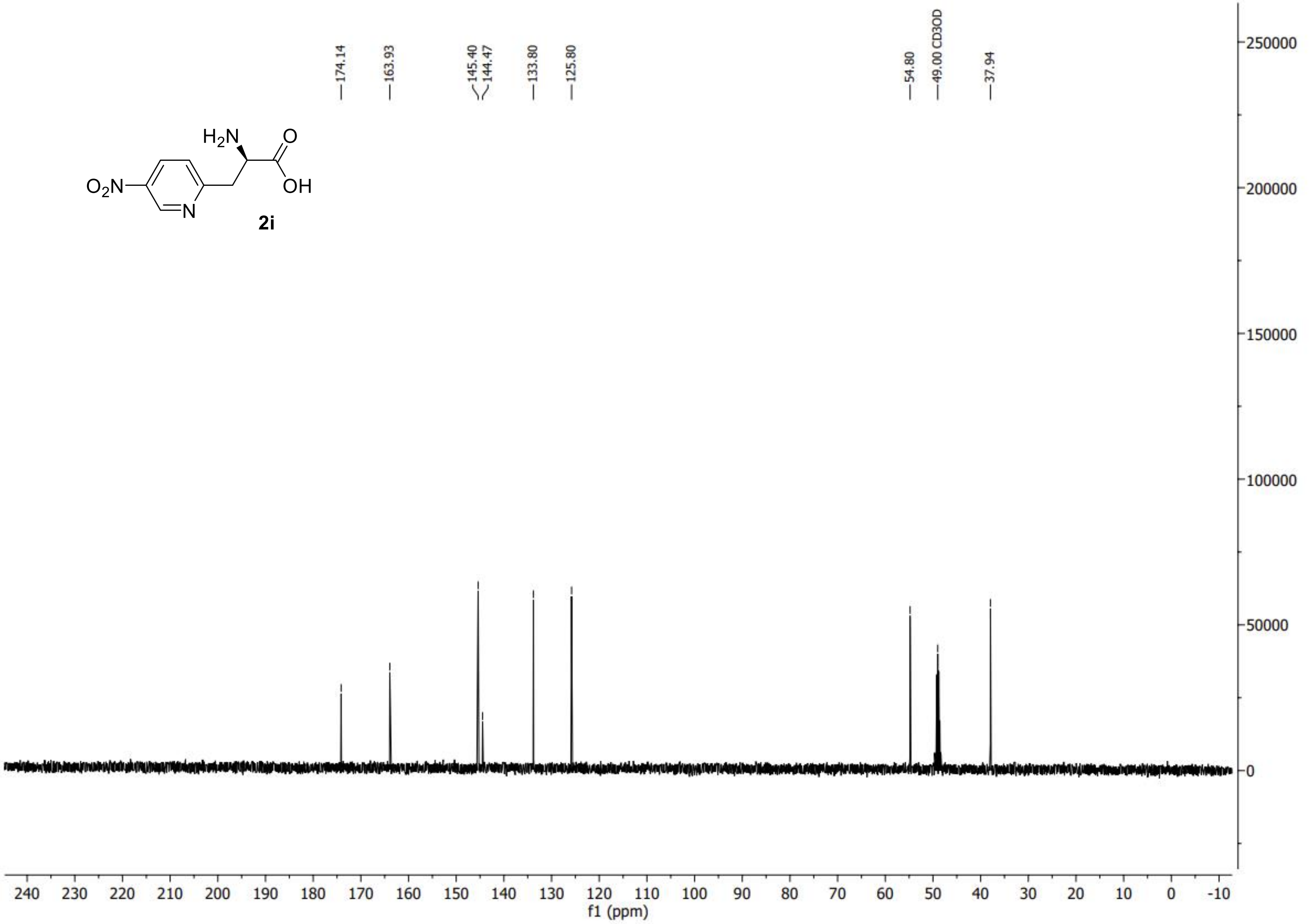
174.14
163.93
145.40
144.47
133.80
125.80
54.80
49.00 CD3OD
37.94
H_2N
O
OH
O_2N
N
2i
250000
200000
150000
100000
50000
0
240 230 220 210 200 190 180 170 160 150 140 130 120 110 100 90 80 70 60 50 40 30 20 10 0 -10
f1 (ppm)

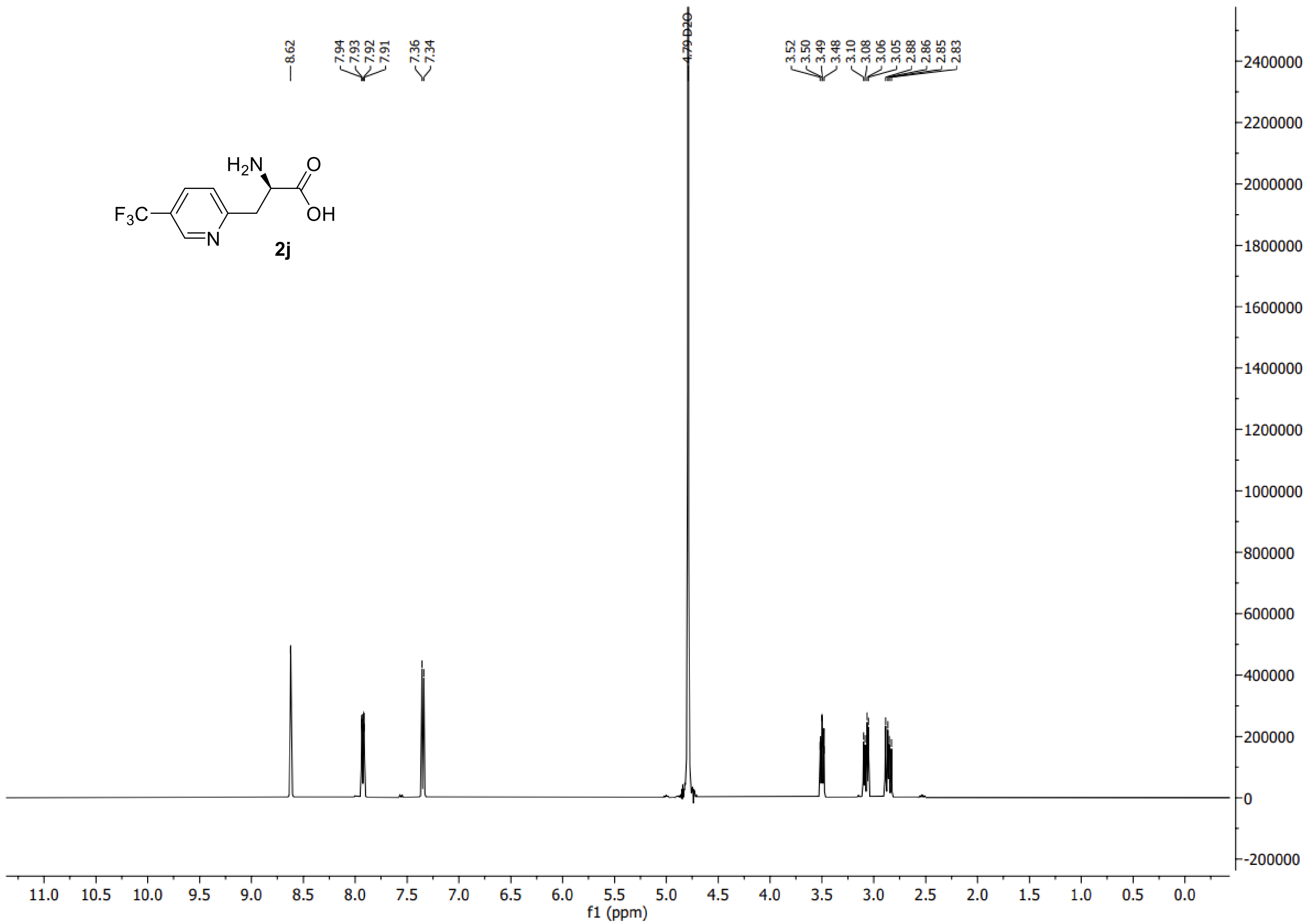

8.62
7.94
7.93
7.92
7.91
7.36
7.34
4.79 D2O
3.52
3.50
3.49
3.48
3.10
3.08
3.06
3.05
2.88
2.86
2.85
2.83
H2N
O
OH
F3C
N
2j
f1 (ppm)

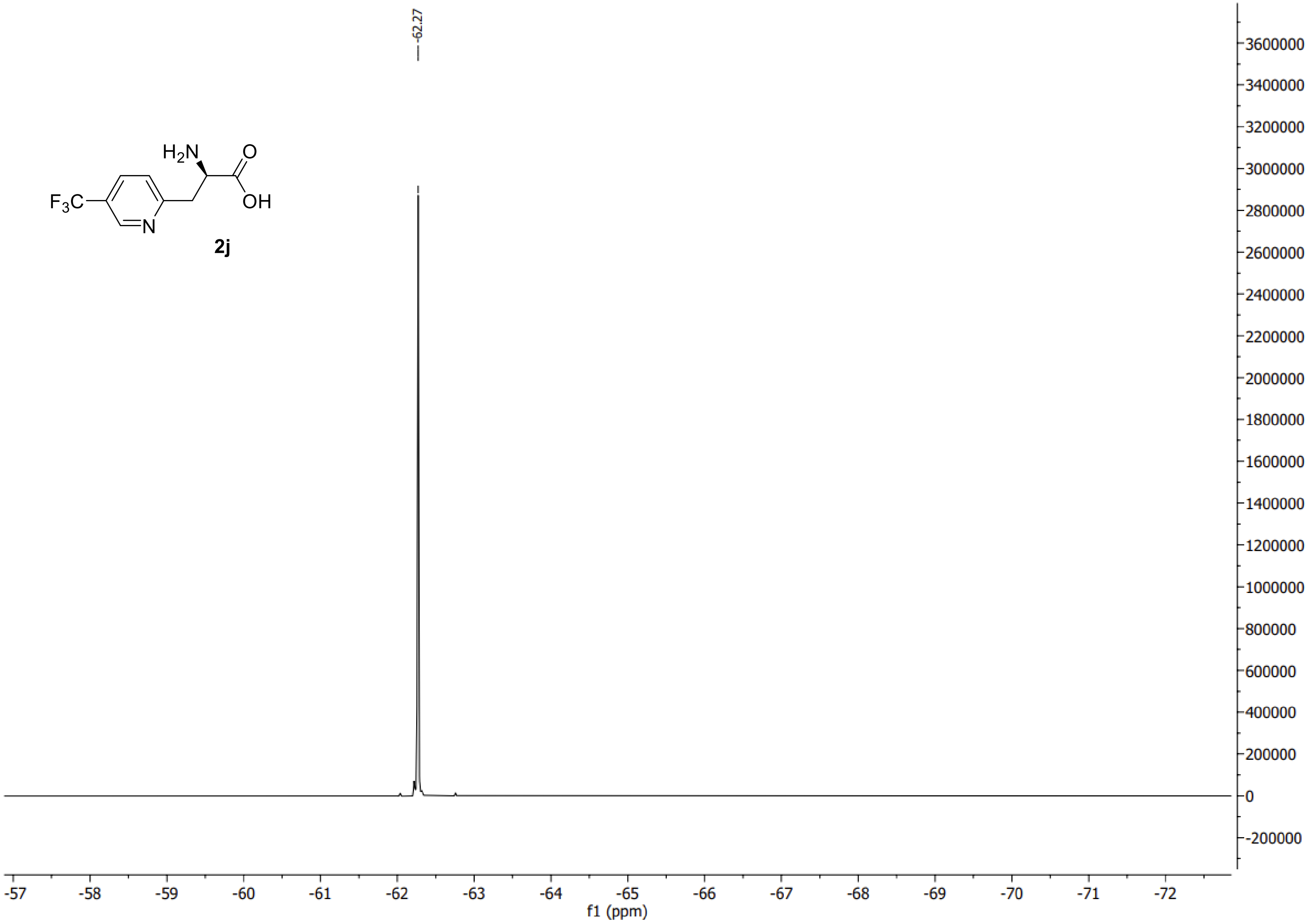

-62.27
H2N
O
OH
F3C
N
2j
3600000
3400000
3200000
3000000
2800000
2600000
2400000
2200000
2000000
1800000
1600000
1400000
1200000
1000000
800000
600000
400000
200000
0
-200000
-57
-58
-59
-60
-61
-62
-63
-64
-65
-66
-67
-68
-69
-70
-71
-72
f1 (ppm)

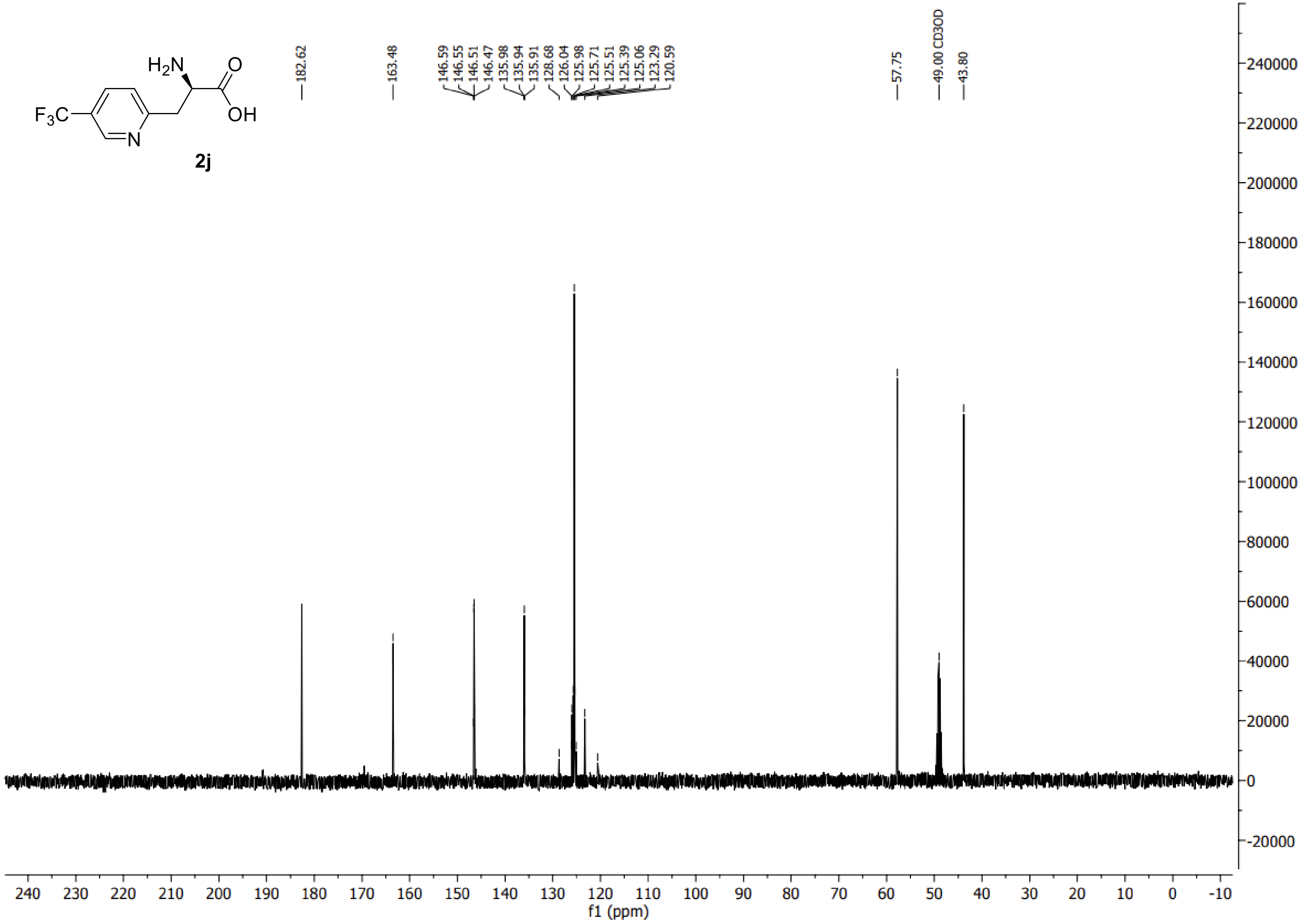

H2N
O
OH
F3C
N
2j
182.62
163.48
146.59
146.55
146.51
146.47
135.98
135.94
135.91
128.68
126.04
125.98
125.71
125.51
125.39
125.06
123.29
120.59
57.75
49.00 CD3OD
43.80
f1 (ppm)

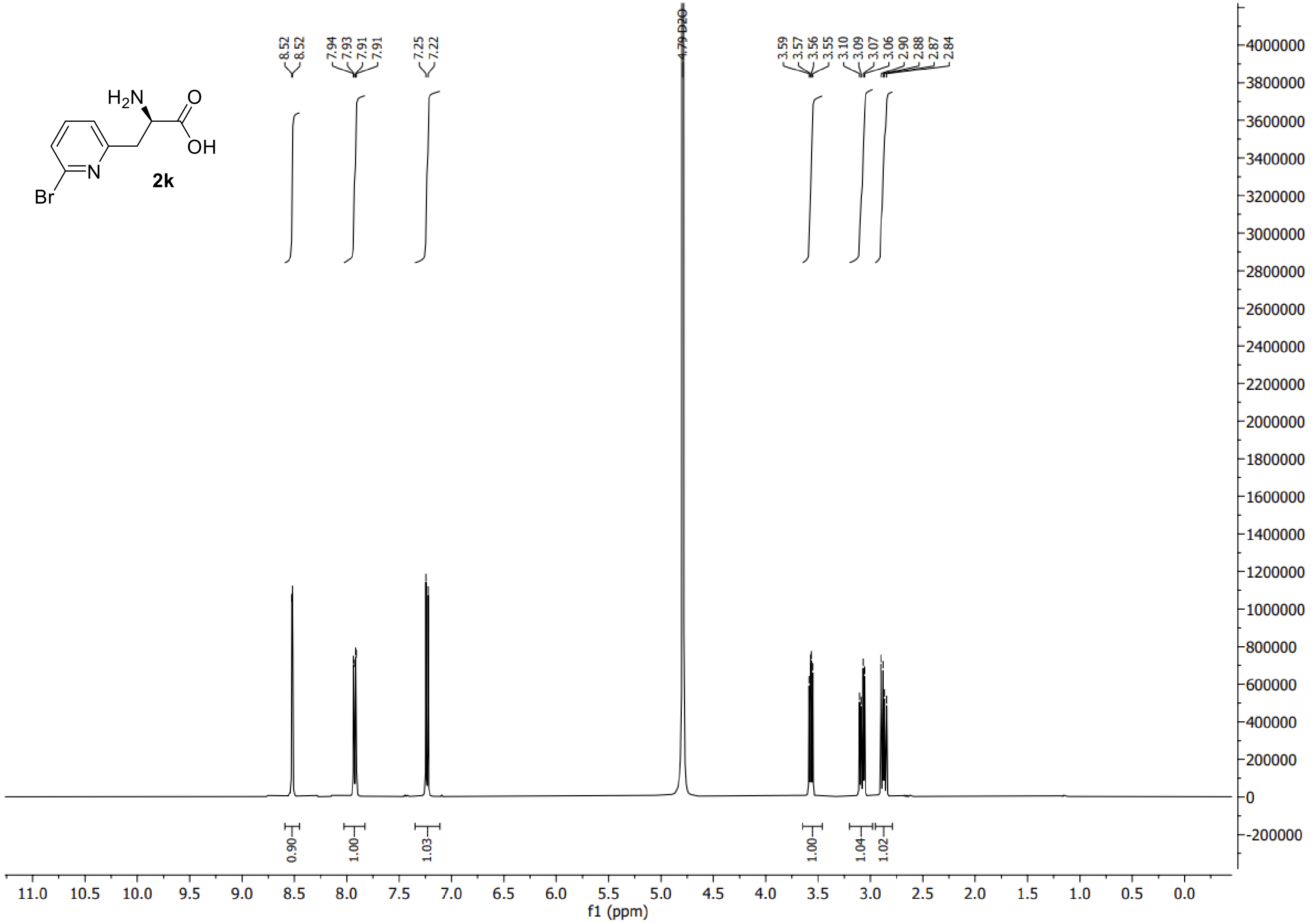

H2N
O
OH
N
Br
2k
8.52
8.52
7.94
7.93
7.91
7.91
7.25
7.22
4.79 D2O
3.59
3.57
3.56
3.55
3.10
3.09
3.07
3.06
2.90
2.88
2.87
2.84
0.90
1.00
1.03
1.00
1.04
1.02
f1 (ppm)

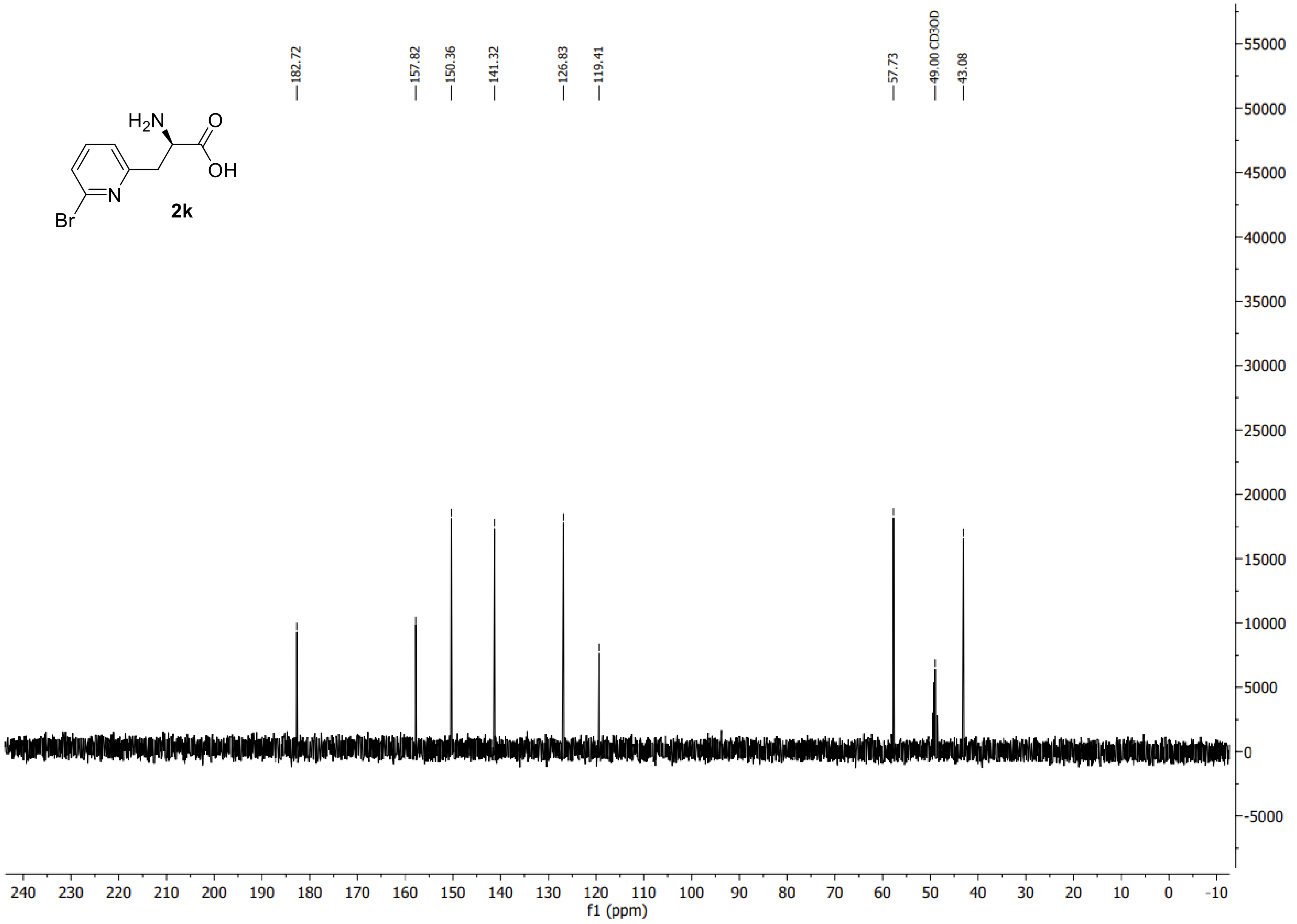

182.72
157.82
150.36
141.32
126.83
119.41
57.73
49.00 CD3OD
43.08
H2N
O
OH
N
Br
2k
f1 (ppm)

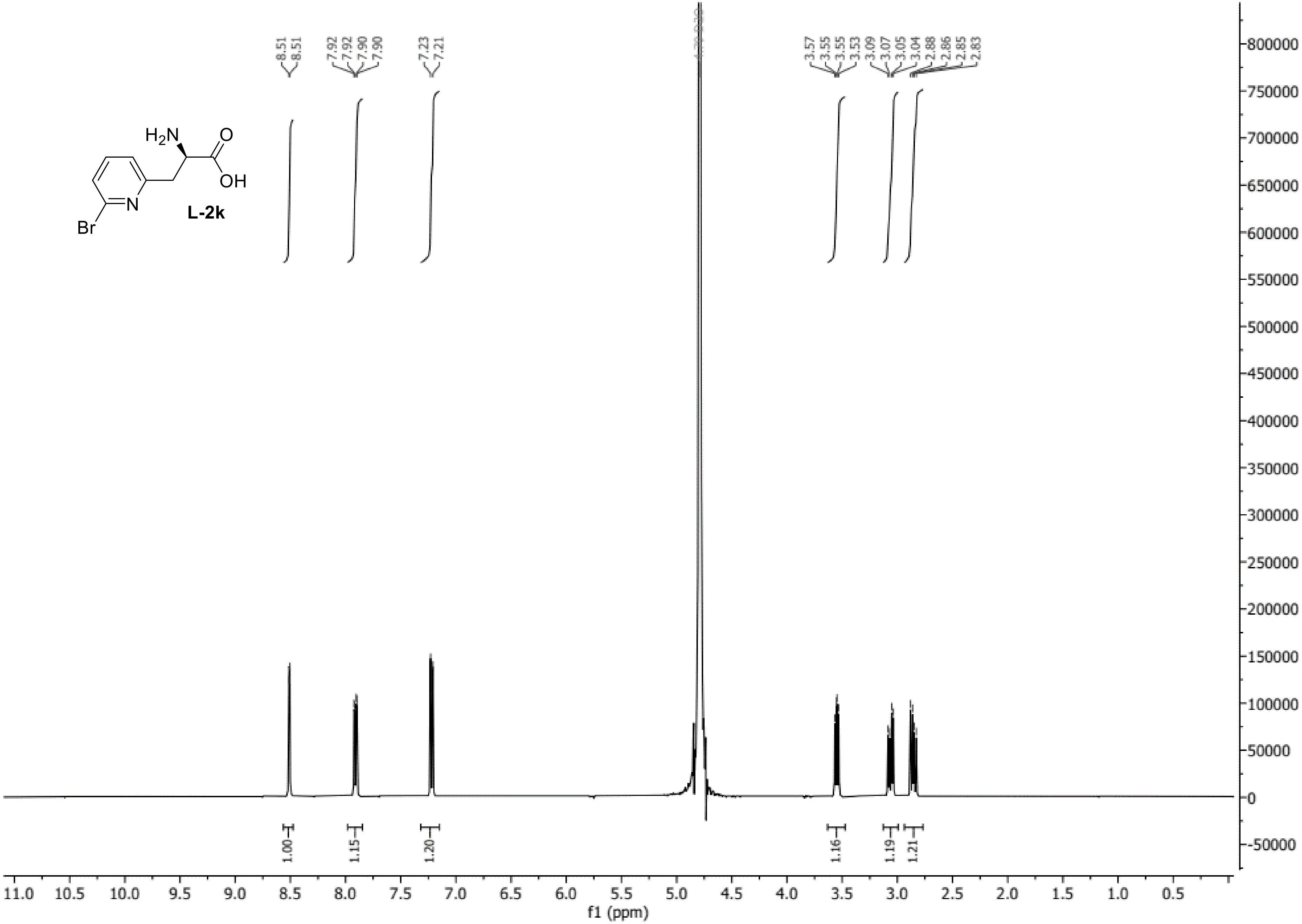
H2N
O
OH
N
Br
L-2k
8.51
8.51
7.92
7.92
7.90
7.90
7.23
7.21
3.57
3.55
3.55
3.53
3.09
3.07
3.05
3.04
2.88
2.86
2.85
2.83
1.00
1.15
1.20
1.16
1.19
1.21
800000
750000
700000
650000
600000
550000
500000
450000
400000
350000
300000
250000
200000
150000
100000
50000
0
-50000
11.0 10.5 10.0 9.5 9.0 8.5 8.0 7.5 7.0 6.5 6.0 5.5 5.0 4.5 4.0 3.5 3.0 2.5 2.0 1.5 1.0 0.5
f1 (ppm)

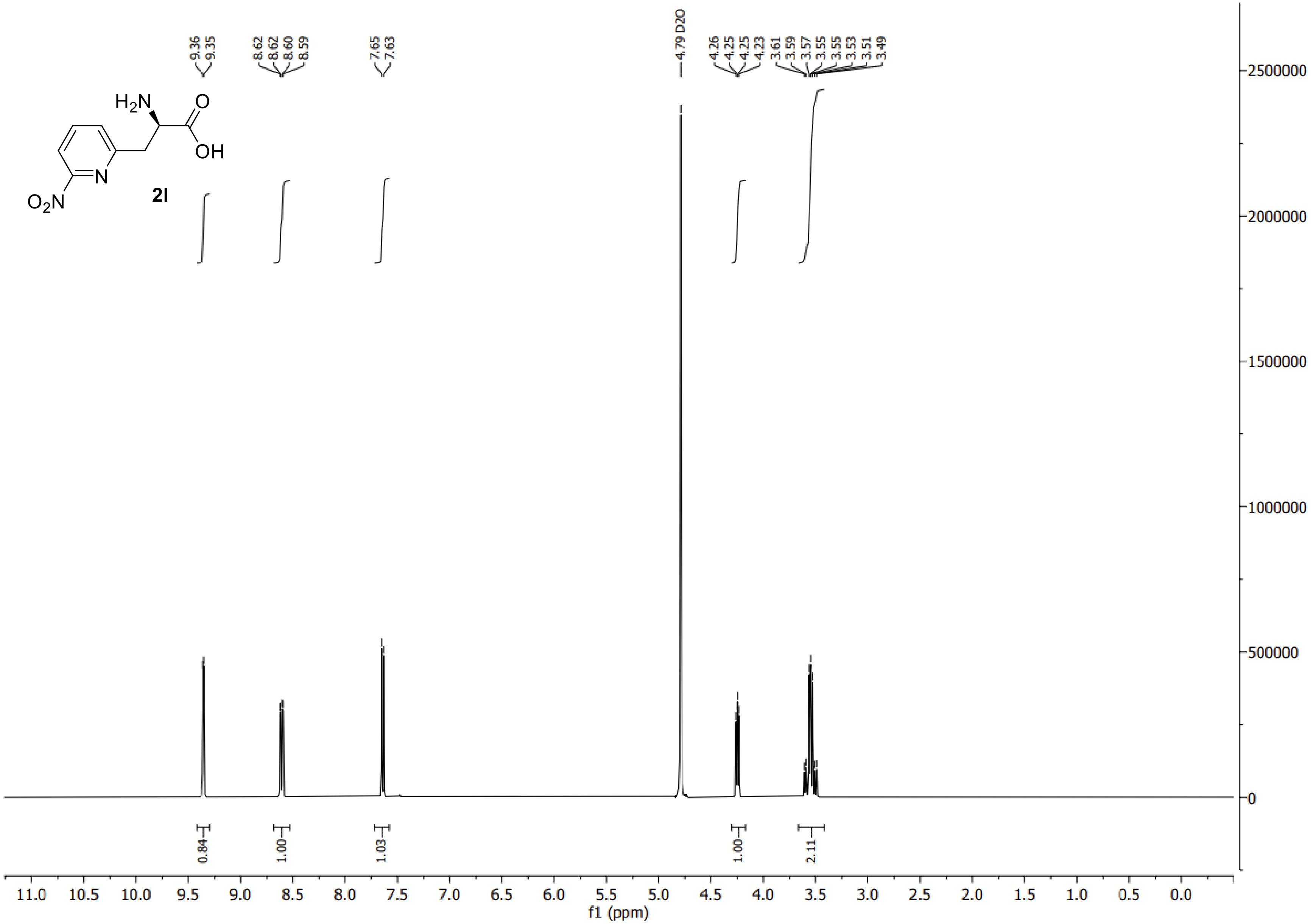
9.36
9.35
8.62
8.62
8.60
8.59
7.65
7.63
4.79 D2O
4.26
4.25
4.25
4.23
3.61
3.59
3.57
3.55
3.55
3.53
3.51
3.49
H2N
O
OH
N
O2N
2l
0.84
1.00
1.03
1.00
2.11
2500000
2000000
1500000
1000000
500000
0
11.0
10.5
10.0
9.5
9.0
8.5
8.0
7.5
7.0
6.5
6.0
5.5
5.0
4.5
4.0
3.5
3.0
2.5
2.0
1.5
1.0
0.5
0.0
f1 (ppm)

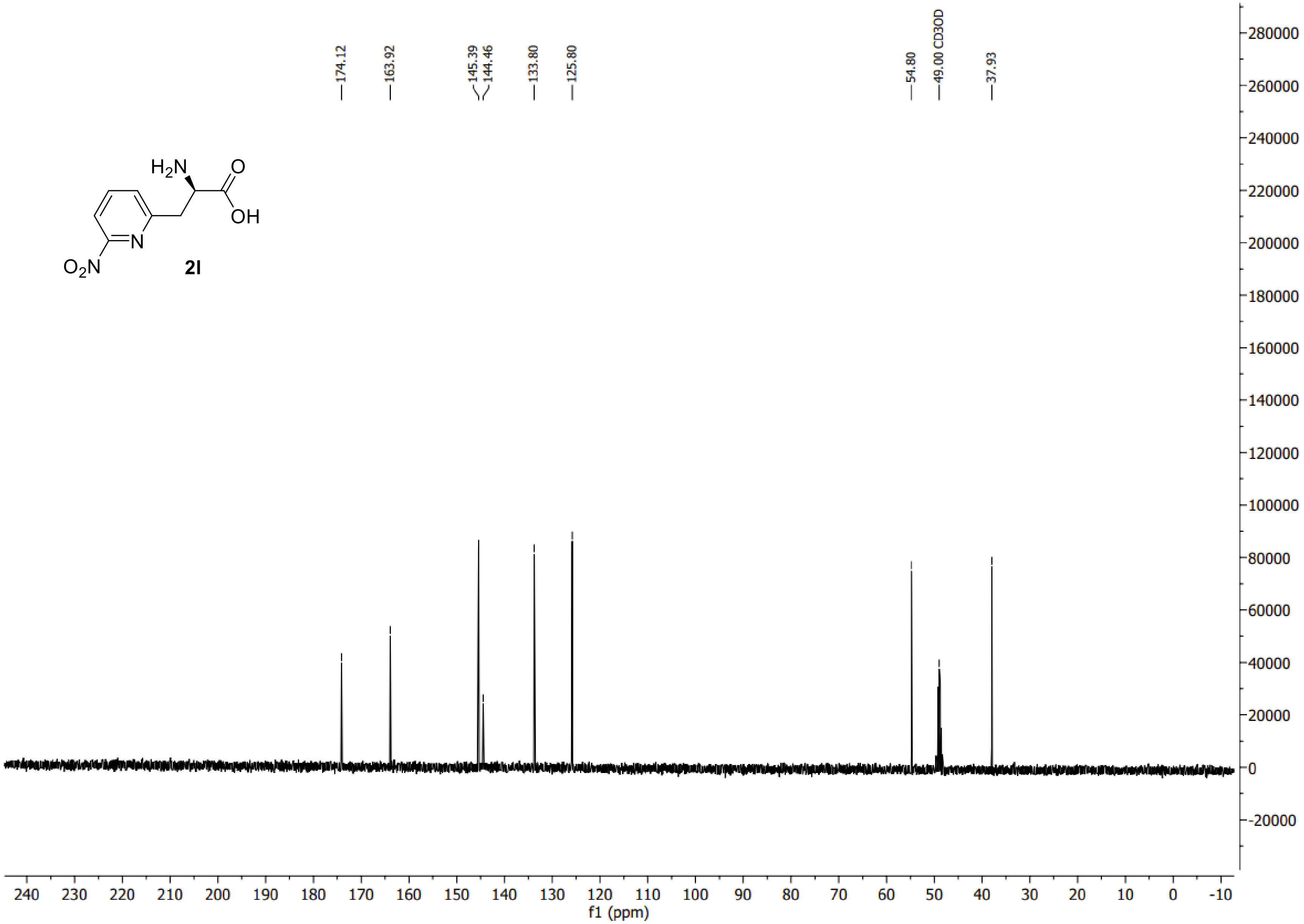
174.12
163.92
145.39
144.46
133.80
125.80
54.80
49.00 CD3OD
37.93
H2N
O
OH
N
O2N
2l
f1 (ppm)

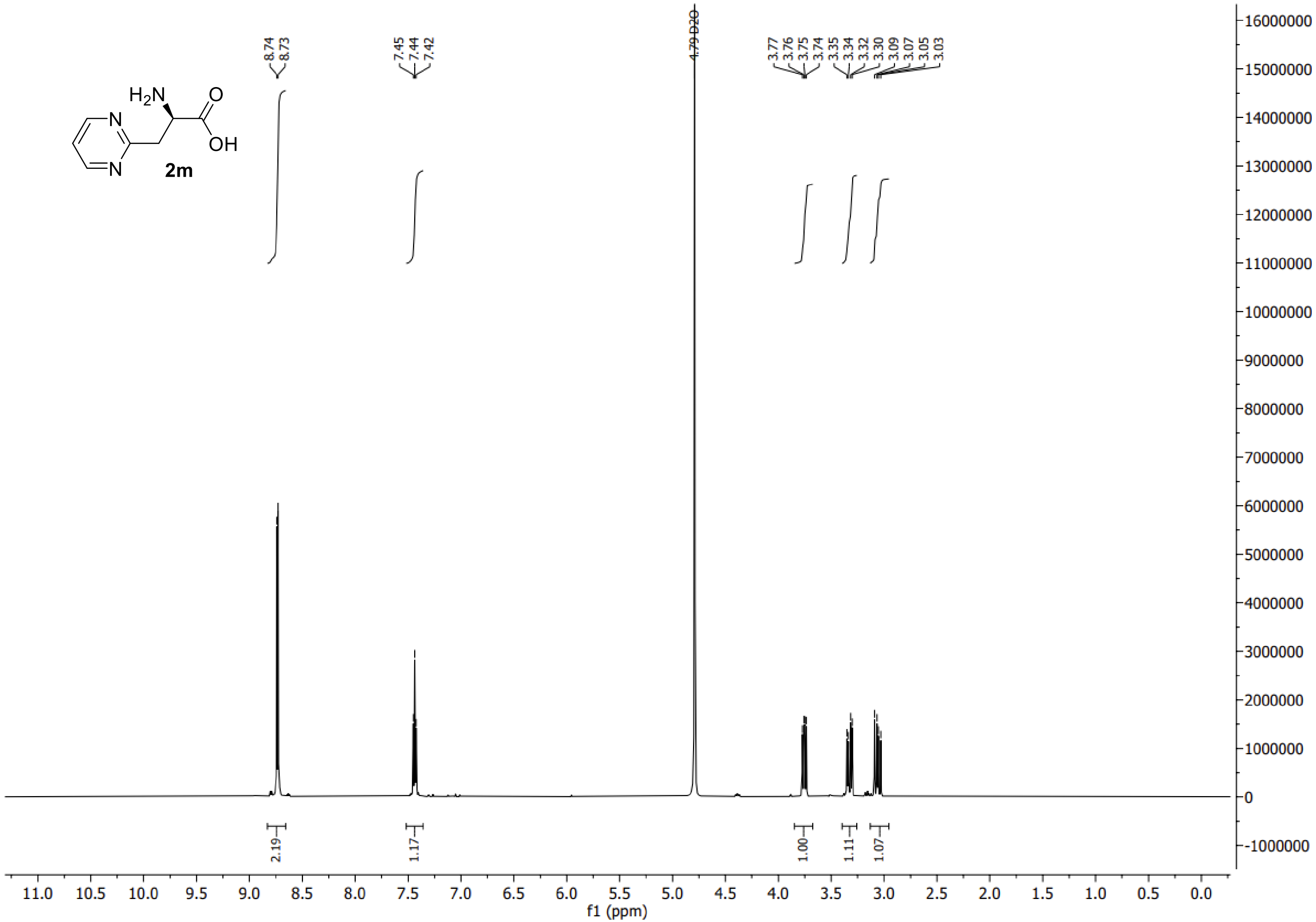

2m
8.74
8.73
7.45
7.44
7.42
4.79 D2O
3.77
3.76
3.75
3.74
3.35
3.34
3.32
3.30
3.09
3.07
3.05
3.03
2.19
1.17
1.00
1.11
1.07
f1 (ppm)

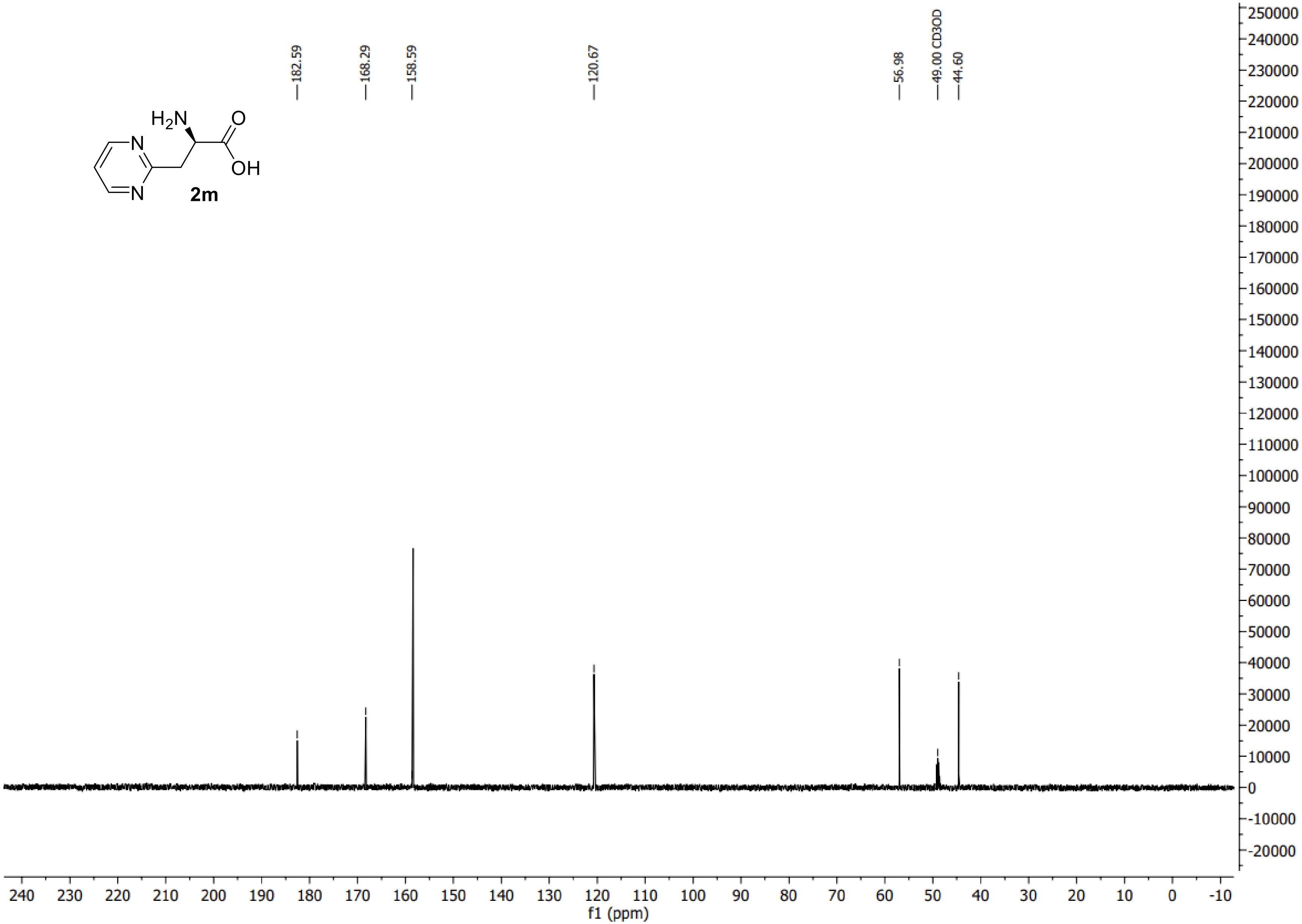
182.59
168.29
158.59
120.67
56.98
49.00 CD3OD
44.60
2m
f1 (ppm)

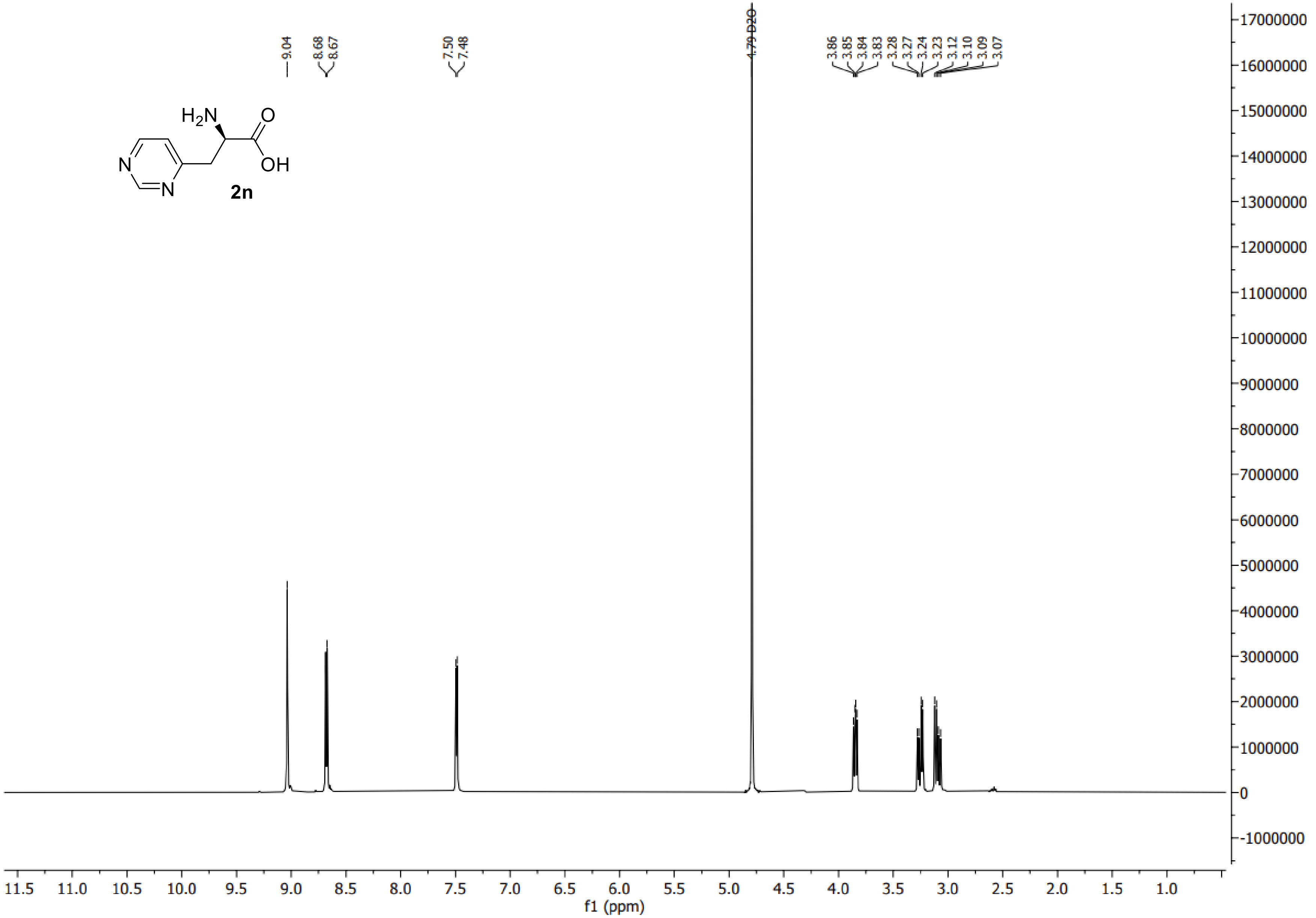
9.04
8.68
8.67
7.50
7.48
4.79 D2O
3.86
3.85
3.84
3.83
3.28
3.27
3.24
3.23
3.12
3.10
3.09
3.07
H_2N
O
OH
N
N
2n
f1 (ppm)

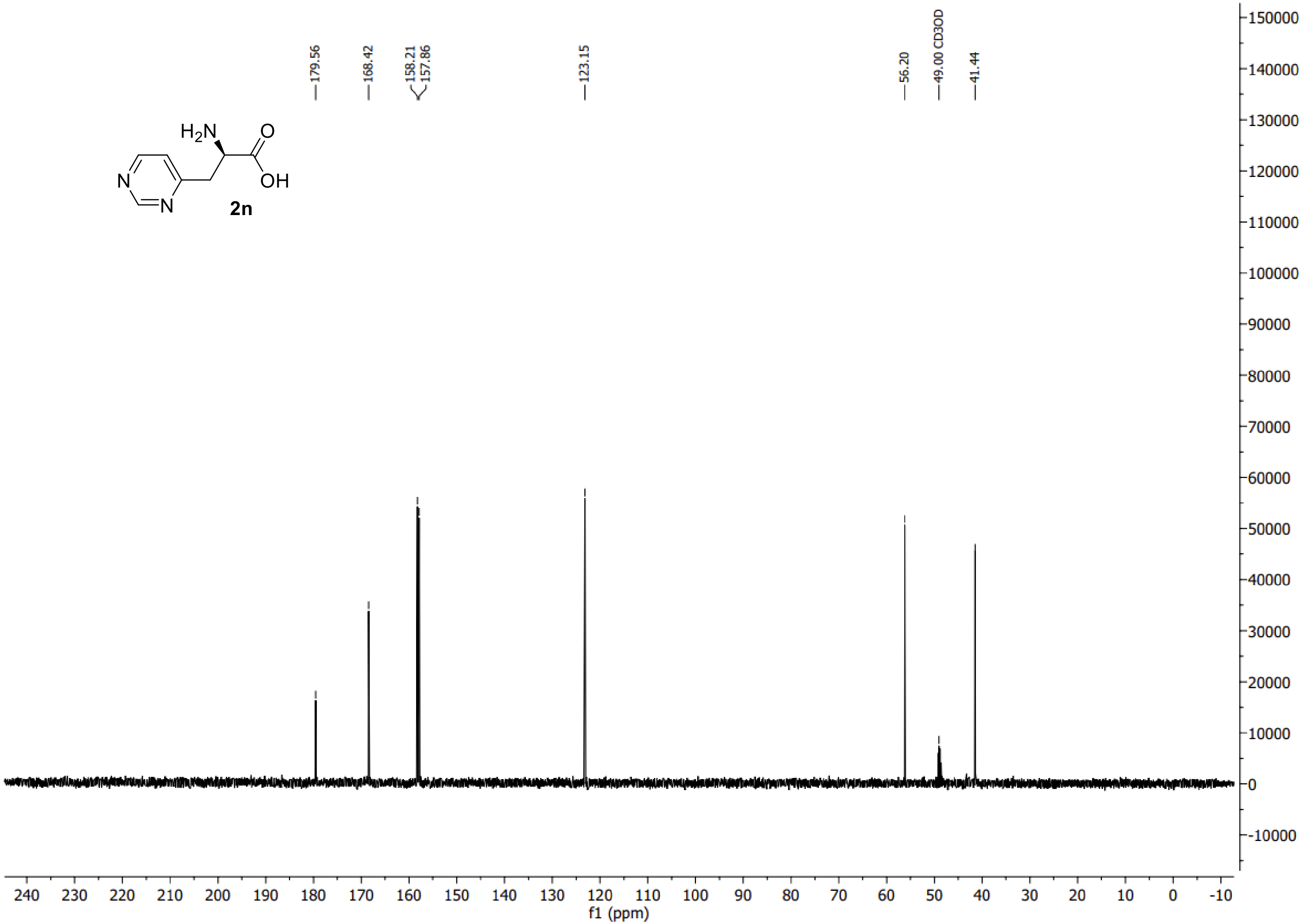
179.56
168.42
158.21
157.86
123.15
56.20
49.00 CD3OD
41.44
H2N
O
N
OH
N
2n
f1 (ppm)

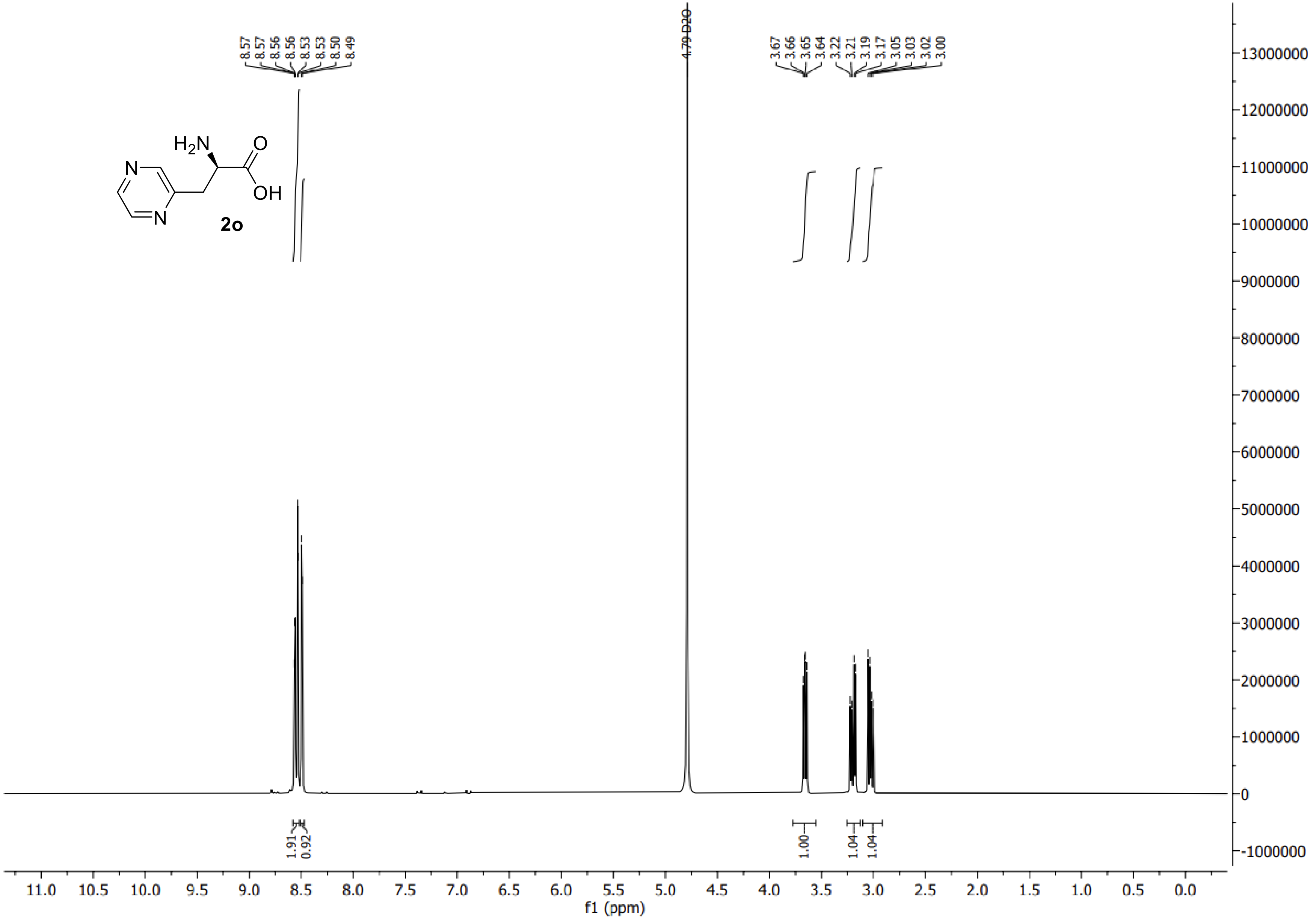
8.57
8.57
8.56
8.56
8.53
8.53
8.50
8.49
4.79 D2O
3.67
3.66
3.65
3.64
3.22
3.21
3.19
3.17
3.05
3.03
3.02
3.00
H2N
O
N
OH
N
2o
1.91
0.92
1.00
1.04
1.04
13000000
12000000
11000000
10000000
9000000
8000000
7000000
6000000
5000000
4000000
3000000
2000000
1000000
0
-1000000
11.0
10.5
10.0
9.5
9.0
8.5
8.0
7.5
7.0
6.5
6.0
5.5
5.0
4.5
4.0
3.5
3.0
2.5
2.0
1.5
1.0
0.5
0.0
f1 (ppm)

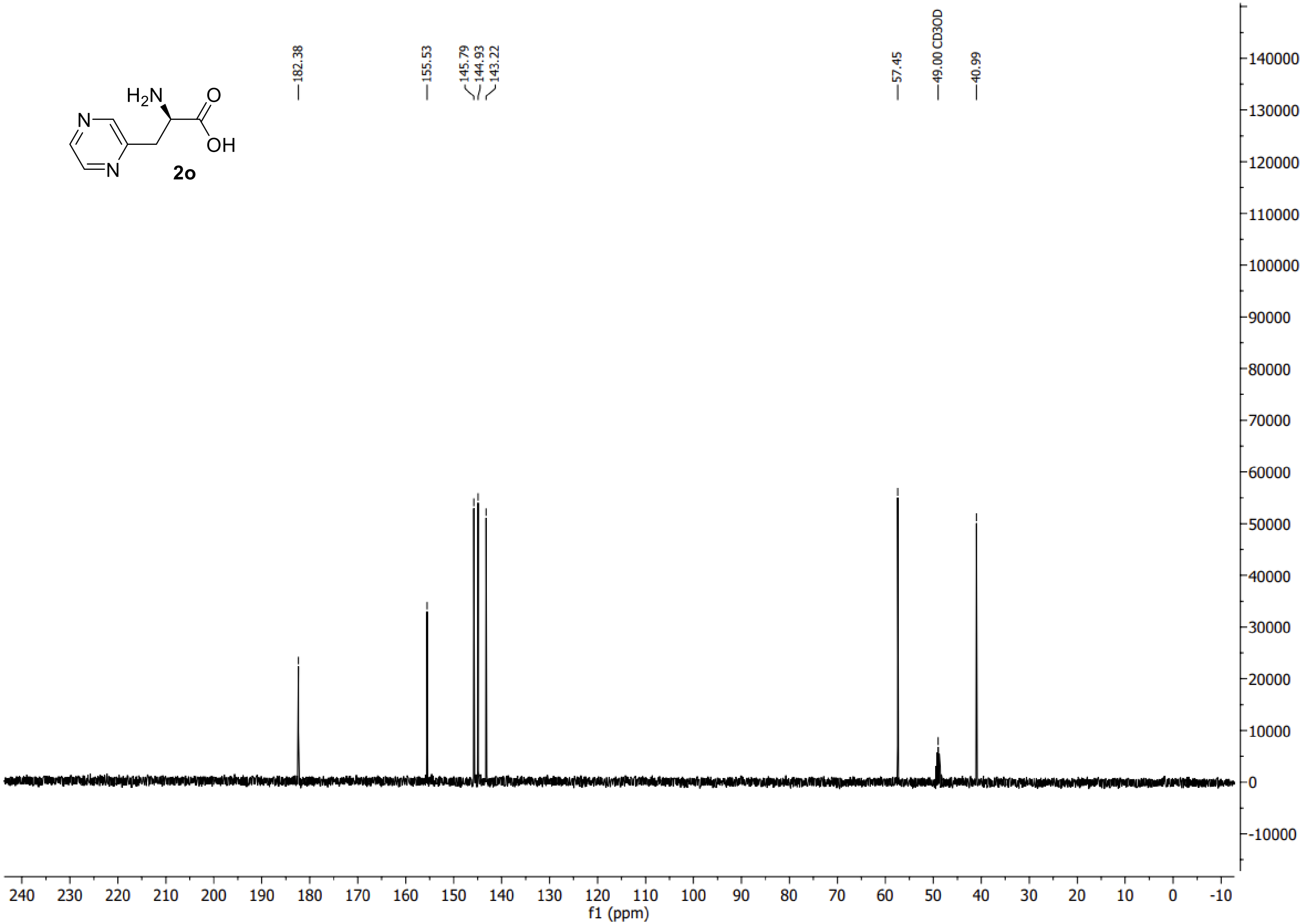

H2N
O
N
OH
N
2o
182.38
155.53
145.79
144.93
143.22
57.45
49.00 CD3OD
40.99
140000
130000
120000
110000
100000
90000
80000
70000
60000
50000
40000
30000
20000
10000
0
-10000
240
230
220
210
200
190
180
170
160
150
140
130
120
110
100
90
80
70
60
50
40
30
20
10
0
-10
f1 (ppm)

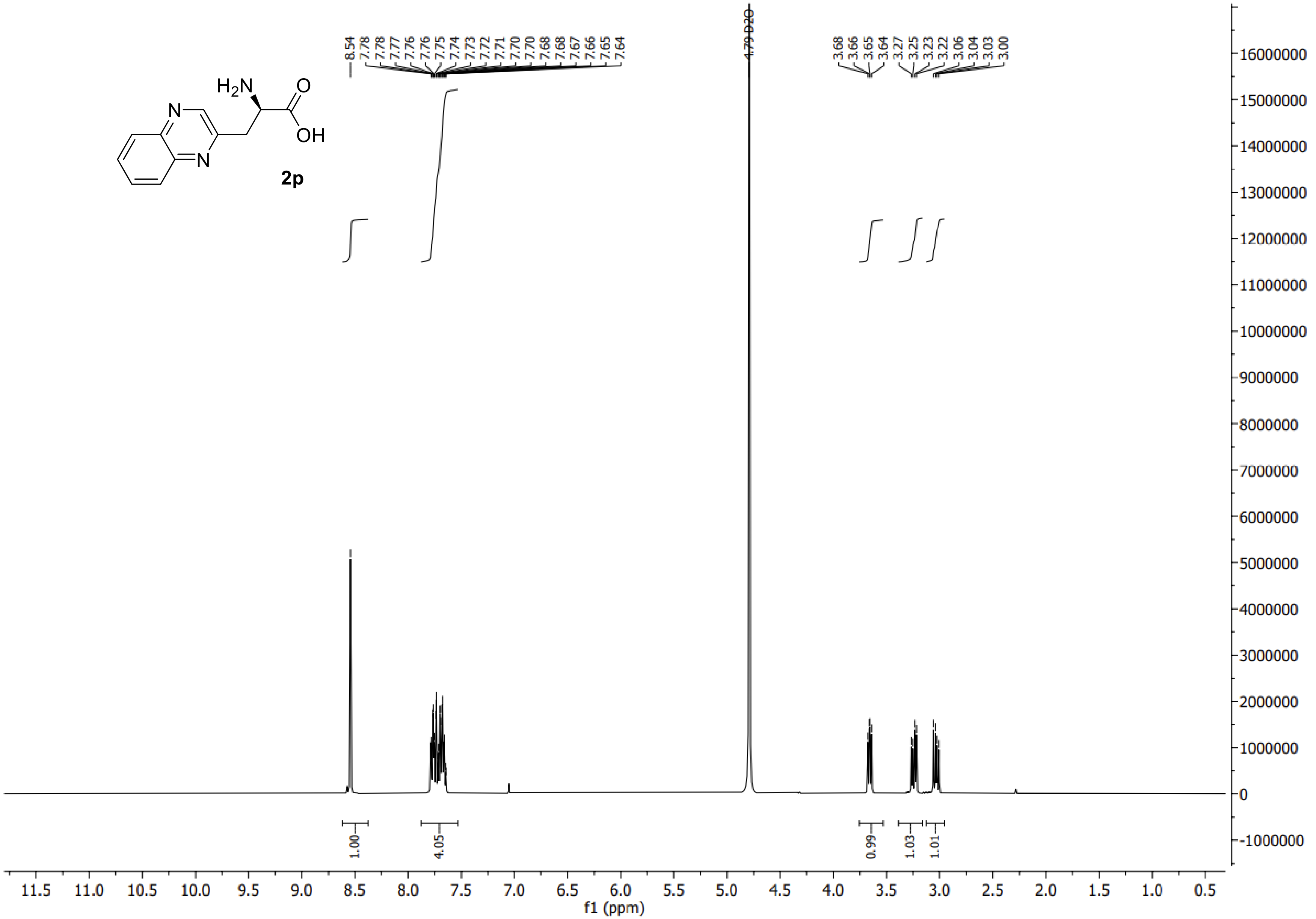
H2N
O
N
OH
N
2p
8.54
7.78
7.78
7.77
7.76
7.76
7.75
7.74
7.73
7.72
7.71
7.70
7.70
7.68
7.68
7.67
7.66
7.65
7.64
4.79 D2O
3.68
3.66
3.65
3.64
3.27
3.25
3.23
3.22
3.06
3.04
3.03
3.00
1.00
4.05
0.99
1.03
1.01
f1 (ppm)

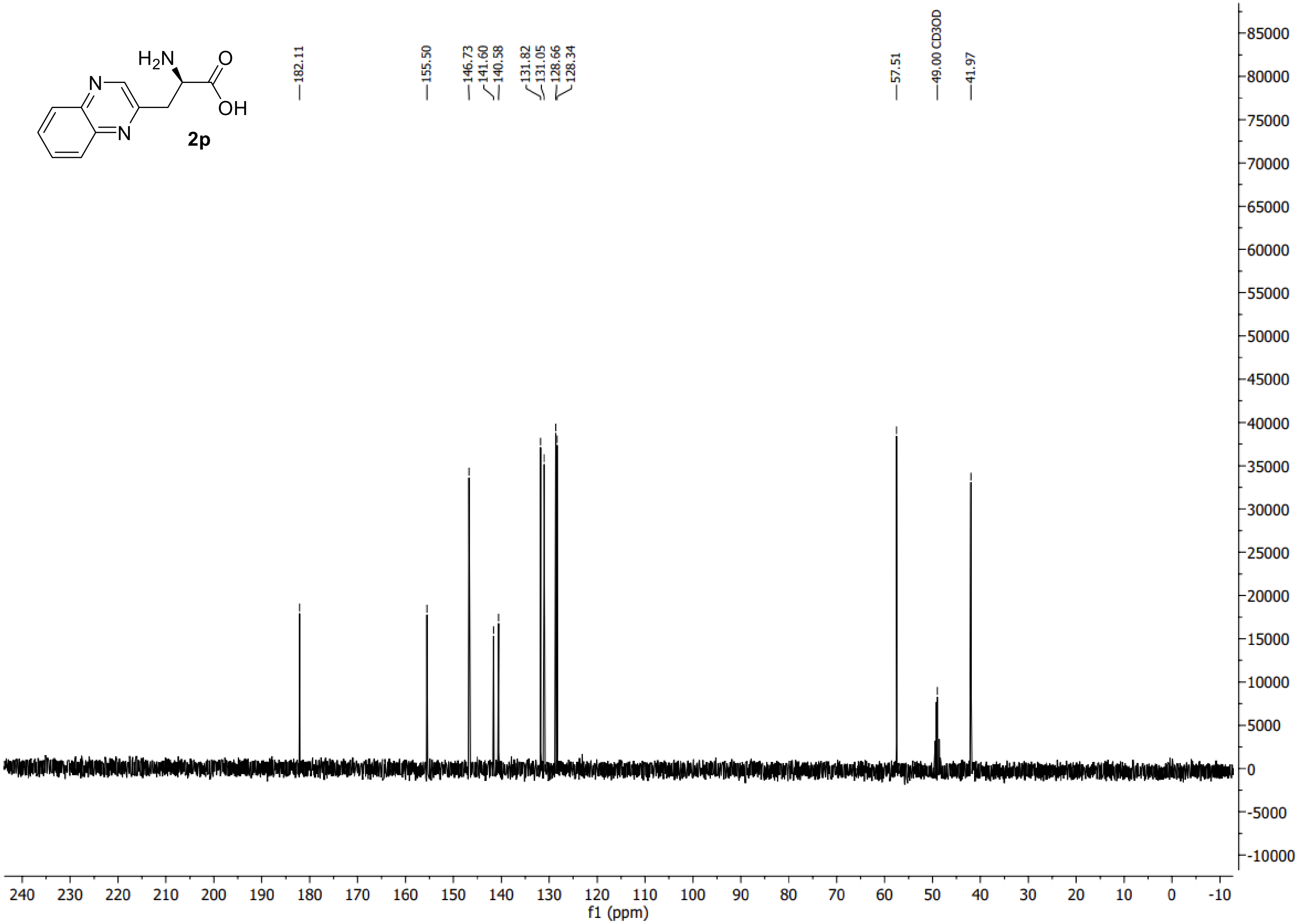

H2N
O
N
OH
N
2p
182.11
155.50
146.73
141.60
140.58
131.82
131.05
128.66
128.34
57.51
49.00 CD3OD
41.97
85000
80000
75000
70000
65000
60000
55000
50000
45000
40000
35000
30000
25000
20000
15000
10000
5000
0
-5000
-10000
240 230 220 210 200 190 180 170 160 150 140 130 120 110 100 90 80 70 60 50 40 30 20 10 0 -10
f1 (ppm)
